# Functional analysis of C1 family cysteine peptidases in the larval gut of *Тenebrio molitor* and *Tribolium castaneum*

**DOI:** 10.1186/s12864-015-1306-x

**Published:** 2015-02-14

**Authors:** Alexander G Martynov, Elena N Elpidina, Lindsey Perkin, Brenda Oppert

**Affiliations:** Department of Biomedical Science and Technology, Skolkovo Institute of Science and Technology, Skolkovo, 143025 Russia; Faculty of Bioengineering and Bioinformatics and A.N. Belozersky Institute of Physico-Chemical Biology, Moscow State University, Moscow, 119991 Russia; A.N. Belozersky Institute of Physico-Chemical Biology, Moscow State University, Moscow, 119991 Russia; USDA Agricultural Research Service, Center for Grain and Animal Health Research, Manhattan, KS 66502 USA

**Keywords:** High throughput sequencing, Cysteine peptidases, Cathepsin L, Cathepsin B, Peptidase homologs, *Tenebrio molitor*, *Tribolium castaneum*

## Abstract

**Background:**

Larvae of the tenebrionids *Tenebrio molitor* and *Tribolium castaneum* have highly compartmentalized guts, with primarily cysteine peptidases in the acidic anterior midgut that contribute to the early stages of protein digestion.

**Results:**

High throughput sequencing was used to quantify and characterize transcripts encoding cysteine peptidases from the C1 papain family in the gut of tenebrionid larvae. For *T. castaneum*, 25 genes and one questionable pseudogene encoding cysteine peptidases were identified, including 11 cathepsin L or L-like, 11 cathepsin B or B-like, and one each F, K, and O. The majority of transcript expression was from two cathepsin L genes on chromosome 10 (LOC659441 and LOC659502). For cathepsin B, the major expression was from genes on chromosome 3 (LOC663145 and LOC663117). Some transcripts were expressed at lower levels or not at all in the larval gut, including cathepsins F, K, and O. For *T. molitor,* there were 29 predicted cysteine peptidase genes, including 14 cathepsin L or L-like, 13 cathepsin B or B-like, and one each cathepsin O and F. One cathepsin L and one cathepsin B were also highly expressed, orthologous to those in *T. castaneum*. Peptidases lacking conservation in active site residues were identified in both insects, and sequence analysis of orthologs indicated that changes in these residues occurred prior to evolutionary divergence. Sequences from both insects have a high degree of variability in the substrate binding regions, consistent with the ability of these enzymes to degrade a variety of cereal seed storage proteins and inhibitors. Predicted cathepsin B peptidases from both insects included some with a shortened occluding loop without active site residues in the middle, apparently lacking exopeptidase activity and unique to tenebrionid insects. Docking of specific substrates with models of *T. molitor* cysteine peptidases indicated that some insect cathepsins B and L bind substrates with affinities similar to human cathepsin L, while others do not and have presumably different substrate specificity.

**Conclusions:**

These studies have refined our model of protein digestion in the larval gut of tenebrionid insects, and suggest genes that may be targeted by inhibitors or RNA interference for the control of cereal pests in storage areas.

**Electronic supplementary material:**

The online version of this article (doi:10.1186/s12864-015-1306-x) contains supplementary material, which is available to authorized users.

## Background

The tenebrionids *Tribolium castaneum* (red flour beetle) and *Tenebrio molitor* (yellow mealworm) are pests of cereal grains and stored products. Over 20 years of combined research by our group and others have revealed many similarities in the digestive processes of these insects, and yet they are distinct in physical parameters and developmental period (Additional file 1: Table S1). *T. molitor* larvae are up to five times larger and persist much longer than *T. castaneum* larvae, and yet *T. castaneum* adults live much longer than *T. molitor* adults. Biochemical studies in *T. molitor* are more abundant because of the larger size of the developmental stages. However, *T. castaneum* is more damaging economically worldwide, and genetic studies have facilitated it as a well-defined genetic model [1].

The C1 (papain) family is part of clan CA of cysteine peptidases containing catalytic Cys25 (hereinafter papain numbering) and His159 residues in the active site [2]. Two other active site residues are important to function: Gln19 is believed to help in the formation of the ‘oxyanion hole’, and Asn175 helps to orientate the imidazolium ring of the catalytic His [3]. Cysteine peptidases serve important functions in most life forms, but they also contribute to pathologies in humans, such as osteoporosis and cancer [4].

A large subset of C1 family cysteine peptidases is lysosomal, described mostly in mammals and human [5]. Human lysosomal cysteine peptidases have catalytic and inhibition mechanisms similar to papain, but they have differences in substrate specificity [6]. The S2 substrate binding subsite is a determining factor in the specificity of C1 family peptidases and is the only real pocket on the protein surface. Large hydrophobic residues in the P2 position and positively charged residues in the P1 position of the substrate are usually preferred. However, cathepsin B is unique in that it will also accept an arginine residue in the P2 position due to a Glu residue (Glu205) in the S2 subsite [7].

The importance of digestive cysteine peptidases in insect pests has been described in beetles from the infraorder Cucujiformia, some hemipterans, and thrips [8-11]. For tenebrionids, protein digestion is a compartmentalized process that heavily relies on cysteine peptidases due to a pH gradient that regulates enzyme activity, with cysteine peptidases mostly in the acidic anterior midgut due to their acidic pH optima [12-16]. While cysteine peptidases provide two-thirds of the total proteolytic activity in the *T. molitor* larval anterior midgut [14], *T. castaneum* larval cysteine peptidases contribute up to 97% of the total anterior midgut proteolytic activity [17].

Biochemical studies of digestive peptidases in *T. molitor* larvae have identified at least six fractions of cysteine peptidase activities [14,16,18] with the major activity from cathepsin L [19]. In *T. molitor,* cysteine peptidases also are important in processing the major glutamine-rich dietary proteins in cereals, prolamins, functioning as post-glutamine hydrolyzing enzymes [20]. At least eight fractions of cysteine peptidase activities were found in the *T. castaneum* larval gut [17,21].

During the annotation of the *T. castaneum* genome, we identified 25 potential cysteine peptidase genes, including cathepsins B, K, L, and O, and some were predicted to be inactive homologs due to a lack of sequence conservation in critical conserved residues [1]. Four linkage groups containing cysteine peptidase gene expansions were established from phylogenetic analysis of predicted *T. castaneum* cysteine cathepsin genes and related sequences in other species. Many of the cathepsin B and L peptidases are expressed in the *T. castaneum* larval gut to varying levels, according to gene expression microarrays [21].

To further study the expression and activity profile of cysteine peptidases in tenebrionid larvae, we accumulated new sequencing data from the midgut of *T. molitor* and *T. castaneum*. These data allow us to refine our model of C1 peptidase genes and predict relative transcript expression in each insect. Furthermore, we have used modeling and substrate docking to speculate on the peptidase structure relative to function in the larval gut of each insect species. These data provide the most comprehensive dataset for coleopteran digestive peptidases to date. In the analysis of peptidase structures, we describe for the first time a new subset of C1 peptidases.

## Methods

### Preparation of biological material and sequencing of cDNA

The Center for Grain and Animal Health Research (CGAHR, Manhattan, KS USA) has laboratory colonies of *T. molitor* maintained on a diet of 50% oat flakes, 2.5% brewer’s yeast, and 47.5% wheat flour at 28°C, 75% R.H., in darkness. Approximately five week old larvae with an average weight of 5.1 mg from three independent biological replicates were fasted overnight and were placed on a diet consisting of 85% stabilized wheat germ, 10% wheat flour, and 5% brewer's yeast for 12 h. For each replicate, the midgut was extracted from 4-7 larvae and placed in room temperature RNAlater (Ambion, Austin TX USA). For RNA isolation, excess RNAlater was blotted, and pooled midguts were ground with a plastic pestle in 1.5 ml microfuge tube containing liquid nitrogen. Total RNA was isolated using the Absolutely RNA Kit with DNase on-column treatment (Agilent Technologies, La Jolla, CA USA). The resulting total RNA was sent to a sequencing facility (National Center for Genome Resources - NCGR, Santa Fe, NM, USA), where mRNA was isolated by polyA, standard libraries were made, and paired-end sequencing was performed on a Illumina HiSeq 2000 (San Diego, CA, USA) using standard protocols from the manufacturer. We obtained approximately 240 million sequence reads, with an approximate 250 bp insert.

*T. castaneum* were reared at CGAHR on a diet of 95% wheat flour and 5% brewer's yeast at 28°C, 75% R.H., in darkness. Neonate larvae from three independent biological replicates were placed on 85% stabilized wheat germ, 10% wheat flour, and 5% brewer's yeast and reared under normal rearing conditions for 14 d. Total RNA was extracted as with *T. molitor* (above). Sequencing of cDNA was by the High Throughput Genomics Center, Seattle, USA, and paired-end sequencing on the Illumina HiSeq 2000. We obtained 344,476,216 sequence reads, >95.6% = Q30 post filtering, with a mean quality score of 37.39, and insert size approximately 250 bp.

### Assembly of contigs

A custom assembly of *T. molitor* sequences combined from all replicates was made by NCGR, resulting in 197,800 contigs (minimun length = 100 and maximum length = 51,328; Q1 = 123, Q2 = 153, Q3 = 335; N50 = 2232, B1000 = 71.9%, B2000 = 54.1%). For *T. molitor* sequences, we also combined the replicate data and included previous databases of Sanger sequencing [16] and pyrosequencing [22] of mRNA from the larval gut and performed additional *de novo* assemblies with SeqManNGen (v. 4.0.1.4, DNAStar, Madison, WI USA) and custom assembly programs. For *T. castaneum* sequences, we used SeqManNGen to map sequences to the *T. castaneum* genome (Tcas3, NCBI; parameters for alignment were merSize = 19; 309,572,610 bp submitted, 263,305,494 aligned, 17,268,742 unaligned; sequence count score was > 90%), as well as Galaxy [23,24]. Potential coding sequences, starting at methionine and covering at least 20% of the mRNA sequence, were found in the *T. molitor* contigs using custom software.

### Analysis of sequences

BLAST [25] and custom scripts were used to identify ORFs homologous to those encoding cysteine peptidases from the C1 papain family [26]. Two cysteine peptidase sequences from the gut of *T. molitor* were used as query sequences (Cont-08879 and Cont-00890) [27]. We constructed multiple alignments from pairwise alignments of predicted protein sequences, using custom scripts based on the algorithm of Wagner-Fischer [28]. ORFs that were grouped into blocks with identity of at least 95% and that overlapped with another block of at least 10 amino acid residues were considered as referring to one unique peptidase. Multiple sequence alignment (MAFFT) [29] and custom scripts were used to refine and build consensus sequences, and in the case of SNPs, the amino acid chosen was the highest percentage and more than 50% of the total. We used SignalP 4.0 [30] to detect signal peptides and identify the predicted start of a translated sequence. Final alignments of sequences were made with MegAlign (MUSCLE, DNAStar) [31] to compare functional and conserved residues in peptidases. In addition, the predicted start of the *T. molitor* and *T. castaneum* mature enzyme sequence was identified by sequence homology through alignment with mature human cathepsin L and cathepsin B.

### Modeling and visualization of three-dimensional structures of enzymes: analysis of substrate binding subsites

The simulation of 3D structures of cysteine cathepsin proteins was obtained by two different approaches:

1. Homologous modeling of the predicted mature enzyme with a ligand (inhibitor) was with Modeller [32]. The following structures were used:
  mature human cathepsin L complexed with a peptide inhibitor (3OF8) [33] to model cathepsin L-like sequences;
  mature rat cathepsin B with a peptide inhibitor (1THE) [34] - for the simulation of cathepsin B-like sequences.
2. Homologous modeling of tertiary structures of the proenzyme and the mature enzyme was with RaptorX [35,36] using a multi-patterned approach to the modeling of the tertiary structure of the enzyme.

Both methods resulted in similar structures; therefore, method 2 was used for analysis. Model quality was evaluated by Ramachandran plots using RAMPAGE [37]. Structures were discarded and were not included in further analyses if the percentage of residues in a favored region was lower than 90%, or amino acids of the active site or S2 subsite were in an outlier region.

Amino acids corresponding to S1 and S2 subsites in *T. molitor* and *T. castaneum* were identified by comparison to model sequences using ClustalW [38] and structural comparison of 3D models of mature peptidases with crystal structures of human mature peptidases by PDBeFold [39]. PyMol software [40] was used for visualization of the resulting structures. In addition, the models of mature peptidases were compared with crystal structures of corresponding mutant procathepsins 3QJ3 and 3QT4 [41] using PDBeFold [39].

### Modeling and analysis of enzyme-substrate complexes

Docking was used to model the enzyme-substrate complexes of cysteine peptidases and classical substrates of cathepsins, the tripeptides phenylalanine-arginine-phenylalanine (FRF) and lysine-arginine-phenylalanine (LRF). 3D structures of substrates were obtained by ChemSketch [42] using spatial optimization, and were secondarily optimized by MOPAC [43]. Docking was performed by AutoDock [44], using standard docking parameters, except for the number of conformations (number of GA runs), which varied from 700 to 1000. Using a custom script and visual analysis of the models, we ensured that they met the following criteria that are required for hydrolysis of the proper bond:

- The distance between the C atom of arginine and the cysteine S atom of the active center should not exceed 4 Å;
- The orientation of the substrate shall be as described in the literature: N-terminal phenylalanine must be located in the subsite S2, and C-terminal should be in the S1’ subsite;
-NH_2_ and-COOH groups of the substrate must be sterically accessible and not be immersed in the enzyme.

### Analysis of expression

To analyze the expression of peptidase transcripts in *T. castaneum*, we used reads mapped to the genome of *T. castaneum* (Tcas3) to obtain expression values for contigs by normalized reads per kilobase per million mapped reads (RPKM) [45], using SeqManPro (DNAStar).

For *T. molitor*, about 40 million reads were assembled from each of three datasets of RNA-Seq data. BLAST, MAFFT, and custom scripts were used to obtain all nucleotide sequences in the contigs potentially encoding peptidases. The contigs were used to assemble and refine sequences of complete peptidase mRNAs. Refined peptidase mRNA sequences were used to identify contigs in each assembly with at least 97% sequence identity, which were used for expression calculations. If a contig aligned only partially to the mRNA, its contribution to expression was proportional to the aligned part if it was more than 50% of the contig length. RPKM was used to calculate the number of reads mapped to a contig, calculating each multiread as one unit. As an additional approach, we used a “rescue” method [46]. The rescue method calculates similarly the number of unique reads aligned to a contig, but the multi-mapped reads were counted fractionally proportional to the number of different map sites, the expression level and the length of the contig. Without a sequenced genome for *T. molitor,* the method of rescue probably gives a better estimate of mRNA expression, because repetitive and overlapping contigs may have been overrepresented, and calculation of each multi-mapped read as one unit can give an inflated expression. To compare expression levels within each insect, we took the RPKM for each predicted peptidase and divided by the sum of RPKM for all peptidase transcripts × 100.

### Phylogenetic analysis and orthology predictions

A cladogram was constructed with MegAlign, using ClustalW alignment [38,47] according to Dayhoff et al. [48], and bootstrapping = 1,000, seed = 111 (DNAStar). Cathepsin K from *T. castaneum* was used as the outgroup for the tree. Based on the cladogram, pairs of sequences from *T. molitor* and *T. castaneum* were considered orthologous if they formed a single clade. If more than two sequences formed a clade that could not be separated into orthological pairs, this set of sequences was considered an orthological group.

## Results

Using different algorithms to analyze RNASeq data, we were able to predict cysteine peptidase genes in sequences from the larval gut of *T. molitor* and *T. castaneum* that were similar to peptidases from the C1 papain family, and calculate relative expression values. Different approaches were taken because of the availability of a reference genome for *T. castaneum* and lack of a sequenced genome for *T. molitor.* These comparisons identified similarities and differences in the complement of C1 cysteine peptidases in the two tenebrionids.

### Cysteine peptidases in the *T. castaneum* larval gut

In our previous bioinformatic study of cysteine peptidases in the *T. castaneum* genome as part of the annotation project, 24 genes were identified that encode enzymes similar to the C1 papain family peptidases [1]. We now update this to 25 putative cysteine peptidase genes and one pseudogene, found on chromosomes 3 (five), 7 (seven and one pseudogene), 8 (six), and 10 (five) (Table 1). Previous annotations of Tc01950 and Tc09363 have been removed from consideration (although Tc01950 remains in the unlocated contigs at NCBI, we believe that it duplicates NP_001164001). These genes have been tentatively classified as: 10 encoding cathepsin L and one inactive homolog (lacking conservation in active site residues of peptidases); three cathepsin B, six similar to cathepsin B (cathepsin B-like) and two cathepsin B inactive homologs; one each cathepsin F, K, and O. Cathepsins F, K and O are found on chromosomes 7, 1(X) and 4, respectively.

**Table 1:** **Predicted cysteine cathepsin genes (B, L, O, K, and F) in the** ***T. castaneum*** **genome, and relative expression levels in the larval gut, as estimated by transcriptome and microarray data**

**Protein ID**	**Gene ID**	**Tc annotation** ^**1**^	**Chromosome**	**Expression (RPKM)**	**Gut rank** ^**2**^	**Active site residues** ^**3**^	**NCBI annotation**
NP_001164001	LOC659441	11001	10	77,228.22	82	QCHN	cathepsin L
NP_001164314	LOC659502	11000	10	25,848.86	14	QCHN	cathepsin L
XP_970644	LOC659226	11003	10	42.77	7	QCHN	cathepsin L
XP_970773*	LOC659367	11002	10	35.06	2	ESHN	cathepsin L homolog
XP_970951	LOC659565	10999	10	0.28	2	QCHN	cathepsin L
XP_971698	LOC660368	09365	7	2387.62	44	QCHN	cathepsin L
XP_971867	LOC660551	09362	7	1.99	1	QCHN	cathepsin L
XP_971752	LOC660428	09364	7	0.98	1	QCHN	cathepsin L
XP_971975	LOC660669	09448	7	0.02	2	QCHN	cathepsin L
NC_007422	LOC660491	pseudogene?	7	-	NOC		-
XP_974298	LOC663145	02952	3	3,142.15	43	QCHN HH	cathepsin B
NP_001164205	LOC663117	02953	3	1,132.96	46	QCHN HH	cathepsin B
XP_974244	LOC663090	02954	3	248.71	9	QCHN HH	cathepsin B
XP_974220	LOC663066	02955	3	79.91	2	QCHN	cathepsin B-like
XP_966750	LOC655148	05431	8	443.27	3	QCHN	cathepsin B-like
XP_966663	LOC655077	05432	8	1.18	3	QCHN	cathepsin B-like
XP_968689*	LOC657117	05954	8	57.60	NOC	QSTN	cathepsin B homolog
XP_968767	LOC657203	05953	8	82.17	3	QCHN	cathepsin B-like
XP_008196467^4^	LOC656957	05955/05956	8	-	NOC	QCHN	cathepsin B-like
XP_008196465^4^	LOC657038	---	8	-	NOC	QCHN	cathepsin B-like
NP_001164088	LOC663234 (26-29-p)	--- (09486)	7	1,309.16	NOC	QCHN	cathepsin L
XP_969833	LOC658343	02843	3	0.02	1	QCHN	cathepsin L
XP_967834* (XP_008195382)^5^	LOC656198	09217	7	28.64	1	QSHN	cathepsin B homolog
XP_970512	LOC659087	07214	4	11.99	NOC	QCHN	cathepsin O
XP_973607 (XP_3195656)^6^	LOC662417	---	7	2.32	NOC	QCHN	cathepsin F
XP_001814509	LOC100141668	13582	1 (X)	0	1	QCHN	cathepsin K

^1^From [1]. Tc09363 and Tc01950 were in the original annotation but have been removed from the annotations of cysteine cathepsins; Tc09486 was missed in the original annotation.

^2^As defined in [21], from microarray gene expression data from larval gut tissue (higher ranks=higher expression); NOC – not on chip.

^3^Active site residues including those in occluding loop [55].

^4^Changed in the Tcas4 genome build; listed as a pseudogene in Tcas3, and no expression values available.

^5^Now annotated by NCBI as tubulointerstitial nephritis antigen-like.

^6^Changed in Tcas4 genome build.

*Predicted homologs according to lack of sequence conservation in active site residues.

In the present study, some of the coding sequences in the current version of the *T. castaneum* genome (Tcas3) were supported by transcriptome data (Table 1); because the sequences were from gut tissue, these cysteine cathepsins are expressed in the larval gut. The majority of transcript expression was from two cathepsin L genes on chromosome 10 (LOC659441 and LOC659502), and two on chromosome 7 (LOC660368, 26-29-p); for cathepsin B, the major gut peptidase genes were on chromosome 3 (LOC663145 and LOC663117). The other cathepsin L and B peptidases, as well as cathepsin F, K, and O, have low transcript expression levels and do not appear to be important for the digestion of food. One pseudogene, LOC660491, had some associated reads, but we were unable to calculate RPKM due to its annotation in the genome; whether this is an actual pseudogene remains to be determined.

### Cysteine peptidases in the *T. molitor* larval gut

Sequence analysis of gut cDNA from *T. molitor* larvae revealed 29 predicted protein sequences similar to cysteine peptidases from the papain C1 family (Table 2). Of those, 14 sequences were similar to cathepsin L, of which two had substitutions in the conserved active site and were considered inactive homologs. There were transcripts encoding three cathepsin B, nine B-like peptidases, and one inactive cathepsin B homolog. We found one each cathepsin F and O, but no ortholog to cathepsin K, and cathepsin K is not expressed in the gut tissue of *T. castaneum* [21]. Ten of these sequences correspond to previously annotated sequences [16,19,27,41], but 19 are new and first described in this study.

**Table 2:** **Predicted cysteine cathepsin genes in the** ***T. molitor*** **genome, and relative expression levels in the larval gut, as estimated by transcriptome data**

**Identification**	**NCBI accession** ^**1**^	**Previous iterations (% Identity)**	**Expression (RPKM)**	**Expression (Rescue)**	**Active site residues** ^**7**^	**Predicted annotation**
TmL13	KP303287	AM4-22 (ABC88769, 99%), AM3-32 (ABC88768, 99%)^2^; TmCysII, TmCysIII^3^; ppCal3 (AAP94048)^4^; 3QT4^5^; Cont-08897, Bt-07583^5^	19,726.5	8,496.6	QCHN	cathepsin L
TmL5	KP303279	ppCAL2 (AAR05023, 97%)^4^; 3QJ3^5^; Cont-01354, Bt-07528^6^	1,356.6	572.7	QCHN	cathepsin L
TmL11	KP303285	Cont-00009, Bt-01497^6^	1,149.4	354.9	QCHN	cathepsin L
TmL2	KP303276	ppCAL1a,b,c (AAP94046, 100%)^4^; Cont-09057, Bt-00111^5^	337.3	263.5	QCHN	cathepsin L
TmL4	KP303278		326.9	168.9	QCHN	cathepsin L
TmL1	KP303275		162.2	113.9	QCHN	cathepsin L
TmL30*	KP303289		130.3	104.4	ESHN	cathepsin L homolog
TmL29*	KP303289		130.3	104.4	QAHN	cathepsin L homolog
TmL3	KP303277	AAP94047 (91%)^3^	62.1	31.3	QCHN	cathepsin L
TmL9	KP303283		72.0	46.0	QCHN	cathepsin L
TmL7	KP303281		25.3	11.7	QCHN	cathepsin L
TmL6	KP303280	Bt-07886	15.8	5.8	QCHN	cathepsin L
TmL8	KP303282		5.7	3.7	QCHN	cathepsin L
TmL15	KP303288		0.2	0.2	QCHN	cathepsin L
TmB33	KP303302	AM4-18 (ABC88766, 98%)^2^; TmCysII^3^; Cont-09310, Bt-002495	2,489.6	1,160.4	QCSN HH	cathepsin B
TmB20	KP303293	АМ3-87 (ABC88767, 99%)^2^; Cont-00890^6^	448.4	221.5	QCHN	cathepsin B-like
TmB25	KP303297		672.6	296.7	QCHN	cathepsin B-like
TmB26	KP303298	Cont-08975, Bt-08237^6^	657.5	431.6	QCHN	cathepsin B-like
TmB18	KP303291		283.2	175.8	QCHN HH	cathepsin B
TmB17	KP303290	Cont-00240, Bt-01453^6^	163.9	99.0	QCHN HH	cathepsin B
TmB32*	KP303301		77.9	37.5	QSHN	cathepsin B homolog
TmB23	KP303295		48.8	29.3	QCHN	cathepsin B-like
TmB19	KP303292		34.2	24.9	QCHN	cathepsin B-like
TmB27	KP303299		26.5	13.9	QCHN	cathepsin B-like
TmB24	KP303296		20.0	6.5	QCHN	cathepsin B-like
TmB28	KP303300		4.0	2.2	QCHN	cathepsin B-like
TmB22	KP303294		1.2	0.7	QCHN	cathepsin B-like
TmO12	KP303286		38.4	22.2	QCHN	cathepsin O
TmF10	KP303284		23.4	8.4	QCHN	cathepsin F

^1^Accession numbers are for predicted mRNA.

^2^[16].

^3^[14,18].

^4^[19].

^5^[41].

^6^[27].

^7^Active site residues including those in occluding loop [55].

*Predicted homologs according to lack of sequence conservation in active site residues.

Unlike *T. castaneum*, there was only one highly expressed cathepsin L in *T. molitor*, contig TmL13 (Table 2). Similarly, one cathepsin B contig, TmB33, had the most reads. These data may reflect the relative importance of cathepsin B and L in the two insects.

### Phylogeny of *T. castaneum* and *T. molitor* cysteine cathepsins

**Figure 1:**
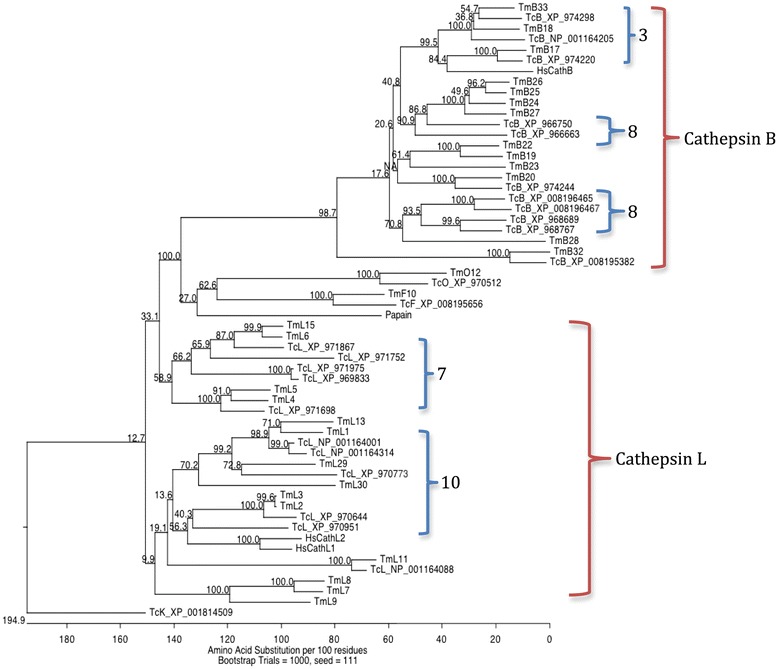
**Cladogram of B, L, O, and F cysteine cathepsins from** ***T. molitor*** **(TmL_, TmB_, Tm_O, Tm_F) and** ***T. castaneum*** **(TcL_, TcB_, Tc_O, Tc_F),** ***Carica papaya*** **(papain, AAB02650), and** ***Homo sapiens*** **cathepsin L (HsCathL1, NP_001903, HsCathL2, AAI10513) or B (HsCathB, AAH10240), with outgroup sequence cathepsin K (TcK_XP_001814509) from** ***T. castaneum*** **.** Clades corresponding to chromosomes in *T. castaneum* as well as cathepsin groups (B or L) are indicated.

The location of *T. castaneum* cysteine peptidase genes in chromosomes indicated that they were either single genes or clusters of tandem duplicated genes on chromosomes 3, 7, 8 and 10. Phylogeny supports this hypothesis, as overall genes within a cluster form one clade on a phylogenetic tree (Figure 1). Cathepsin L and B groups were separated by cathepsin O and F in the cladogram. Orthologs between *T. castaneum* and *T. molitor* were found in clades within ortholog pairs or ortholog groups, including the most highly expressed genes. However, some sequences lacked orthologs, which may be due to lack of sequence data or functional divergence and independent evolution of those peptidases. Homolog sequences (those lacking conserved residues in the active site) also clustered, suggesting that they diverged prior to the separation of the tenebrionid lineage.

### Comparison of cathepsin L and related peptidases in *T. molitor* and *T. castaneum*

Orthologous cathepsin L peptidase genes found on chromosome 10 in *T. castaneum* had the highest expression levels of all peptidase genes, NP_001164001 and NP_001164314 in *T. castaneum* and TmL13 in *Т. molitor* (Tables 1 and 2). These orthologs belong to gene expansion groups and in previous transcriptome and proteome studies were annotated as major digestive peptidases in *T. molitor* and *T. castaneum* [19,21,27,41]. *T. molitor* had only one highly expressed cathepsin L gene, but this clade also included an orthologous TmL1 peptidase, which had a low RPKM.

In *T. castaneum*, the two major cathepsin L peptidases were expressed significantly higher (92% of total transcripts) than the *T. molitor* major cathepsin L (about 62%) (Tables 1 and 3). Orthologous cathepsin L transcripts with a moderate level of expression belonged to another cluster on chromosome 7 and included TmL5 (*T. molitor*) and XP_971698 (*T. castaneum*), about 6% and 2% of the total cysteine peptidase expression, respectively. TmL5 was previously characterized as digestive cathepsin L (ppCAL2, AAR05023) by [19]. Another orthologous pair of moderately transcribed sequences was TmL11 and NP_001164088 (5% and 1%, respectively). All of the remaining cathepsin L transcripts, as well as orthologs for cathepsin F (TmF10 and XP_973607) and cathepsin O (TmO12 and XP_970512), had minor expression levels in the gut transcriptomes. Orthologs were found for all cathepsin L genes, except for three from *T. molitor* (TmL7, TmL8, and TmL9) and five from *T. castaneum*, three included in clusters (XP_970951, XP_971752, and XP_971975), and two single genes (XP_969833 and XP_001814509). The expression levels correlated to our previous biochemical data, demonstrating the greater significance of cysteine peptidases in the digestive process for *T. castaneum* than for *T. molitor* [14,17,18].

**Table 3:** **Comparison of putative cathepsin L orthologs in** ***T. molitor*** **and** ***T. castaneum*** **, and comparison of key residues to those in human cathepsin L1 (NP_001903) and L2 (AAI10513)**

	**Substrate binding site**						**Substrate binding site**				
	**Key residues** ^**1**^						**Key residues**				
	**S1**	**S2**					**S1**	**S2**			
	**23|65**	**67|68**	**133**	**157**	**205**		**23|65**	**67|68**	**133**	**157**	**205**
Cathepsin L1 human	GG	LM	A	M	A		GG	LM	A	M	A
Cathepsin L2 human	GG	FM	A	L	A		GG	FM	A	L	A
***Tenebrio molitor***						***Tribolium castaneum***					
TmL13	GG	WM	A	L	A	NP_001164001	GG	WM	A	L	A
TmL1	GG	YM	A	L	Q	NP_001164314	GG	WM	A	L	A
TmL2	GG	LM	A	L	E	XP_970644	GG	LM	A	L	Q
TmL3	GG	LM	A	L	E	XP_970951	AG	LM	A	V	Q
TmL29*	GG	LT	A	L	S	XP_970773*	GG	HA	T	L	S
TmL30*	GG	SI	A	L	D						
TmL4	GG	WM	A	F	K						
TmL5	GG	WM	A	F	V	XP_971698	GG	WM	A	F	K
TmL6	GG	WM	A	F	K	XP_971867	GG	WM	A	F	K
TmL15	GG	WM	A	F	Q	XP_971752	GG	YL	S	K	R
						XP_971975	GG	WM	A	L	H
						XP_969833	GG	WI	A	L	H
TmL11	GG	ED	G	L	T	NP_001164088	GG	ED	A	L	T
TmL7	MQ	LD	T	F	I						
TmL8	MQ	LD	T	F	R						
TmL9	LE	ME	I	Y	Y						
TmF10	GG	LM	A	L	P	XP_008195656	GG	LM	A	L	P
TmO12	GG	DV	A	L	E	XP_970512	GG	DI	A	L	E
						XP_001814509	GG	SL	S	V	Y

^1^Papain numbering.

*Predicted homolog.

An alignment of all cathepsin L predicted protein sequences from *T. castaneum* and *T. molitor* demonstrated sequence conservation of the active site residues QCHN in the majority of the sequences (Additional file 2: Figure S1). Three predicted inactive homologs, XP_970773, TmL29, and TmL30 had substitutions QC → ES and CA. All sequences had predictions of signal peptides except XP_970773 and XP_970951 and cathepsin O XP_970512. Single nucleotide polymorphisms (SNPs) were detected in XP_971698, NP_001164088, XP_970773, and NP_001164314, but all were in non-conserved regions (data not shown).

### Comparison of cathepsin B peptidases in *T. molitor* and *T. castaneum*

The expression of cathepsin B and B-like peptidase transcripts were notably lower than that of cathepsin L in both insects (Tables 1 and 2). The highest expression levels were from a gene located on the third chromosome of *T. castaneum*, XP_974298, and *T. molitor* ortholog TmB33 (3% and 11%, respectively; Table 4). Other orthologs constituted much less of the total peptidase transcriptome: TmB20 and XP_974244 (2% and 0.2%, respectively), TmB18 and NP_001164205 (both 1.0%). *T. castaneum* cathepsin B genes from the cluster on chromosome 3 and a single gene on chromosome 7 had orthologs in the *T. molitor* gut, but genes located in two clusters on chromosome 8 had no clear orthologous pairs in *T. molitor*. This may indicate that the common ancestor was a single cathepsin gene in chromosome 8, which duplicated independently in each insect. Overall, the proportion of the total expression of cathepsin B transcripts in the *T. molitor* gut was significantly higher than that in the gut of *T. castaneum*: 21% vs. 5%, respectively.

**Table 4:** **Comparison of putative cathepsin B orthologs in** ***T. molitor*** **and** ***T. castaneum*** **, and comparison of key residues to those in human cathepsin B (P07858)**

	**Substrate binding site**						**Substrate binding site**				
	**Key residues** ^**1**^						**Key residues**				
	**S1**	**S2**					**S1**	**S2**			
	**23|65**	**67|68**	**133**	**157**	**205**		**23|65**	**67|68**	**133**	**157**	**205**
Cathepsin B human	GG	YP	A	G	E		GG	YP	A	G	E
***Tenebrio molitor***						***Tribolium castaneum***					
TmB33	GG	WP	D	G	D	XP_974298	GG	WP	D	G	D
TmB18	GG	YP	S	G	D	NP_001164205	GG	MP	S	G	G
TmB17	GG	FP	A	G	E	XP_974220	GG	FP	A	G	S
TmB20	GG	YM	N	G	Y	XP_974244	GG	YM	S	G	N
TmB19	GG	YI	G	G	Y						
TmB22	GG	YM	G	G	N						
TmB23	GG	YV	T	G	Y						
TmB24	GG	AP	N	G	N	XP_966663	GG	YS	S	G	N
TmB25	GG	WP	S	G	N	XP_966750	GG	AP	H	G	Y
TmB27	GG	WM	A	F	Q						
TmB26	GG	SS	S	G	N						
						XP_008196467	GG	YO	Y	G	E
						XP_008196465	GG	YT	T	X	E
						XP_968767	GG	YS	G	G	S
						XP_968689*	SG	YT	A	G	S
TmB28*	SG	SS	I	S	H						
TmB32*	GG	YL	T	G	F	XP_008195382*	GG	YL	T	G	F

^1^Papain numbering.

*Predicted homolog.

QCHN active site residues were conserved in all except TmB32, XP_968689 and XP_967834, in which C → S (all) and H → T (XP_968689) were found; we consider these sequences inactive homologs (Additional file 3: Figure S2). The changes in the active site conserved region were identical for orthologs TmB32 and XP_967834 [QG(S → W)CGS(C → S)WA(F → I)], evidence of mutations in these genes prior to species divergence. TmB17, TmB18, and TmB33 and XP_974220, XP_974298 and NP_01164205 were classified as “typical” cathepsin B peptidases, containing two His residues in the occluding loop (marked by black frame), similar to human cathepsin B. All other cathepsin B-like peptidases form a novel cathepsin B-like group of peptidases with atypical shortened occluding loops lacking additional active site residues, including the homologs TmB32, XP_968689 and XP_967834. Transcripts of typical cathepsin B peptidases were exclusively mapped to chromosome 3, and this cluster, including also a pair of cathepsin B-like orthologs, accounted for the major expression of cathepsin B genes in the gut of both insects. *T. castaneum* cathepsin B transcript sequences with SNPs included XP_966750, XP_968689, XP_974220, XP_974298, and NP_001164205 (data not shown). There were five SNPs in the N-terminal region of XP_966750; most other SNPs were random and not found in conserved residues.

### Structural analysis of cathepsin B peptidases

As previously mentioned, cathepsin B sequences revealed major differences in the occluding loop. The occluding loop is typical only for three pairs of orthologous cathepsins (*T. molitor* B17, B18, B33 and *T. castaneum* XP_974220, NP_001164205, XP_974298, respectively) and consists of about 25 amino acid residues similar to human cathepsin B. The models of “typical” cathepsin B peptidases show a conserved structure in subsites S1 and S2 (Additional file 4: Figure S3A). Two histidines (His110, His111) of the occluding loop can function as an additional active center, which in mammals provides exopeptidase activity in acid conditions [50]. In the remaining 10 sequences in *T. molitor* and eight in *T. castaneum*, the occluding loop contains only about 15 residues and lacks the two histidines required for exopeptidase activity (Additional file 4: Figure S3B).

**Figure 2:**
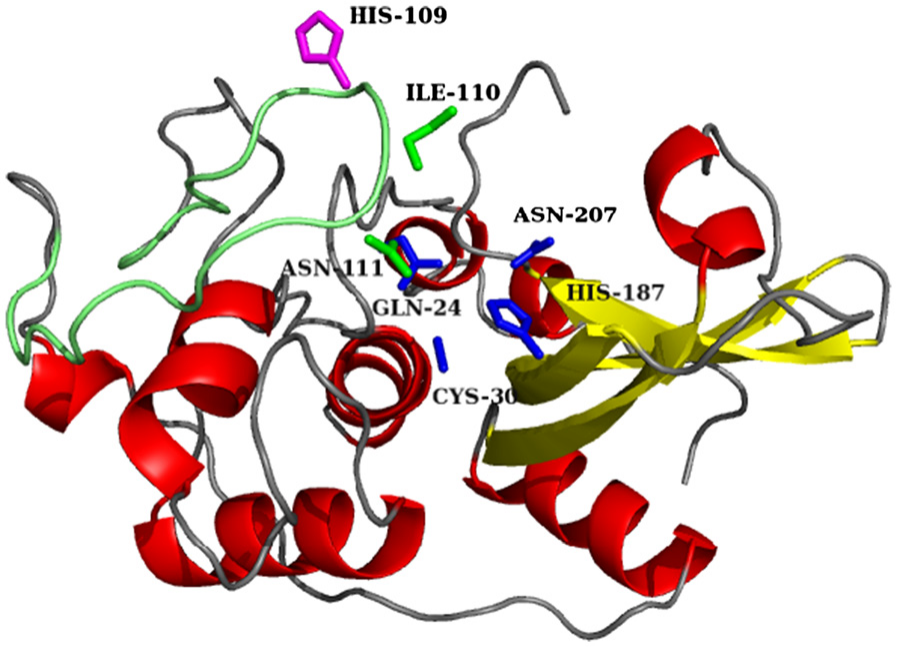
**Predicted structure of TmB19, an atypical B-like peptidase from** ***T. molitor*** **, obtained by 3D modeling.** Dark blue - active site (residues Gln-24, Cys-30, His-187, Asn-207); purple – His -109 and green Ile-110, Asn-111 are in the short occluding loop, which is marked light green.

3D modeling of cathepsin B-like sequences also demonstrated considerable changes in the structure of the “lid hinge” and the absence of the active center of the two histidines in the occluding loop in atypical cathepsin B-like peptidases (Figure 2). In a typical cathepsin B, the occluding loop reaches the active center and the histidine pair is in close proximity to the main active center (Additional file 4: Figure S3A). The shortened occluding loop in atypical cathepsin B-like peptidases does not reach the active center (Additional file 4: Figure S3B and Figure 2). This structure would imply a new group of cathepsins presumably lacking exopeptidase activity, not described previously for humans and mammals but only in insects. Combined with phylogenetic data, we suggest that this group originated from typical cathepsin B, but through evolutionary changes, the peptidase has lost the exopeptidase function.

For two *T. molitor* sequences (TmB19, TmB20), it was previously suggested that the site of AH in the occluding loop participates as an exopeptidase [16]. However, the 3D model did not predict this activity, because the relative distance of these residues was far from the active center core (Figure 2).

### Analysis of the substrate binding sites

We compared the 3D structures of complexes of a peptide-like inhibitor and model cysteine peptidases from the C1 family: papain and human mature cathepsins L1 (3OF8), mature cathepsin S (1NQC), and *Schistosoma mansoni* mature cathepsin B (3S3R), to identify amino acid residues in the S1 and S2 subsites that presumably play the most important role in the substrate specificity of C1 peptidases (data not shown). We found five amino acid residues whose radicals were located within 5 Å of the substrate-like inhibitor and thus could affect the substrate specificity of S2 subsite - 67, 68, 133, 157, 205, similar to another report [51]. We also identified two amino acid residues that presumably determine the substrate specificity of the S1 subsite - 23, 65. These amino acids are only a part of previously published list involved in the formation of the S1 subsites [52]. Our list is shorter because most of the proposed hydrogen bonds in the S1 subsite are formed between the substrate and the backbone atoms of the enzyme and are not affected by their radicals.

To determine residues in the binding subsites of *T. molitor* and *T. castaneum* peptidases, alignments were made of the predicted sequences of cathepsin L and B peptidases with human cathepsin L1 (P07711) [53] and L2 (O60911) [54], and B (P07858) [55], respectively. To further clarify and confirm the location of subsites in the predicted sequences, we performed a pairwise comparison of 3D models of cathepsin L and B from *T. molitor* with 3D structures of human mature cathepsin L2 (3KFQ, chain A; Renko and Turk, unpublished) and mature cathepsin B (3CBJ, chain A; Redzynia et al., unpublished), respectively. Cathepsin L-like sequences of both insects contained a significant amount of variability in the substrate binding sites, especially at the S2 subsite (Table 3, Additional file 3: Figure S2). S1 subsites contained conserved Gly23, Gly65 residues in most sequences, including inactive homologs TmL29, TmL30, and XP_970773. However, one group of *T. molitor* transcripts (TmL7, TmL8, TmL9) lacked homology in the S1 subsite (Table 3, Additional file 3: Figure S2). In the S2 subsite, with residues responsible for the specificity of cathepsin L, a spectrum of amino acid residues was found that differed in physico-chemical properties. For example, position 205, crucial in determining the specificity of cysteine peptidases from the C1 family, was occupied not only by small nonpolar amino acids (Ala) typical in mammalian cathepsins L, but also by large nonpolar like Ile, polar (Gln, Tyr, Thr) and charged (Lys, Arg, His, Glu) residues. Variations also were found in other residues of the S2 subsite.

Two sequences of cathepsin L peptidases in *T. castaneum* (NP_001164001, NP_001164314) and orthologs TmL13 and TmL1 in *T. molitor*, respectively, were closest to human cathepsin L in the S1 and S2 subsites (Table 3). *T. castaneum* genes from this set belong to one cluster located on chromosome 10, indicating that this cluster and the orthologus cluster in *T. molitor* are the closest to human cathepsin L, although other members of this cluster have differences in the 205 position, including A → E. Tenebrionid cathepsins closest to human cathepsin L have a high level of expression in the gut and include biochemically identified digestive peptidases [14,16-19,21].

*T. castaneum* cathepsins L from chromosome 7 (XP_971698, XP_971867, XP_971975, XP_969833) and a related cathepsin L from chromosome 3, and orthologous *T. molitor* cathepsins TmL4 and TmL6, have basic residues in the 205 position (Lys, His, Arg). Position 157 in this cluster was mostly occupied with a large aromatic Phe residue. Mammalian cathepsin L peptidases contain nonpolar Ala205 and Met157 (HsCath1) or Leu157 (HsCath2) residues in the S2 subsite, so tenebrionid cathepsin L from this cluster may have different substrate specificity. This cluster contains the transcripts with moderate expression levels (XP_971698, TmL4, TmL5). One group of specific *T. molitor* transcripts (TmL7, TmL8, TmL9) lacked homology in both S1 and S2 subsites and may have specific regulatory functions. Orthologous cathepsin O and F in both insects had similar substrate binding sites, except for acidic Asp 67 and Glu205 in both cathepsin O peptidases.

Most cathepsin B sequences, including inactive homologs (XP_008195352 and TmB32), contained typical Gly residues in the S1 subsite, except for TmB28 and inactive XP_968689, which contained Ser65 (Table 4, Additional file 3: Figure S2). Typical cathepsin B sequences with the complete occluding loop in *T. castaneum* (XP_974220, NP_001164205, XP_974298) and orthologs in *T. molitor* (TmB17, TmB18 and TmB33) contained negatively charged amino acids (Asp, Glu) at position 205 in S2 subsites, consistent with the Glu205 in mammalian cathepsin B (except in XP_974220 and NP_001164205, it was Ser). In most atypical cathepsin B-like sequences with a shortened occluding loop, located in two clusters on chromosome 8, position 205 was occupied by polar uncharged residues (Tyr and Asn in *T. molitor* and also Ser in *T. castaneum*). Therefore, affinity to substrates with Arg at position P2, characteristic for human cathepsin B, is different in predicted atypical cathepsin B-like peptidases of tenebrionids. TmB28 differed substantially from other B-like cathepsins, not only in the S1 subsite (Ser65), but also in the S2 subsite with a positively charged His in the 205 position. *T. castaneum* has two cathepsin B peptidases (previously characterized as pseudogenes XP_008196465 and XP_008196467 in the Tcas3 genome build) with atypical occluding loops containing Glu205, and these peptidases may have similar substrate specificity as mammalian cathepsins B.

### Molecular docking of substrates FRF and LRF to selected cysteine peptidases from *T. molitor*

To model the substrate specificity of *T. molitor* cysteine cathepsins, the tripeptides FRF and LRF were used as the substrates in docking experiments (Additional file 5: Figure S4). Docking of these substrates to the mature human cathepsin L1 enzyme (3OF8) [33] was performed as a reference. The free energy of FRF substrate binding to the active site of human cathepsin L was equal to -7.5 to - 7.7 kcal/mol (Table 5). There were two possible conformations of the radical of arginine in the P1 position of the substrate in the active center of human cathepsin L. In one conformation, the arginine amide group reacted with the oxygen of the primary chain and residues Cys63 and Asn64 (Additional file 5: Figure S4A), which was slightly less stable than the preferred conformation (-7.5 kcal/mol). In the preferred conformation (-7.7 kcal/mol), the substrate reacted with the oxygen atoms of the primary chain and the radical residue Asp158 (Additional file 5: Figure S4B). However, replacement of Asp158 to Phe158 in the cathepsin L 3D structure retained binding of the substrate, with a slight rise of the free energy of binding (-6.1 to - 7.1 kcal/mol). Replacing Gly23 and 65 of the S1 binding subsite to phenylalanine resulted in partial or complete loss of affinity for the substrate. For Gly23 to Phe23, access to the active site was restricted, while changing Gly65 to Phe65 allowed the substrate access to the active site, but the conformation required for catalysis was significantly hampered. Apparently, the significance of Gly 23 and 65 in substrate specificity is because it allows access to the active site without steric hindrance. The larger radicals physically impede access of the substrate to the active site.

**Table 5:** **The binding energy of peptide substrates in the models of the active site of cathepsins in** ***T. molitor*** **compared to that of human cathepsin L1 (3OF8), using the substrates FRF and LRF**

**Cathepsin**	**Free energy of binding (kcal/mol)**	
	**Substrate FRF**	**Substrate LRF**
Human cathepsin L1	−7.7	- 6.1
TmL2	−7.6	-
TmL5	−7.1	−3.3
TmL7	-	-
TmL8	-	-
TmL13	−6.9	−5.5
TmB18	−7.6	−3.9
TmB33	−8.7	−6.6

The exact characteristics of the substrate specificity of *T. molitor* cathepsins are not yet known experimentally, so we performed *in silico* studies of their specificity. For these experiments, we picked cathepsins with highest expression (TmL2, TmL5, TmL13, TmB18, TmB33) and cathepsins with changes in the S1 subsite (TmL7, TmL8, TmL9, TmB28). Mature enzyme models were constructed for all of the chosen peptidases. All models were evaluated by Ramachandran plots (Additional file 6: Figure S5). For peptidases TmL9 and TmB28, there were significant changes in the model structure (less than 90% of amino acids were in a favored region, or some of the amino acids forming the subsites were in outlier region) that corresponded to the major differences in the sequence of the peptidases. Therefore, these two peptidase sequences were not selected for further analysis.

As part of the model evaluation, we compared mature peptidase models (TmL5, TmL13) with crystal structures of corresponding mutant procathepsins 3QJ3 and 3QT4 [41], respectively (Additional file 7: Figure S6A,B). Both TmL15 and TmL13 models were close to the crystal structures: root mean square deviation was 0.199 and 0.173 angstroms, respectively, and differences in active site amino acid positions were not larger than 0.05 angstroms. The comparison of mature vs procathepsin L from *H. sapiens* is provided for reference (Additional file 7: Figure S6C).

Docking studies were conducted with peptide substrates FRF and LRF, differing in position P2, and the model of the active site of *T. molitor* sequences TmL2, TmL5, Tm7, TmL8, TmL13, TmB18, and TmB33. Sequences TmL2, TmL5 and TmL13 demonstrated FRF substrate affinity, and free binding energies of the substrate were similar to each other and with the binding energy of the substrate with human cathepsin L (Table 5). TmL13 had similar affinity to the substrate LRF as mammalian cathepsin L, and the affinity of TmL5 was significantly lower, and TmL2, containing Glu205 that is atypical for cathepsin L, had no affinity for this substrate. Nevertheless, the substrate specificity of this cathepsin group was similar to human cathepsin L. Cathepsins TmL7 and TmL8 predictably had no affinity to either of these substrates due to changes in glycines in the binding site of S1. Thus, these enzymes have different substrate specificity or no catalytic activity, as their sequences are radically different from those described for the model cysteine peptidases.

In substrate docking studies, typical *T. molitor* cathepsin B peptidases (TmB18, TmB33) were predicted to freely form an enzyme-substrate complex, with a preference for the substrate FRF with an aromatic amino acid at position P2 (Table 5). Interestingly, TmB33, which contains an additional negatively charged Asp133, showed maximal affinity to both substrates, even higher than mammalian cathepsin B.

## Discussion

In this study, using different algorithms to analyze RNA-Seq data, we were able to predict cysteine peptidase genes in sequences from the larval gut of *T. molitor* and *T. castaneum* that were similar to peptidases from the C1 papain family, calculate relative expression values, and analyze *in silico* the structure of predicted peptidases. The datasets of C1 cysteine peptidase transcripts in the guts of two insect larvae were similar, but not identical. They included transcripts from orthologous genes as well as those lacking close homologs, suggesting that they originated after the divergence of species. Alternatively, without a sequenced genome for *T. molitor*, our analyses are limited by the available transcriptome data; these sequence models will be improved by additional sequencing.

The data on C1 peptidase expression, together with biochemical [17] and proteomic [21] data, indicate that the most abundant C1 endopeptidases, cathepsin L, have a major role in protein digestion in *T. castaneum*. Their transcripts constitute 95% of the total expression of cysteine peptidase transcripts in the larval gut, while transcripts of exo/endopeptidases, cathepsin B, constitute only 5%. So, digestion in this insect was expanded to accommodate different substrates, as cathepsin L has broad substrate specificity. The primary digestive peptidases in *T. castaneum* are two cathepsin L peptidases (NP_001164001 and NP_001164314), encoded by neighboring genes on chromosome 10 (LOC659441 and LOC659502), which are highly expressed in the gut and are most similar to the mammalian cathepsin L in the structure of substrate binding site. The *T. molitor* larval gut contains only one highly expressed orthologous cathepsin L, TmL13, and the impact of cathepsin B transcripts as a percentage of total peptidase transcripts (21%) is higher than in *T. castaneum*. However, we did not find a correlation between the expression profile and the structure of the peptidase clusters. Therefore, the regulation of expression in tandem genes may be independent.

We propose that the structure of substrate binding S2 subsite, containing residues Trp67, Met68, Ala133, Leu157, Ala205, provides the most effective hydrolysis of proteins, because it is characteristic for *T. castaneum* and *T. molitor* major digestive peptidases and for human cathepsin L2, with the only substitution Leu67. The other eight cathepsin L peptidases in *T. castaneum* and 11 cathepsin L peptidases in *T. molitor* have substitutions in S2 subsite, and levels of their transcripts expression are much lower under normal dietary conditions. However, we know that the expression of tenebrionid peptidases can change in response to dietary inhibitors or toxins [22,27].

As C1 cysteine peptidases in tenebrionids perform the most important initial steps in protein digestion, we speculate that the 5-6-fold increased speed of larval development in *T. castaneum* compared to *T. molitor* may be, at least in part, due to the overwhelming role of cathepsin L in food digestion, and this can be one of the factors facilitating the distribution of these larvae around the world.

We predict that cathepsin L and B peptidases with moderate expression in the gut of tenebrionids, like XP_971698, NP_001164088 (both cathepsin L), XP_974244, XP_966750 (both cathepsin B) in *T. castaneum* and their orthologs or homologs in *T. molitor* (TmL5, TmL11, TmB20, TmB25, TmB26) may be lysosomal enzymes. These enzymes have increased diversity in the structure of substrate binding sites, suggesting the possibility to hydrolyze a wide variety of substrates. The remaining transcripts of cathepsin L, B, and also F and O with low level of expression in the larval guts of both insects most probably are involved in regulatory processes. Transcripts of cathepsin L and B with negligible level of expression, like TmL15, TmL8, TmB22 and their cathepsin L orthologs (homologs) mainly from chromosome 7, XP_971868, XP_971975, XP_971752, XP_969833, as well as cathepsin B from chromosome 8, XP_966663, may have specific regulatory roles or are not expressed in the gut and may be an artifact of sequence assembly or tissue contamination. Cathepsin K, which is found in the *T. castaneum* genome, is expressed primarily in the embryo (data not shown).

By examining the *in silico* primary and tertiary structures of the predicted peptidases, we were able to demonstrate that peptidases similar to cathepsin B can be divided in two groups: those with typical cathepsin B structures and containing occluding loops, and cathepsin B-like proteins with a short loop lacking two His residues, that are apparently unable to function as exopeptidases. Cathepsin B transcripts with the highest expression levels in both insects were orthologs and belong to the cluster of typical cathepsin B (NP_001164205, XP_974298, TmB18, TmB33). The structure of the substrate-binding site in cathepsins from this cluster (chromosome 3 in *T. castaneum*) was similar to mammalian cathepsin B, and the enzymes contained acidic residues in position 205 of the S2 subsite, which enables the hydrolysis of peptide bonds after basic amino acid residues in the substrate. Cathepsin B-like peptidases from another cluster (chromosome 8) with short occluding loops presumably will not accept basic residues in the P1 position due to the absence of acidic residues in position 205.

Docking characteristic peptide substrates FRF and LRF to the active center of *T. molitor* cathepsin L and B supported the analysis of the primary structures and 3D models of enzymes. Cathepsins TmB18, TmB33 and TmL13, with a typical binding site structure, formed complexes with these substrates with binding energy comparable to mammalian homologs. Enzymes with slightly altered S2 subsites, TmL5, TmB18 and TmL2, showed lower or no affinity to LRF, while enzymes with an altered S1 and S2 subsites, TmL7 and TmL8, did not bind these substrates in the proper position, and thus have entirely different specificity, or may be inactive. These data suggest that a Gly-Gly pair in the S1 subsite is crucial for substrate binding, and proteins with changes in this pair will lack typical substrate affinity.

## Conclusions

Three main groups of cysteine peptidases were identified in the gut of tenebrionid insects: cathepsin L, cathepsin B, and a new group of cathepsin B-like peptidases that lack an additional active site in the occluding loop. Using genomic, transcriptome, and microarray data from this and previous studies, we have identified 11 cathepsin L and 11 cathepsin B-like peptidases transcripts in the gut of *T. castaneum* larvae. Using transcriptome data acquired in this and previous studies, we found 14 complete predicted peptidase sequences similar to cathepsin L and related peptidases, and 13 cathepsin B and B-like peptidases transcripts in the *T. molitor* larval gut. In addition, there were sequences encoding cathepsin F and O in both insects, but expression levels were low, and cathepsin K was found only in the *T. castaneum* genome. The most highly expressed peptidases in both insects were orthologous cathepsin L peptidases, with an additional highly expressed cathepsin L in *T. castaneum*. The expression of cathepsin B peptidases was much lower than cathepsin L in both insects. Most cysteine cathepsin B and L peptidases had considerable variability in the substrate binding sites, consistent with the hypothesis that evolution in cysteine cathepsins has enabled the insects to survive diets high in proline and glutamine, as well as seed inhibitors of peptidases. A new group of atypical cathepsin B-like peptidases was described with shortened occluding loops. These data provide unique perspectives of protein digestion in these tenebrionids and the most comprehensive data for coleopteran peptidases to date.

## Additional files

Additional file 1: Table S1. Some developmental and life history characteristics of the tenebrionids *T. molitor* and *T. castaneum.*
Additional file 2: Figure S1. Alignment of predicted amino acid sequences of cysteine cathepsin L, O, F, and K from *T. molitor* and *T. castaneum*, *Homo sapiens* cathepsin L (HsCathL1, NP_001903, HsCathL2, AAI10513), and papain (*Carica papaya*, AAB02650). Conserved residues are marked with a black asterisk and black box; residues in the S1 subsite are in turquoise box; residues in the S2 subsite are in green box. Homologs are marked with red asterisk and sequences lacking a signal peptide are underlined in red on first page; the signal peptide recognition site is marked by a red vertical line. Additional file 3: Figure S2. Alignment of amino acid sequences of cysteine cathepsin B from *T. molitor* and *T. castaneum*, *Homo sapiens* cathepsin B (HsCathB, AAH10240), and papain (*Carica papaya*, AAB02650). Conserved residues are marked with a black asterisk and black box; residues in the S1 subsite are in turquoise box; residues in the S2 subsite are in green box. The occluding loop region is bracketed in black lines. Homologs are marked with red asterisk and sequences lacking a signal peptide are underlined in red on first page; the signal peptide recognition site is marked by a red vertical line. Additional file 4: Figure S3 Models of three-dimensional structures of representatives of two groups of cathepsins B in *T. molitor* larvae*.* A, typical cathepsin B TmB33; B, atypical cathepsin B-like TmB22. Dark blue - active site (residues Gln-19, Cys-25, His-159, Asn-175); light blue - S1 substrate binding site (residues 23, 66, 158); purple - S2 substrate binding site (residues 67, 68, 133, 157, 160, 205); the occluding loop is colored green. Additional file 5: Figure S4. Models of two possible conformations (A and B) of Arg in the P1 position of the substrate FRF in the active site of human cathepsin L (3OF8) [33]. Additional file 6: Figure S5. Ramachandran plots of the analysis of active site and S2 subsite of models of *T. molitor* predicted mature cysteine cathepsins (TmL2, TmL4, TmL5, TmL7, TmL8, TmL9, TmL11, TmL13, TmB18, TmB19, TmB20, TmB22, TmB28, and TmB33). Additional file 7: Figure S6. The models of mature peptidases from *T. molitor* (A, TmL5; B, TmL13) were compared with crystal structures of corresponding mutant procathepsins 3QJ3 and 3QT4 [41], respectively, using PDBeFold [39]. C, the comparison of mature (3OF8) vs procathepsin L (1CS8) from *H. sapiens*.
