# Supplementary material for: Functional analysis of C1 family cysteine peptidases in the larval gut of Тenebrio molitor and Tribolium castaneum
Source: BMC Genomics. 2015 Feb 14;16(1):75. doi: 10.1186/s12864-015-1306-x (PMC4336737; doi:10.1186/s12864-015-1306-x)
Supplement: Additional file 1: Table S1. — Some developmental and life history characteristics of the tenebrionids T. molitor and T. castaneum. [file 12864_2015_1306_MOESM1_ESM.docx]

**Additional file 1**

**Table S1.** Some developmental and life history characteristics of the tenebrionids *T. molitor* and *T. castaneum.*

| **Characteristic** | ***T. molitor*** | ***T. castaneum*** |
| --- | --- | --- |
| Size | Larvae up to 30 mm | Larvae up to 6 mm |
| Larval period | 120-629 d (up to 21 instars) | 22-100 d |
| Life cycle | 16-88 w (adults live 2-3 mo) | 4-12 w (adults live 1-3 y) |
| Economic | Reared as animal feed; can be host to human parasite | One of the most serious coleopteran storage pests worldwide |
| Biochemical | Many studies (mostly digestion) | Few studies |
| Genetic | Few studies | Genetic model for coleopterans |
