# Supplementary material for: Functional analysis of C1 family cysteine peptidases in the larval gut of Тenebrio molitor and Tribolium castaneum
Source: BMC Genomics. 2015 Feb 14;16(1):75. doi: 10.1186/s12864-015-1306-x (PMC4336737; doi:10.1186/s12864-015-1306-x)
Supplement: Additional file 2: Figure S1. — Alignment of predicted amino acid sequences of cysteine cathepsin L, O, F, and K from T. molitor and T. castaneum, Homo sapiens cathepsin L (HsCathL1, NP_001903, HsCathL2, AAI10513), and papain (Carica papaya, AAB02650). Conserved residues are marked with a black asterisk and black box; residues in the S1 subsite are in turquoise box; residues in the S2 subsite are in green box. Homologs are marked with red asterisk and sequences lacking a signal peptide are underlined in red on first page; the signal peptide recognition site is marked by a red vertical line. [file 12864_2015_1306_MOESM2_ESM.pdf]

```
                   ------MKTLIVLALCL---------------------------------------------------------------
                            10        20        30        40        50        60        70        80
*TmL30             ------MKCFILLTATF--------------------------------------------------------------- 12
*TmL29             ------MKILWILSLVL--------------------------------------------------------------- 12
*TcL_XP_970773     -------------------------------------------------------------------------------- 1
TmL13              ------MKTLLVLCLAF--------------------------------------------------------------- 12
TcL_NP_001164001   ------MKTFIVLALAV--------------------------------------------------------------- 12
TcL_NP_001164314   ------MKTFIVLALAV--------------------------------------------------------------- 12
TmL1               ------MKTFVVLSLAI--------------------------------------------------------------- 12
HsCathL2           -----MNLSLVLAAFCL--------------------------------------------------------------- 13
HsCathL1           -----MNPTLILAAFCL--------------------------------------------------------------- 13
TmL3               ------MKFLIFLAICV--------------------------------------------------------------- 12
TmL2               ------MKFLIFLAICV--------------------------------------------------------------- 12
TcL_XP_970644      ------MKFLVFVALCV--------------------------------------------------------------- 12
TcL_XP_970951      -----MKTGLVFVATVV--------------------------------------------------------------- 13
TcL_XP_971752      ------MKVLTLLVVL---------------------------------------------------------------- 11
TmL15              ------MKILVVLLLSL--------------------------------------------------------------- 12
TmL6               ------MKILVVLLVSL--------------------------------------------------------------- 12
TcL_XP_971867      ------MRTLVVLLLVL--------------------------------------------------------------- 12
TcL_XP_971975      ------MHTLAVLLLCA--------------------------------------------------------------- 12
TcL_XP_969833      ------MHTLAVLLLCA--------------------------------------------------------------- 12
TmL5               ------MKVFIILAIAI--------------------------------------------------------------- 12
TmL4               ------MKVLVILAITI--------------------------------------------------------------- 12
TcL_XP_971698      ------MKVFIILALAI--------------------------------------------------------------- 12
TmL9               -----MKPQLLLFFVFL--------------------------------------------------------------- 13
TmL8               ------MKWFLLLIVSI--------------------------------------------------------------- 12
TmL7               ------MKLLLLLFVSI--------------------------------------------------------------- 12
TmL11              --MGTVVKSVIFSCFLTLVYGDCAVTAPVWSNTHTVKGILYIPYAEIEEPFYAWYDGPSKRSRIDYYGDMVKTYQLSTSG 78
TcL_NP_001164088   --MRMLFKSVIFSCFLALVYGDCSNKAPAWSNTYTVKGILYIPYAEIEEPFYAWYDGQSKRSRIDYYGDMVKTYQLSTEG 78
Papain             MAMIPSISKLLFVAICLFVYM----------------------------------------------------------- 22
TmF10              ------MALLRCLFICLCCSLFVNSEVNQENGANAYDSHDAEIAAVLNHLNSTPIKVYNYKQATLINAHTKADITELTLD 74
TcF_XP_008195656   ------MATLWYMFVCFCWWSLVNSDVLQENLSNKSESHSSEISAVLNHLNSSPFKVYNYNKATLLNAQTKADIIELILD 74
TmO12              ---MAPWTTYLQVFFCV--------------------------------------------------------------- 15
TcO_XP_970512      ---MASWSTYLQAIFYI--------------------------------------------------------------- 15
TcK_XP_001814509   ------MKTILIIAL----------------------------------------------------------------- 10
```

```
                  --------------------------------------------------------------------------------
                           90        100       110       120       130       140       150       160
TmL30             -------------------------------------------------------------------------------- 12
TmL29             -------------------------------------------------------------------------------- 12
TcL_XP_970773     -------------------------------------------------------------------------------- 1
TmL13             -------------------------------------------------------------------------------- 12
TcL_NP_001164001  -------------------------------------------------------------------------------- 12
TcL_NP_001164314  -------------------------------------------------------------------------------- 12
TmL1              -------------------------------------------------------------------------------- 12
HsCathL2          -------------------------------------------------------------------------------- 13
HsCathL1          -------------------------------------------------------------------------------- 13
TmL3              -------------------------------------------------------------------------------- 12
TmL2              -------------------------------------------------------------------------------- 12
TcL_XP_970644     -------------------------------------------------------------------------------- 12
TcL_XP_970951     -------------------------------------------------------------------------------- 13
TcL_XP_971752     -------------------------------------------------------------------------------- 11
TmL15             -------------------------------------------------------------------------------- 12
TmL6              -------------------------------------------------------------------------------- 12
TcL_XP_971867     -------------------------------------------------------------------------------- 12
TcL_XP_971975     -------------------------------------------------------------------------------- 12
TcL_XP_969833     -------------------------------------------------------------------------------- 12
TmL5              -------------------------------------------------------------------------------- 12
TmL4              -------------------------------------------------------------------------------- 12
TcL_XP_971698     -------------------------------------------------------------------------------- 12
TmL9              -------------------------------------------------------------------------------- 13
TmL8              -------------------------------------------------------------------------------- 12
TmL7              -------------------------------------------------------------------------------- 12
TmL11             PYGTSLKIAPVTTETE---------------------------------------------------------------- 95
TcL_NP_001164088  PYGSSLKIAPVTTETE---------------------------------------------------------------- 95
Papain            -------------------------------------------------------------------------------- 22
TmF10             VSVDCINNYPDVPCTETSLTCNAFVRSTAEEGSQVLNDVNCKSKPEIIVEEPVYDPTTSPND-DANETKSIALSNEAVSS 153
TcF_XP_008195656  IDVDCINKYPNVPCGEKVVTCHAFVRNTPEEGSKVLTDVNCKPKPEIIVDEPVYDSPTNPTEVNTNEPTPIVLSNEAVSS 154
TmO12             -------------------------------------------------------------------------------- 15
TcO_XP_970512     -------------------------------------------------------------------------------- 15
TcK_XP_001814509  -------------------------------------------------------------------------------- 10
```

```
                     -------------YXSXAXL------EXLVEE------------------------------------------------
                              170       180       190       200       210       220       230       240
TmL30                -------------VVGTLSS------SEELHP------------------------------------------------ 25
TmL29                -------------AATATPL--------SIQK------------------------------------------------ 23
TcL_XP_970773        ----------------MASP------QKLIND------------------------------------------------ 11
TmL13                -------------AATLALP------KSPFQE------------------------------------------------ 25
TcL_NP_001164001     -------------VATLAVP------QSPVHA------------------------------------------------ 25
TcL_NP_001164314     -------------VATLAVP------QSPVHA------------------------------------------------ 25
TmL1                 -------------VTTLALP------KSPLNE------------------------------------------------ 25
HsCathL2             -------------GIASAVP----KFDQNLDT------------------------------------------------ 28
HsCathL1             -------------GIASATL----TFDHSLEA------------------------------------------------ 28
TmL3                 -------------AGSQAVS-----FFDLVQE------------------------------------------------ 26
TmL2                 -------------AGSQAVS-----FFDLVQE------------------------------------------------ 26
TcL_XP_970644        -------------VGSQAVS-----FFDLVQE------------------------------------------------ 26
TcL_XP_970951        -------------AFAKSQLSIGVTLENLLQE------------------------------------------------ 32
TcL_XP_971752        -------------HTSDAFS---------LNK------------------------------------------------ 21
TmL15                -------------YACQASL-----PESVVPD------------------------------------------------ 26
TmL6                 -------------YTCQAGL-----PESLISE------------------------------------------------ 26
TcL_XP_971867        -------------YTSDASS---------LNE------------------------------------------------ 22
TcL_XP_971975        -------------YSSQALL-----PENLVEE------------------------------------------------ 26
TcL_XP_969833        -------------YSSQALL-----PENLVEE------------------------------------------------ 26
TmL5                 -------------YGASAAL-----PSTFVAE------------------------------------------------ 26
TmL4                 -------------YGTSATL-----SSEFVAE------------------------------------------------ 26
TcL_XP_971698        -------------YGTSASL-----SKDFVEE------------------------------------------------ 26
TmL9                 -------------CNCSAGK-----------N------------------------------------------------ 21
TmL8                 -------------SAVFASP------------------------------------------------------------ 19
TmL7                 -------------NVVFASQ------------------------------------------------------------ 19
TmL11                -------------TNARTCLQVNGTSEYTIEP------------------------------------------------ 114
TcL_NP_001164088     -------------TNSRTCLQVNGTEENTIEP------------------------------------------------ 114
Papain               -------------GLSFGDFSIVGYSQNDLTS------------------------------------------------ 41
TmF10                EVGDHFVAVRRESYPCLGCPIDLNPNAEGVDELVDIGLGHIESEREQKQSLVKVLKLQQQVVSGVKYILVVEVAPTICAK 233
TcF_XP_008195656     EVGEHFVAVRKDSVPCLGCPFDLNTNAEGVDELIDVALEHIQTERAKKHALVKVLRLQQQVVTGVKYILTAEFAPTLCEK 234
TmO12                -------------ALLFFMIPIKIDGPDEVEL------------------------------------------------ 34
TcO_XP_970512        -------------ALLFFVIPIRIKGPDQAES------------------------------------------------ 34
TcK_XP_001814509     -------------ATVLAKLAHFHENGASLDE------------------------------------------------ 29
```

```
                   --------------------------------------------------------------------------------
                            250       260       270       280       290       300       310       320
TmL30              -------------------------------------------------------------------------------- 25
TmL29              -------------------------------------------------------------------------------- 23
TcL_XP_970773      -------------------------------------------------------------------------------- 11
TmL13              -------------------------------------------------------------------------------- 25
TcL_NP_001164001   -------------------------------------------------------------------------------- 25
TcL_NP_001164314   -------------------------------------------------------------------------------- 25
TmL1               -------------------------------------------------------------------------------- 25
HsCathL2           -------------------------------------------------------------------------------- 28
HsCathL1           -------------------------------------------------------------------------------- 28
TmL3               -------------------------------------------------------------------------------- 26
TmL2               -------------------------------------------------------------------------------- 26
TcL_XP_970644      -------------------------------------------------------------------------------- 26
TcL_XP_970951      -------------------------------------------------------------------------------- 32
TcL_XP_971752      -------------------------------------------------------------------------------- 21
TmL15              -------------------------------------------------------------------------------- 26
TmL6               -------------------------------------------------------------------------------- 26
TcL_XP_971867      -------------------------------------------------------------------------------- 22
TcL_XP_971975      -------------------------------------------------------------------------------- 26
TcL_XP_969833      -------------------------------------------------------------------------------- 26
TmL5               -------------------------------------------------------------------------------- 26
TmL4               -------------------------------------------------------------------------------- 26
TcL_XP_971698      -------------------------------------------------------------------------------- 26
TmL9               -------------------------------------------------------------------------------- 21
TmL8               -------------------------------------------------------------------------------- 19
TmL7               -------------------------------------------------------------------------------- 19
TmL11              -------------------------------------------------------------------------------- 114
TcL_NP_001164088   -------------------------------------------------------------------------------- 114
Papain             -------------------------------------------------------------------------------- 41
TmF10              DSNLGAESCPRDSTVDSSVCKITYLHKPWITKAKHVIGNNCTVSQEFVKSDGRKMEMSNEIDGTGAKDVKPPTVVGPNFD 313
TcF_XP_008195656   SANLDASSCPRDTNAETTICEITYLHKPWISKAKHVIKNNCTVSQEYVRPDIRKSDISNEIDVSGKRT---KLIDSPNFD 311
TmO12              -------------------------------------------------------------------------------- 34
TcO_XP_970512      -------------------------------------------------------------------------------- 34
TcK_XP_001814509   -------------------------------------------------------------------------------- 29
```

```
                 --------------------------------------------------------------------------------
                        330       340       350       360       370       380       390       400
TmL30            -------------------------------------------------------------------------------- 25
TmL29            -------------------------------------------------------------------------------- 23
TcL_XP_970773    -------------------------------------------------------------------------------- 11
TmL13            -------------------------------------------------------------------------------- 25
TcL_NP_001164001 -------------------------------------------------------------------------------- 25
TcL_NP_001164314 -------------------------------------------------------------------------------- 25
TmL1             -------------------------------------------------------------------------------- 25
HsCathL2         -------------------------------------------------------------------------------- 28
HsCathL1         -------------------------------------------------------------------------------- 28
TmL3             -------------------------------------------------------------------------------- 26
TmL2             -------------------------------------------------------------------------------- 26
TcL_XP_970644    -------------------------------------------------------------------------------- 26
TcL_XP_970951    -------------------------------------------------------------------------------- 32
TcL_XP_971752    -------------------------------------------------------------------------------- 21
TmL15            -------------------------------------------------------------------------------- 26
TmL6             -------------------------------------------------------------------------------- 26
TcL_XP_971867    -------------------------------------------------------------------------------- 22
TcL_XP_971975    -------------------------------------------------------------------------------- 26
TcL_XP_969833    -------------------------------------------------------------------------------- 26
TmL5             -------------------------------------------------------------------------------- 26
TmL4             -------------------------------------------------------------------------------- 26
TcL_XP_971698    -------------------------------------------------------------------------------- 26
TmL9             -------------------------------------------------------------------------------- 21
TmL8             -------------------------------------------------------------------------------- 19
TmL7             -------------------------------------------------------------------------------- 19
TmL11            -------------------------------------------------------------------------------- 114
TcL_NP_001164088 -------------------------------------------------------------------------------- 114
Papain           -------------------------------------------------------------------------------- 41
TmF10            RMAETGALEEMTPSRLHDLESMIVVENDEPVQQNKKGQVDVGKRIDEVVVPSTVESQIQLESQPSGEVAVEAESTGNPLL 393
TcF_XP_008195656 KMAENGAIDEMTPSRLADLESQIIVDVEP----------------EIGAPSTAESHPQVESKLEFDL------------- 363
TmO12            -------------------------------------------------------------------------------- 34
TcO_XP_970512    -------------------------------------------------------------------------------- 34
TcK_XP_001814509 -------------------------------------------------------------------------------- 29
```

```
                  --------------------------------------------------------------------------------
                           410       420       430       440       450       460       470       480
TmL30             -------------------------------------------------------------------------------- 25
TmL29             -------------------------------------------------------------------------------- 23
TcL_XP_970773     -------------------------------------------------------------------------------- 11
TmL13             -------------------------------------------------------------------------------- 25
TcL_NP_001164001  -------------------------------------------------------------------------------- 25
TcL_NP_001164314  -------------------------------------------------------------------------------- 25
TmL1              -------------------------------------------------------------------------------- 25
HsCathL2          -------------------------------------------------------------------------------- 28
HsCathL1          -------------------------------------------------------------------------------- 28
TmL3              -------------------------------------------------------------------------------- 26
TmL2              -------------------------------------------------------------------------------- 26
TcL_XP_970644     -------------------------------------------------------------------------------- 26
TcL_XP_970951     -------------------------------------------------------------------------------- 32
TcL_XP_971752     -------------------------------------------------------------------------------- 21
TmL15             -------------------------------------------------------------------------------- 26
TmL6              -------------------------------------------------------------------------------- 26
TcL_XP_971867     -------------------------------------------------------------------------------- 22
TcL_XP_971975     -------------------------------------------------------------------------------- 26
TcL_XP_969833     -------------------------------------------------------------------------------- 26
TmL5              -------------------------------------------------------------------------------- 26
TmL4              -------------------------------------------------------------------------------- 26
TcL_XP_971698     -------------------------------------------------------------------------------- 26
TmL9              -------------------------------------------------------------------------------- 21
TmL8              -------------------------------------------------------------------------------- 19
TmL7              -------------------------------------------------------------------------------- 19
TmL11             -------------------------------------------------------------------------------- 114
TcL_NP_001164088  -------------------------------------------------------------------------------- 114
Papain            -------------------------------------------------------------------------------- 41
TmF10             ADTEQVMNDFAAPELSTVT--PESEEQATTLEPTPETTPKSELSQSKSDSSSDESKDSSEESDSKSRVKRDNKSDCSSSS 471
TcF_XP_008195656  ---------FQTPESLTVSEDPAFTAGVTVEEPAP-------------------------TEEHVSRLKRDESS------ 403
TmO12             -------------------------------------------------------------------------------- 34
TcO_XP_970512     -------------------------------------------------------------------------------- 34
TcK_XP_001814509  -------------------------------------------------------------------------------- 29
```

```
                   --------------------------------------------------------------------------------
                            490       500       510       520       530       540       550       560
TmL30              -------------------------------------------------------------------------------- 25
TmL29              -------------------------------------------------------------------------------- 23
TcL_XP_970773      -------------------------------------------------------------------------------- 11
TmL13              -------------------------------------------------------------------------------- 25
TcL_NP_001164001   -------------------------------------------------------------------------------- 25
TcL_NP_001164314   -------------------------------------------------------------------------------- 25
TmL1               -------------------------------------------------------------------------------- 25
HsCathL2           -------------------------------------------------------------------------------- 28
HsCathL1           -------------------------------------------------------------------------------- 28
TmL3               -------------------------------------------------------------------------------- 26
TmL2               -------------------------------------------------------------------------------- 26
TcL_XP_970644      -------------------------------------------------------------------------------- 26
TcL_XP_970951      -------------------------------------------------------------------------------- 32
TcL_XP_971752      -------------------------------------------------------------------------------- 21
TmL15              -------------------------------------------------------------------------------- 26
TmL6               -------------------------------------------------------------------------------- 26
TcL_XP_971867      -------------------------------------------------------------------------------- 22
TcL_XP_971975      -------------------------------------------------------------------------------- 26
TcL_XP_969833      -------------------------------------------------------------------------------- 26
TmL5               -------------------------------------------------------------------------------- 26
TmL4               -------------------------------------------------------------------------------- 26
TcL_XP_971698      -------------------------------------------------------------------------------- 26
TmL9               -------------------------------------------------------------------------------- 21
TmL8               -------------------------------------------------------------------------------- 19
TmL7               -------------------------------------------------------------------------------- 19
TmL11              -------------------------------------------------------------------------------- 114
TcL_NP_001164088   -------------------------------------------------------------------------------- 114
Papain             -------------------------------------------------------------------------------- 41
TmF10              SSSSSSSSEEDDCDEKKHKEEKKKKASEEKKHHKKDEKDSDSSSSSSSSSSSSSSSEEDDDKDKNKHRSERAIRQLEKIS 551
TcF_XP_008195656   -------------------EETKKDSS------------SESVETAANQTKATKTSSSEENSSEEVLRPKRAIRQLEKIS 451
TmO12              -------------------------------------------------------------------------------- 34
TcO_XP_970512      -------------------------------------------------------------------------------- 34
TcK_XP_001814509   -------------------------------------------------------------------------------- 29
```

```
                    --------------------------------------------------------------------------------
                             570       580       590       600       610       620       630       640
TmL30               -------------------------------------------------------------------------------- 25
TmL29               -------------------------------------------------------------------------------- 23
TcL_XP_970773       -------------------------------------------------------------------------------- 11
TmL13               -------------------------------------------------------------------------------- 25
TcL_NP_001164001    -------------------------------------------------------------------------------- 25
TcL_NP_001164314    -------------------------------------------------------------------------------- 25
TmL1                -------------------------------------------------------------------------------- 25
HsCathL2            -------------------------------------------------------------------------------- 28
HsCathL1            -------------------------------------------------------------------------------- 28
TmL3                -------------------------------------------------------------------------------- 26
TmL2                -------------------------------------------------------------------------------- 26
TcL_XP_970644       -------------------------------------------------------------------------------- 26
TcL_XP_970951       -------------------------------------------------------------------------------- 32
TcL_XP_971752       -------------------------------------------------------------------------------- 21
TmL15               -------------------------------------------------------------------------------- 26
TmL6                -------------------------------------------------------------------------------- 26
TcL_XP_971867       -------------------------------------------------------------------------------- 22
TcL_XP_971975       -------------------------------------------------------------------------------- 26
TcL_XP_969833       -------------------------------------------------------------------------------- 26
TmL5                -------------------------------------------------------------------------------- 26
TmL4                -------------------------------------------------------------------------------- 26
TcL_XP_971698       -------------------------------------------------------------------------------- 26
TmL9                -------------------------------------------------------------------------------- 21
TmL8                -------------------------------------------------------------------------------- 19
TmL7                -------------------------------------------------------------------------------- 19
TmL11               -------------------------------------------------------------------------------- 114
TcL_NP_001164088    -------------------------------------------------------------------------------- 114
Papain              -------------------------------------------------------------------------------- 41
TmF10               PDEKGVVKNLADFAASALDNIDDDNHKRIVLQILGAKKMVGDDGVYYHLIMRMGISRCLEDKPTDPYENCREKLFENYTK 631
TcF_XP_008195656    PDEKGLVKNLADFAANALDSIDDDNNKRIILQILGAKKMVGDDGVYYHIIMRMGVSRCLEDSPINPYENCKDKLFENYTK 531
TmO12               -------------------------------------------------------------------------------- 34
TcO_XP_970512       -------------------------------------------------------------------------------- 34
TcK_XP_001814509    -------------------------------------------------------------------------------- 29
```

```
                    --------------------------------------------------------------------------------
                             650       660       670       680       690       700       710       720
TmL30               -------------------------------------------------------------------------------- 25
TmL29               -------------------------------------------------------------------------------- 23
TcL_XP_970773       -------------------------------------------------------------------------------- 11
TmL13               -------------------------------------------------------------------------------- 25
TcL_NP_001164001    -------------------------------------------------------------------------------- 25
TcL_NP_001164314    -------------------------------------------------------------------------------- 25
TmL1                -------------------------------------------------------------------------------- 25
HsCathL2            -------------------------------------------------------------------------------- 28
HsCathL1            -------------------------------------------------------------------------------- 28
TmL3                -------------------------------------------------------------------------------- 26
TmL2                -------------------------------------------------------------------------------- 26
TcL_XP_970644       -------------------------------------------------------------------------------- 26
TcL_XP_970951       -------------------------------------------------------------------------------- 32
TcL_XP_971752       -------------------------------------------------------------------------------- 21
TmL15               -------------------------------------------------------------------------------- 26
TmL6                -------------------------------------------------------------------------------- 26
TcL_XP_971867       -------------------------------------------------------------------------------- 22
TcL_XP_971975       -------------------------------------------------------------------------------- 26
TcL_XP_969833       -------------------------------------------------------------------------------- 26
TmL5                -------------------------------------------------------------------------------- 26
TmL4                -------------------------------------------------------------------------------- 26
TcL_XP_971698       -------------------------------------------------------------------------------- 26
TmL9                -------------------------------------------------------------------------------- 21
TmL8                -------------------------------------------------------------------------------- 19
TmL7                -------------------------------------------------------------------------------- 19
TmL11               -------------------------------------------------------------------------------- 114
TcL_NP_001164088    -------------------------------------------------------------------------------- 114
Papain              -------------------------------------------------------------------------------- 41
TmF10               ICKVQVYVNDDFGSPKVVKSQCQNIKRDKNDSNRTNYSRFRRES------------------------------------ 676
TcF_XP_008195656    ICKVQVYVNDDFGSKKVVKSQCQNIKRDKNDNNRTNYSRYKRDGLGAPNRIDKNSEKIRQFVKEGINGFNANYNSKNNKV 611
TmO12               -------------------------------------------------------------------------------- 34
TcO_XP_970512       -------------------------------------------------------------------------------- 34
TcK_XP_001814509    -------------------------------------------------------------------------------- 29
```

```
                  --------------------------------------------------------------------------------
                           730       740       750       760       770       780       790       800
TmL30             -------------------------------------------------------------------------------- 25
TmL29             -------------------------------------------------------------------------------- 23
TcL_XP_970773     -------------------------------------------------------------------------------- 11
TmL13             -------------------------------------------------------------------------------- 25
TcL_NP_001164001  -------------------------------------------------------------------------------- 25
TcL_NP_001164314  -------------------------------------------------------------------------------- 25
TmL1              -------------------------------------------------------------------------------- 25
HsCathL2          -------------------------------------------------------------------------------- 28
HsCathL1          -------------------------------------------------------------------------------- 28
TmL3              -------------------------------------------------------------------------------- 26
TmL2              -------------------------------------------------------------------------------- 26
TcL_XP_970644     -------------------------------------------------------------------------------- 26
TcL_XP_970951     -------------------------------------------------------------------------------- 32
TcL_XP_971752     -------------------------------------------------------------------------------- 21
TmL15             -------------------------------------------------------------------------------- 26
TmL6              -------------------------------------------------------------------------------- 26
TcL_XP_971867     -------------------------------------------------------------------------------- 22
TcL_XP_971975     -------------------------------------------------------------------------------- 26
TcL_XP_969833     -------------------------------------------------------------------------------- 26
TmL5              -------------------------------------------------------------------------------- 26
TmL4              -------------------------------------------------------------------------------- 26
TcL_XP_971698     -------------------------------------------------------------------------------- 26
TmL9              -------------------------------------------------------------------------------- 21
TmL8              -------------------------------------------------------------------------------- 19
TmL7              -------------------------------------------------------------------------------- 19
TmL11             -------------------------------------------------------------------------------- 114
TcL_NP_001164088  -------------------------------------------------------------------------------- 114
Papain            -------------------------------------------------------------------------------- 41
TmF10             -------------------------------------------------------------------------------- 676
TcF_XP_008195656  KPVEVVSATTQVVAGTLYKITTKISESDCSKNDNKDLDDCNILEGASPKTCELEVWEKLWENFRQFTIKCEGDEQSIKFQ 691
TmO12             -------------------------------------------------------------------------------- 34
TcO_XP_970512     -------------------------------------------------------------------------------- 34
TcK_XP_001814509  -------------------------------------------------------------------------------- 29
```

```
                    --------------------------------------------------------------------------------
                              810       820       830       840       850       860       870       880
TmL30               -------------------------------------------------------------------------------- 25
TmL29               -------------------------------------------------------------------------------- 23
TcL_XP_970773       -------------------------------------------------------------------------------- 11
TmL13               -------------------------------------------------------------------------------- 25
TcL_NP_001164001    -------------------------------------------------------------------------------- 25
TcL_NP_001164314    -------------------------------------------------------------------------------- 25
TmL1                -------------------------------------------------------------------------------- 25
HsCathL2            -------------------------------------------------------------------------------- 28
HsCathL1            -------------------------------------------------------------------------------- 28
TmL3                -------------------------------------------------------------------------------- 26
TmL2                -------------------------------------------------------------------------------- 26
TcL_XP_970644       -------------------------------------------------------------------------------- 26
TcL_XP_970951       -------------------------------------------------------------------------------- 32
TcL_XP_971752       -------------------------------------------------------------------------------- 21
TmL15               -------------------------------------------------------------------------------- 26
TmL6                -------------------------------------------------------------------------------- 26
TcL_XP_971867       -------------------------------------------------------------------------------- 22
TcL_XP_971975       -------------------------------------------------------------------------------- 26
TcL_XP_969833       -------------------------------------------------------------------------------- 26
TmL5                -------------------------------------------------------------------------------- 26
TmL4                -------------------------------------------------------------------------------- 26
TcL_XP_971698       -------------------------------------------------------------------------------- 26
TmL9                -------------------------------------------------------------------------------- 21
TmL8                -------------------------------------------------------------------------------- 19
TmL7                -------------------------------------------------------------------------------- 19
TmL11               -------------------------------------------------------------------------------- 114
TcL_NP_001164088    -------------------------------------------------------------------------------- 114
Papain              -------------------------------------------------------------------------------- 41
TmF10               -------------------------------------------------------------------------------- 676
TcF_XP_008195656    TSPKNRAKRQAPGGESPVTKDNEYVVKYLEAALNQLDSESPHENKFKVHEFISATSQTVSGHIYRIKTKVVLSDCKKTAS 771
TmO12               -------------------------------------------------------------------------------- 34
TcO_XP_970512       -------------------------------------------------------------------------------- 34
TcK_XP_001814509    -------------------------------------------------------------------------------- 29
```

```
                  --------------------------------------------------------------------------------
                         890       900       910       920       930       940       950       960
TmL30             -------------------------------------------------------------------------------- 25
TmL29             -------------------------------------------------------------------------------- 23
TcL_XP_970773     -------------------------------------------------------------------------------- 11
TmL13             -------------------------------------------------------------------------------- 25
TcL_NP_001164001  -------------------------------------------------------------------------------- 25
TcL_NP_001164314  -------------------------------------------------------------------------------- 25
TmL1              -------------------------------------------------------------------------------- 25
HsCathL2          -------------------------------------------------------------------------------- 28
HsCathL1          -------------------------------------------------------------------------------- 28
TmL3              -------------------------------------------------------------------------------- 26
TmL2              -------------------------------------------------------------------------------- 26
TcL_XP_970644     -------------------------------------------------------------------------------- 26
TcL_XP_970951     -------------------------------------------------------------------------------- 32
TcL_XP_971752     -------------------------------------------------------------------------------- 21
TmL15             -------------------------------------------------------------------------------- 26
TmL6              -------------------------------------------------------------------------------- 26
TcL_XP_971867     -------------------------------------------------------------------------------- 22
TcL_XP_971975     -------------------------------------------------------------------------------- 26
TcL_XP_969833     -------------------------------------------------------------------------------- 26
TmL5              -------------------------------------------------------------------------------- 26
TmL4              -------------------------------------------------------------------------------- 26
TcL_XP_971698     -------------------------------------------------------------------------------- 26
TmL9              -------------------------------------------------------------------------------- 21
TmL8              -------------------------------------------------------------------------------- 19
TmL7              -------------------------------------------------------------------------------- 19
TmL11             -------------------------------------------------------------------------------- 114
TcL_NP_001164088  -------------------------------------------------------------------------------- 114
Papain            -------------------------------------------------------------------------------- 41
TmF10             ---------------------------------------------TLVGGANPTDKNSAKVKELINDGLTRLN------- 704
TcF_XP_008195656  TERGQCGTLKDAKPKTCKFEVFEQTWVPNSRRIKTDCDKERRKRKTLDGGITEVDKDDDEVKTFVREGLLNLNTHLTTSN 851
TmO12             -------------------------------------------------------------------------------- 34
TcO_XP_970512     -------------------------------------------------------------------------------- 34
TcK_XP_001814509  -------------------------------------------------------------------------------- 29
```

```
                  --------------------------------------------------------------------------------
                           970       980       990      1000      1010      1020      1030      1040
TmL30             -------------------------------------------------------------------------------- 25
TmL29             -------------------------------------------------------------------------------- 23
TcL_XP_970773     -------------------------------------------------------------------------------- 11
TmL13             -------------------------------------------------------------------------------- 25
TcL_NP_001164001  -------------------------------------------------------------------------------- 25
TcL_NP_001164314  -------------------------------------------------------------------------------- 25
TmL1              -------------------------------------------------------------------------------- 25
HsCathL2          -------------------------------------------------------------------------------- 28
HsCathL1          -------------------------------------------------------------------------------- 28
TmL3              -------------------------------------------------------------------------------- 26
TmL2              -------------------------------------------------------------------------------- 26
TcL_XP_970644     -------------------------------------------------------------------------------- 26
TcL_XP_970951     -------------------------------------------------------------------------------- 32
TcL_XP_971752     -------------------------------------------------------------------------------- 21
TmL15             -------------------------------------------------------------------------------- 26
TmL6              -------------------------------------------------------------------------------- 26
TcL_XP_971867     -------------------------------------------------------------------------------- 22
TcL_XP_971975     -------------------------------------------------------------------------------- 26
TcL_XP_969833     -------------------------------------------------------------------------------- 26
TmL5              -------------------------------------------------------------------------------- 26
TmL4              -------------------------------------------------------------------------------- 26
TcL_XP_971698     -------------------------------------------------------------------------------- 26
TmL9              -------------------------------------------------------------------------------- 21
TmL8              -------------------------------------------------------------------------------- 19
TmL7              -------------------------------------------------------------------------------- 19
TmL11             -------------------------------------------------------------------------------- 114
TcL_NP_001164088  -------------------------------------------------------------------------------- 114
Papain            -------------------------------------------------------------------------------- 41
TmF10             -------------------------------------------------------------------------------- 704
TcF_XP_008195656  KVKPVEVVSASVQVVAGSLHRIKVKISESDCSRNDQKDFEQCNVLEGASPKLCEMEVWDKPWEDFRRYTIKCEGENKKHQ 931
TmO12             -------------------------------------------------------------------------------- 34
TcO_XP_970512     -------------------------------------------------------------------------------- 34
TcK_XP_001814509  -------------------------------------------------------------------------------- 29
```

```
                 --------------------------------------------------------------------------------
                        1050      1060      1070      1080      1090      1100      1110      1120
TmL30            -------------------------------------------------------------------------------- 25
TmL29            -------------------------------------------------------------------------------- 23
TcL_XP_970773    -------------------------------------------------------------------------------- 11
TmL13            -------------------------------------------------------------------------------- 25
TcL_NP_001164001 -------------------------------------------------------------------------------- 25
TcL_NP_001164314 -------------------------------------------------------------------------------- 25
TmL1             -------------------------------------------------------------------------------- 25
HsCathL2         -------------------------------------------------------------------------------- 28
HsCathL1         -------------------------------------------------------------------------------- 28
TmL3             -------------------------------------------------------------------------------- 26
TmL2             -------------------------------------------------------------------------------- 26
TcL_XP_970644    -------------------------------------------------------------------------------- 26
TcL_XP_970951    -------------------------------------------------------------------------------- 32
TcL_XP_971752    -------------------------------------------------------------------------------- 21
TmL15            -------------------------------------------------------------------------------- 26
TmL6             -------------------------------------------------------------------------------- 26
TcL_XP_971867    -------------------------------------------------------------------------------- 22
TcL_XP_971975    -------------------------------------------------------------------------------- 26
TcL_XP_969833    -------------------------------------------------------------------------------- 26
TmL5             -------------------------------------------------------------------------------- 26
TmL4             -------------------------------------------------------------------------------- 26
TcL_XP_971698    -------------------------------------------------------------------------------- 26
TmL9             -------------------------------------------------------------------------------- 21
TmL8             -------------------------------------------------------------------------------- 19
TmL7             -------------------------------------------------------------------------------- 19
TmL11            -------------------------------------------------------------------------------- 114
TcL_NP_001164088 -------------------------------------------------------------------------------- 114
Papain           -------------------------------------------------------------------------------- 41
TmF10            -------------------------------------------------------------------------------- 704
TcF_XP_008195656 FTLNPNKRTKRQVPRGESPVAKDNEYVIKYLEAALNQLDAESEHENKFKVHEFISATSQIVSGHIYRINAKVILSDCKKT 1011
TmO12            -------------------------------------------------------------------------------- 34
TcO_XP_970512    -------------------------------------------------------------------------------- 34
TcK_XP_001814509 -------------------------------------------------------------------------------- 29
```

```
                   --------------------------------------------------------------------------------
                            1130      1140      1150      1160      1170      1180      1190      1200
TmL30              -------------------------------------------------------------------------------- 25
TmL29              -------------------------------------------------------------------------------- 23
TcL_XP_970773      -------------------------------------------------------------------------------- 11
TmL13              -------------------------------------------------------------------------------- 25
TcL_NP_001164001   -------------------------------------------------------------------------------- 25
TcL_NP_001164314   -------------------------------------------------------------------------------- 25
TmL1               -------------------------------------------------------------------------------- 25
HsCathL2           -------------------------------------------------------------------------------- 28
HsCathL1           -------------------------------------------------------------------------------- 28
TmL3               -------------------------------------------------------------------------------- 26
TmL2               -------------------------------------------------------------------------------- 26
TcL_XP_970644      -------------------------------------------------------------------------------- 26
TcL_XP_970951      -------------------------------------------------------------------------------- 32
TcL_XP_971752      -------------------------------------------------------------------------------- 21
TmL15              -------------------------------------------------------------------------------- 26
TmL6               -------------------------------------------------------------------------------- 26
TcL_XP_971867      -------------------------------------------------------------------------------- 22
TcL_XP_971975      -------------------------------------------------------------------------------- 26
TcL_XP_969833      -------------------------------------------------------------------------------- 26
TmL5               -------------------------------------------------------------------------------- 26
TmL4               -------------------------------------------------------------------------------- 26
TcL_XP_971698      -------------------------------------------------------------------------------- 26
TmL9               -------------------------------------------------------------------------------- 21
TmL8               -------------------------------------------------------------------------------- 19
TmL7               -------------------------------------------------------------------------------- 19
TmL11              -------QTILPDLTNFECTGEETISGILTEKWALTQTIGQKVNKYTMWLHYKDDPNNVGTKIAIPVRYEMNGYNSLLGS 186
TcL_NP_001164088   -------QSILPDLTGFECTGEETISGVLTEKWALSQTIGQKVNKYAMWLYYKDDPDNTGVKIAVPVKYEMRGFNSLLGS 186
Papain             -------------------------------------------------------------------------------- 41
TmF10              --AERG-------------------------------------------------------------------------- 708
TcF_XP_008195656   VSTERGQCGTLKDAKPKTCKFEVFEQLWVPNSRQIKTDCHKERRKRQTLPGGIKEVDKNNDEVKTFVHEGLLNLNTHLTT 1091
TmO12              -------------------------------------------------------------------------------- 34
TcO_XP_970512      -------------------------------------------------------------------------------- 34
TcK_XP_001814509   -------------------------------------------------------------------------------- 29
```

```
                             -------------------------------------------------------------Q-WENFKL-----------
                                      1210      1220      1230      1240      1250      1260      1270      1280
TmL30                        -------------------------------------------------------------K-WKNFKK----------- 32
TmL29                        ---------------------------------------------------------------WXEFKL----------- 29
TcL_XP_970773                -------------------------------------------------------------QNWSQFKL----------- 19
TmL13                        -------------------------------------------------------------Q-WSQFKL----------- 32
TcL_NP_001164001             -------------------------------------------------------------K-WAEFKL----------- 32
TcL_NP_001164314             -------------------------------------------------------------K-WAEFKL----------- 32
TmL1                         -------------------------------------------------------------T-WVQFKL----------- 32
HsCathL2                     -------------------------------------------------------------K-WYQWKA----------- 35
HsCathL1                     -------------------------------------------------------------Q-WTKWKA----------- 35
TmL3                         -------------------------------------------------------------Q-WGAFKM----------- 33
TmL2                         -------------------------------------------------------------Q-WGAFKM----------- 33
TcL_XP_970644                -------------------------------------------------------------Q-WGAFKV----------- 33
TcL_XP_970951                -------------------------------------------------------------E-WMAFKL----------- 39
TcL_XP_971752                -------------------------------------------------------------E-WENFKR----------- 28
TmL15                        -------------------------------------------------------------E-WSNFKN----------- 33
TmL6                         -------------------------------------------------------------Q-WTNFKN----------- 33
TcL_XP_971867                -------------------------------------------------------------K-WENFKQ----------- 29
TcL_XP_971975                -------------------------------------------------------------E-WNKFKA----------- 33
TcL_XP_969833                -------------------------------------------------------------E-WNKFKA----------- 33
TmL5                         -------------------------------------------------------------K-WENFKT----------- 33
TmL4                         -------------------------------------------------------------K-WENFKT----------- 33
TcL_XP_971698                -------------------------------------------------------------K-WESFKK----------- 33
TmL9                         -------------------------------------------------------------E-WEIFKA----------- 28
TmL8                         -------------------------------------------------------------D-WDSFKE----------- 26
TmL7                         -------------------------------------------------------------D-WNAFKE----------- 26
TmL11                        HYDHYYLDYDSFDSDPIPDDIFKLDSNMTCSAFPGPGDKHIYTFNPMAEFVHPAKTSHVDYE-FNKFTR----------- 255
TcL_NP_001164088             HYDHYYLDYDSFSSDPIPDDIFKFDSNMKCSAFPGPGDKHIYTFNPMAEFIRPEKSGHVDFE-FGKFTR----------- 255
Papain                       --------------------------------------------------------TERLIQLFESWML----------- 54
TmF10                        GQKHKSVEIVSASLQVVAGTLYRIKVKISPTDCSSNDRKKLDDCNVKGGASPKNCEIKVWEKPWENFKQFTITCDGDEKV 787
TcF_XP_008195656             SNKVKPVEVVSASVQVVAGSLHRIKVKISESDCSRNDQKDFEQCNVLEGASPKLCEMEVWDKPWEDFRRYTIKCEGENKK 1171
TmO12                        -------------------------------------------------------------Q-FLDYLE----------- 41
TcO_XP_970512                -------------------------------------------------------------Q-FQEYLK----------- 41
TcK_XP_001814509             -------------------------------------------------------------Q-WEKFKQ----------- 36
```

```
                      --------------------------------------------------------------------------------
                               1290      1300      1310      1320      1330      1340      1350      1360
TmL30                 -------------------------------------------------------------------------------- 32
TmL29                 -------------------------------------------------------------------------------- 29
TcL_XP_970773         -------------------------------------------------------------------------------- 19
TmL13                 -------------------------------------------------------------------------------- 32
TcL_NP_001164001      -------------------------------------------------------------------------------- 32
TcL_NP_001164314      -------------------------------------------------------------------------------- 32
TmL1                  -------------------------------------------------------------------------------- 32
HsCathL2              -------------------------------------------------------------------------------- 35
HsCathL1              -------------------------------------------------------------------------------- 35
TmL3                  -------------------------------------------------------------------------------- 33
TmL2                  -------------------------------------------------------------------------------- 33
TcL_XP_970644         -------------------------------------------------------------------------------- 33
TcL_XP_970951         -------------------------------------------------------------------------------- 39
TcL_XP_971752         -------------------------------------------------------------------------------- 28
TmL15                 -------------------------------------------------------------------------------- 33
TmL6                  -------------------------------------------------------------------------------- 33
TcL_XP_971867         -------------------------------------------------------------------------------- 29
TcL_XP_971975         -------------------------------------------------------------------------------- 33
TcL_XP_969833         -------------------------------------------------------------------------------- 33
TmL5                  -------------------------------------------------------------------------------- 33
TmL4                  -------------------------------------------------------------------------------- 33
TcL_XP_971698         -------------------------------------------------------------------------------- 33
TmL9                  -------------------------------------------------------------------------------- 28
TmL8                  -------------------------------------------------------------------------------- 26
TmL7                  -------------------------------------------------------------------------------- 26
TmL11                 -------------------------------------------------------------------------------- 255
TcL_NP_001164088      -------------------------------------------------------------------------------- 255
Papain                -------------------------------------------------------------------------------- 54
TmF10                 HKFETTSSRR-KRGVPGGEFEVSKDDEFVIKYLDGGLSKLDGESSHPNKLKVHEVVSATQQVVAGDIYRIKAKIVLSDCK 866
TcF_XP_008195656      HQFTLNPNKRTKRQVRGGESPVAKDNEYVIKYLEAALNQLDAESEHENKFKVHEFISATSQIVSGHIYRIKAKVILSDCK 1251
TmO12                 -------------------------------------------------------------------------------- 41
TcO_XP_970512         -------------------------------------------------------------------------------- 41
TcK_XP_001814509      -------------------------------------------------------------------------------- 36
```

```
                     --------------------------------------------------------------------------------
                              1370      1380      1390      1400      1410      1420      1430      1440
TmL30                --------------------------------------------------------------------------------  32
TmL29                --------------------------------------------------------------------------------  29
TcL_XP_970773        --------------------------------------------------------------------------------  19
TmL13                --------------------------------------------------------------------------------  32
TcL_NP_001164001     --------------------------------------------------------------------------------  32
TcL_NP_001164314     --------------------------------------------------------------------------------  32
TmL1                 --------------------------------------------------------------------------------  32
HsCathL2             --------------------------------------------------------------------------------  35
HsCathL1             --------------------------------------------------------------------------------  35
TmL3                 --------------------------------------------------------------------------------  33
TmL2                 --------------------------------------------------------------------------------  33
TcL_XP_970644        --------------------------------------------------------------------------------  33
TcL_XP_970951        --------------------------------------------------------------------------------  39
TcL_XP_971752        --------------------------------------------------------------------------------  28
TmL15                --------------------------------------------------------------------------------  33
TmL6                 --------------------------------------------------------------------------------  33
TcL_XP_971867        --------------------------------------------------------------------------------  29
TcL_XP_971975        --------------------------------------------------------------------------------  33
TcL_XP_969833        --------------------------------------------------------------------------------  33
TmL5                 --------------------------------------------------------------------------------  33
TmL4                 --------------------------------------------------------------------------------  33
TcL_XP_971698        --------------------------------------------------------------------------------  33
TmL9                 --------------------------------------------------------------------------------  28
TmL8                 --------------------------------------------------------------------------------  26
TmL7                 --------------------------------------------------------------------------------  26
TmL11                --------------------------------------------------------------------------------  255
TcL_NP_001164088     --------------------------------------------------------------------------------  255
Papain               --------------------------------------------------------------------------------  54
TmF10                KSVSTERAKCGKLSGAEPKTCDFVVFEQVWVPNSRVTNVTCD---------DEVHSFRNKRETL----------------  922
TcF_XP_008195656     KSISTERGQCGTLKDAKPKTCKFEVFEQLWVPNSRQIKTECDGKRVKRQRSEKPHSVRSKRQSIPGGATEIDKKSDKVKQ  1331
TmO12                --------------------------------------------------------------------------------  41
TcO_XP_970512        --------------------------------------------------------------------------------  41
TcK_XP_001814509     --------------------------------------------------------------------------------  36
```

```
                  --------------------------------------------------------------------------------
                           1450      1460      1470      1480      1490      1500      1510      1520
TmL30             -------------------------------------------------------------------------------- 32
TmL29             -------------------------------------------------------------------------------- 29
TcL_XP_970773     -------------------------------------------------------------------------------- 19
TmL13             -------------------------------------------------------------------------------- 32
TcL_NP_001164001  -------------------------------------------------------------------------------- 32
TcL_NP_001164314  -------------------------------------------------------------------------------- 32
TmL1              -------------------------------------------------------------------------------- 32
HsCathL2          -------------------------------------------------------------------------------- 35
HsCathL1          -------------------------------------------------------------------------------- 35
TmL3              -------------------------------------------------------------------------------- 33
TmL2              -------------------------------------------------------------------------------- 33
TcL_XP_970644     -------------------------------------------------------------------------------- 33
TcL_XP_970951     -------------------------------------------------------------------------------- 39
TcL_XP_971752     -------------------------------------------------------------------------------- 28
TmL15             -------------------------------------------------------------------------------- 33
TmL6              -------------------------------------------------------------------------------- 33
TcL_XP_971867     -------------------------------------------------------------------------------- 29
TcL_XP_971975     -------------------------------------------------------------------------------- 33
TcL_XP_969833     -------------------------------------------------------------------------------- 33
TmL5              -------------------------------------------------------------------------------- 33
TmL4              -------------------------------------------------------------------------------- 33
TcL_XP_971698     -------------------------------------------------------------------------------- 33
TmL9              -------------------------------------------------------------------------------- 28
TmL8              -------------------------------------------------------------------------------- 26
TmL7              -------------------------------------------------------------------------------- 26
TmL11             -------------------------------------------------------------------------------- 255
TcL_NP_001164088  -------------------------------------------------------------------------------- 255
Papain            -------------------------------------------------------------------------------- 54
TmF10             -------------------------------------------------------------------------------- 922
TcF_XP_008195656  YVRESLTHLNTQLTSSNKVKPVEVLSATSQVVAGTIHRIKVKISESDCSKDDEKDFDECNIREGASPKICEVKVWDKPWQ 1411
TmO12             -------------------------------------------------------------------------------- 41
TcO_XP_970512     -------------------------------------------------------------------------------- 41
TcK_XP_001814509  -------------------------------------------------------------------------------- 36
```

```
                    ----------------------------------------------------THNKQYSNPKEENFRKXIFQKNLEKIEE
                             1530      1540      1550      1560      1570      1580      1590      1600
TmL30               ----------------------------------------------------LHQKSY-DRVEELKRIQIFEANLKKIEE 58
TmL29               ----------------------------------------------------AHQKQYSSPGEEAKRLKIFTENLAKIEA 56
TcL_XP_970773       ----------------------------------------------------THKKEYSTKTEEMKRLAIFTENLSKIDA 46
TmL13               ----------------------------------------------------THKKSYSSPIEEIRRQLIFKDNVAKIAE 59
TcL_NP_001164001    ----------------------------------------------------THKKQYSSPIEELRRKAIFQDNLVKIEE 59
TcL_NP_001164314    ----------------------------------------------------THKKQYSSPIEELKRMAIFQDNLVKIEE 59
TmL1                ----------------------------------------------------THQKEYSSPLEEIHRQAIFNENLVKIEE 59
HsCathL2            ----------------------------------------------------THRRLY-GANEEGWRRAVWEKNMKMIEL 61
HsCathL1            ----------------------------------------------------MHNRLY-GMNEEGWRRAVWEKNMKMIEL 61
TmL3                ----------------------------------------------------THNKQYQSETEERFRMKIFMENSHTVAK 60
TmL2                ----------------------------------------------------THNKQYQSDTEERFRMKIFMENSHTVAK 60
TcL_XP_970644       ----------------------------------------------------THKKQYESETEERFRMKIFMENAHKVAK 60
TcL_XP_970951       ----------------------------------------------------TYNKSYASPEEENFRREIFIENRHKIAR 66
TcL_XP_971752       ----------------------------------------------------KYERRYPNLEEENFRRAIFEKTFQEIKH 55
TmL15               ----------------------------------------------------LHRRRFMNPVEEVFRERIFEKKLQEVEN 60
TmL6                ----------------------------------------------------SHRRRFMNPVEEVFRKRIFQKKLQDVEN 60
TcL_XP_971867       ----------------------------------------------------KHGRNFLFSKEEFFRKSLFQKKLQEIED 56
TcL_XP_971975       ----------------------------------------------------MHARAFFDPLEETFRKSLFTKNLEIVEE 60
TcL_XP_969833       ----------------------------------------------------MHARAFFDPLEETFRKSLFTKNLEIVEE 60
TmL5                ----------------------------------------------------TYARSYVNAKEETFRKQIFQKKLETFEE 60
TmL4                ----------------------------------------------------SYSRSYVNAKEEAFRKQIFQKKLERFEE 60
TcL_XP_971698       ----------------------------------------------------THEKSYLNAKEEAFRKQIFQKKLERIEA 60
TmL9                ----------------------------------------------------THHKAYQNESEDNFRKSIFLSNLELIES 55
TmL8                ----------------------------------------------------KHHKIYKNEKEENYRKQIFLKNLKVIEE 53
TmL7                ----------------------------------------------------KYHKVYKNSKEENYRKQIFLKNLKVIEE 53
TmL11               ----------------------------------------------------KHRKQYKNETDHALRKEIFRQNVRFIHS 282
TcL_NP_001164088    ----------------------------------------------------KHGKNYQNKTETLMRKDIFRQNVRFIHS 282
Papain              ----------------------------------------------------KHNKIYKNIDEKIYRFEIFKDNLKYIDE 81
TmF10               --------------------------------GFLGRDDETLHFDMFKSFIKRHNKQY-TKKEFKYRFKVFRDNMYRVRV 968
TcF_XP_008195656    NFRQYNITCEGEDHHYTFETKPGKTKRSAELDNLLGCDDREYHLSLFTDFLKKYNKKY-HKKEYKYRFNVFVQNLMQIRV 1490
TmO12               ----------------------------------------------------KFHKTYDGDMYQS-KLQAFKKSLDNINA 67
TcO_XP_970512       ----------------------------------------------------RFNKTYDDPSVYQNRLHAFKQSLQTIET 68
TcK_XP_001814509    ----------------------------------------------------SYNKTYYGYLDTS-RKLAWQSNLEKIKK 62
```

```
                   HNEKYRQGLVTYTLGVNKFADMTPEEFLA-----YLNGLNXPK--PKPXEX---SPLFLPRAN---LPASVDWRDKGAVT
                           1610      1620      1630      1640      1650      1660      1670      1680
TmL30              HNKLFDKNEVSYKMAVNQFGDLSQEEFLD-----YLKTYNNKGGAKTTKSVKLQPHLNAE------VPDEIDWRENGAVT 127
TmL29              HNVRYEKGEVTYSKAMNKFGDWTAEEFLA-----FVNSTRFQK--PRLTKA--PPKLHLNRRL---AEDEVDWRSKGAVT 124
TcL_XP_970773      HNTKYRNGEVTYFKAMNKFGDLTTDEFLA-----FVNRNKLTK--REKNEK--HTKLNTTKIE---YETQVDWRANGLVS 114
TmL13              HNAKFEKGEVTYSKAMNQFGDMSKEEFLA-----YVNRGKAQK--PKHPEN-LRMPYVSSKKP---LAASVDWRSN-AVS 127
TcL_NP_001164001   HNAKFAKGEVTYTKAVNQFADMTADEFMA-----YVNRGLATK--PKMNEK-LRIPFVKSGKP---AAAEVDWRSK-AVT 127
TcL_NP_001164314   HNAKFAKGEVTYSKAVNQFADMTADEFMA-----YVNRGLATK--PKKNEK-LRLPFVQSDKP---AAAEVDWRNS-AVS 127
TmL1               HNAKYARGEVTYTKAMNRFGDMTKEEFLA-----FVNRGKAQK--PKISEK-LRIPYVESKKP---RADSVDWRLD-AVS 127
HsCathL2           HNGEYSQGKHGFTMAMNAFGDMTNEEFRQ-----MMGCFRNQK--FRKGKV-FREPLFLD------LPKSVDWRKKGYVT 127
HsCathL1           HNQEYREGKHSFTMAMNAFGDMTSEEFRQ-----VMNGFQNRK--PRKGKV-FQEPLFYE------APRSVDWREKGYVT 127
TmL3               HNKLYAQGLVSFKLGINKYADMLHHEFVQ-----VLNGFNRTKSGLRSGES-DDSVTFLPPANVQ-LPGQIDWRDKGAVT 133
TmL2               HNKLYAQGLVSFKLGINKYADMLHHEFVQ-----VLNGFNRTKSGLRSGES-DDSVTFLPPANVQ-LPGQIDWRDKGAVT 133
TcL_XP_970644      HNKLYAQGLVSFKLGVNKYSDMLNHEFVH-----TLNGYNRSKTPLRSGEL-DESITFIPPANVE-LPKQIDWRKLGAVT 133
TcL_XP_970951      FNQEYGRGQWSFVQQLNNFADMLHHEFHR-----TLNGFNRTLSARVGIPQ---SSTFIPSANVI-FPDYVDWREVGAVT 137
TcL_XP_971752      HNERYRKGLETYYLRINDLSDYTDEEMSC-----------CSEKAPKPSITILPNVSTSSRQN---LPKMVDWRLRGVVT 121
TmL15              HNERYRQGLETYELGVNQFSDYTEEELTP-----YTHGLLLPSEAPEPLISIDANSSLVARAR---LPASFDWRTRGVVT 132
TmL6               HNERYRQGLETYELGVNQFSDYTEEELLP-----YTHGLRLPSETPDPLISIDANSSLVARAT---LPASLDWRSRGVVT 132
TcL_XP_971867      HNERYRKGLETYEMGINKFSDYTDDELFS-----YTHGLQLPSELPEPIIK-ISPNATLSLSRAG-LPSSFDWRSRGVIT 129
TcL_XP_971975      HNERFRNGSETYEMGVNKFSDFTDEELAN------LTGLQVPPEFEQPLNE-TEDPLLLSLGRG--IPASLDWRQRGGVT 131
TcL_XP_969833      HNERFRNGSETYEMGVNKFSDFTDEELSN------LTGLQVPLEFEQPLNE-TEDPLLPSLGRG--ISASLDWRQRGGVT 131
TmL5               HNEKYRQGLVSYTLGVNLFTDMTPEEMKA-----YTHGLIMPADLHKNGIP-IKTREDLGLNASVRYPASFDWRDQGMVS 134
TmL4               HNEKFRQGLVTYTLGINKFADMTEEEIKP-----YTHGLVQPAQLPKPLFE-IKSREDLGLDAVGEHPASFDWRDQGMVT 134
TcL_XP_971698      HNERFNKGLETYTMGINMFTDMTPEEMRP-----YTHGLIEPAVVPKPLVE-IKSRADLGLNHSVQYPASFDWRDKGMVT 134
TmL9               HNRKYDQGLVSYSLQINHFADHTVEDLTR---------GSRPSTKPHKKPS-----KATPRAE---VPDSIDWRDYGVIT 118
TmL8               HNEKYAQGLSTYSVGVNNLADLTHEEIVF-----WSQGSSRRNLSVGSSPI----PKFIPRDD---VPESIDWRDYGVVT 121
TmL7               HNKKYTQGLSTYTIGVNKFADLTHEEIYS-----SSKSSTRREVTTVRSSP---TPKFIPRDD---VPESIDWRDYGVVT 122
TmL11              VNRQNK----GFTLTVNHLADKNPAELKALRGRTYSGGYNGGAPFPYKNIQ---------KEA---LPDQWDWRLYGAVT 346
TcL_NP_001164088   MNRQNR----GFTLTVNHLADKTPTELKALRGRTYSGGYNGGAPFPYENIN---------KED---LPDQWDWRLLGAVT 346
Papain             TNKKNN----SYWLGLNVFADMSNDEFKE-----KYTGSIAGNYTTTELSY--EEVLNDGDVN---IPEYVDWRQKGAVT 147
TmF10              LNA-YEQGTAVY--GVTKFADLTQNEFSK--------SLGLRTDLRNENEP-PFADAEIPDVQ---LPTEFDWRKKGVVT 1033
TcF_XP_008195656   LNT-FEQGTATY--GITRFADMTQKEFSR--------SLGLRTDLRNENET-PFAQAKIPNIE---LPKEFDWRKKNVVT 1555
TmO12              LNSNKTGDSAVY--GLTKFSDMLPEEFLN-----KHLHLNLCQRFIPHSNT-HHHHHHDKRST---LPQKIDWREKKVVT 136
TcO_XP_970512      LNSKKRNGSALY--GLTKFSDLLPEEFFQ--------TYLQSNLSQKTHSN-EPKRHHHKRAT---VPNKVDWREKNAVT 134
TcK_XP_001814509   HNEEADKGLHSYYIKENDLSDMSTQSYLQ-----KMVKLTKSTHRKVDPEV--VGDLFELLHH---IPEEVNWVEKGFET 132
```

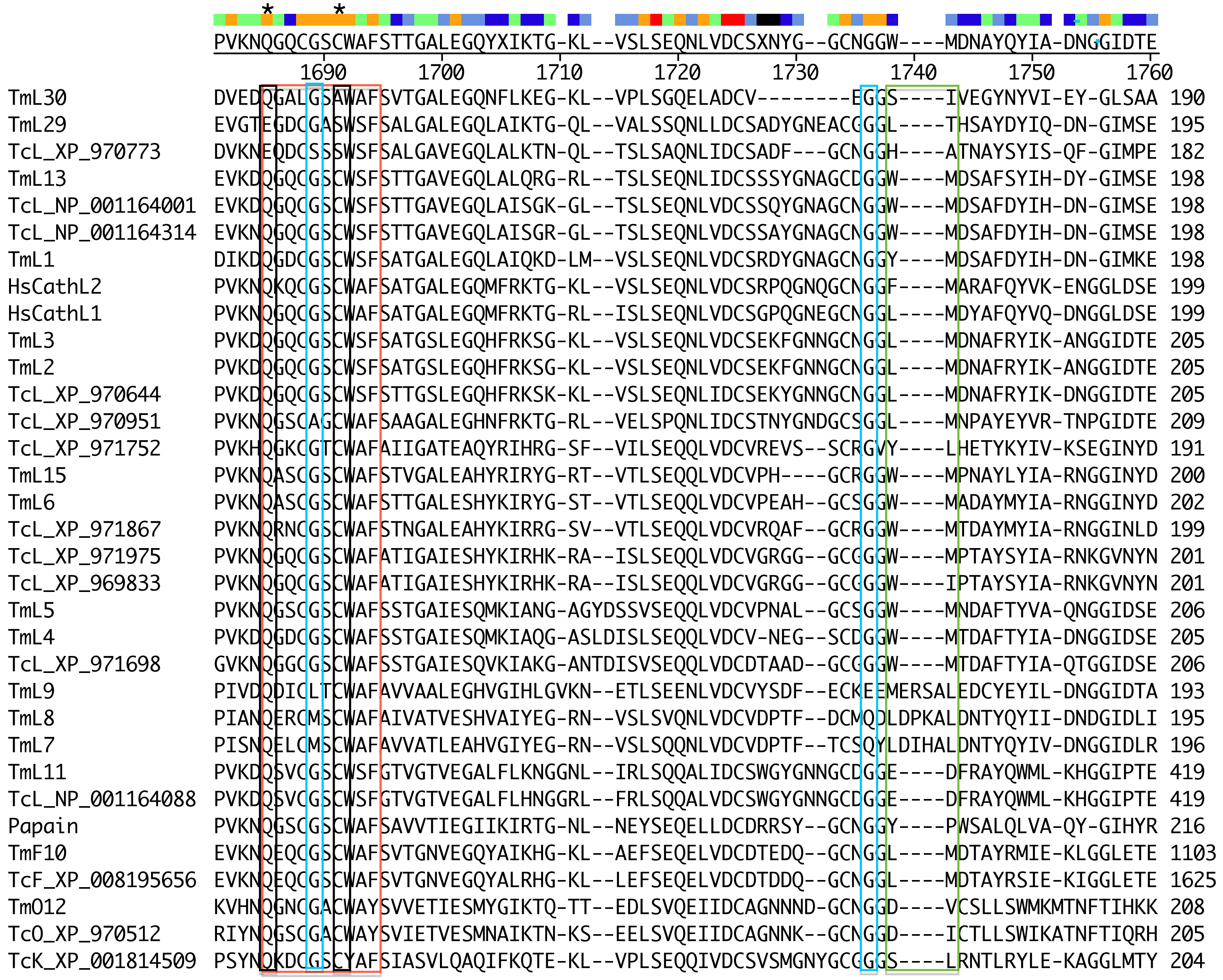

*
*
PVKNQGQCGSCWAFSTTGALEGQYXIKTG-KL--VSLSEQNLVDCSXNYG--GCNGGW----MDNAYQYIA-DNGGIDTE
1690
1700
1710
1720
1730
1740
1750
1760
TmL30 DVEDQGALGSAWAFSVTGALEGQNFLKEG-KL--VPLSGQELADCV--------EGGS----IVEGYNYVI-EY-GLSAA 190
TmL29 EVGTEGDCGASWSFSALGALEGQLAIKTG-QL--VALSSQNLLDCSADYGNEACGGL----THSAYDYIQ-DN-GIMSE 195
TcL_XP_970773 DVKNEQDCSSSWSFSALGAVEGQLALKTN-QL--TSLSAQNLIDCSADF---GCNGGH----ATNAYSYIS-QF-GIMPE 182
TmL13 EVKDQGQCGSCWSFSTTGAVEGQLALQRG-RL--TSLSEQNLIDCSSSYGNAGCDGGW----MDSAFSYIH-DY-GIMSE 198
TcL_NP_001164001 EVKDQGQCGSCWSFSTTGAVEGQLAISGK-GL--TSLSEQNLVDCSSQYGNAGCNGGW----MDSAFDYIH-DN-GIMSE 198
TcL_NP_001164314 EVKNQGQCGSCWSFSTTGAVEGQLAISGR-GL--TSLSEQNLVDCSSAYGNAGCNGGW----MDSAFDYIH-DN-GIMSE 198
TmL1 DIKDQGDCGSCWSFSATGALEGQLAIQKD-LM--VSLSEQNLVDCSRDYGNAGCNGGY----MDSAFDYIH-DN-GIMKE 198
HsCathL2 PVKNQKQCGSCWAFSATGALEGQMFRKTG-KL--VSLSEQNLVDCSRPQGNQGCNGGF----MARAFQYVK-ENGGLDSE 199
HsCathL1 PVKNQGQCGSCWAFSATGALEGQMFRKTG-RL--ISLSEQNLVDCSGPQGNEGCNGGL----MDYAFQYVQ-DNGGLDSE 199
TmL3 PVKDQGQCGSCWSFSATGSLEGQHFRKSG-KL--VSLSEQNLVDCSEKFGNNGCNGGL----MDNAFRYIK-ANGGIDTE 205
TmL2 PVKDQGQCGSCWSFSATGSLEGQHFRKSG-KL--VSLSEQNLVDCSEKFGNNGCNGGL----MDNAFRYIK-ANGGIDTE 205
TcL_XP_970644 PVKDQGQCGSCWSFSTTGSLEGQHFRKSK-KL--VSLSEQNLIDCSEKYGNNGCNGGL----MDNAFRYIK-DNGGIDTE 205
TcL_XP_970951 PVKNQGSCAGCWAFSAAGALEGHNFRKTG-RL--VELSPQNLIDCSTNYGNDGCSGGL----MNPAYEYVR-TNPGIDTE 209
TcL_XP_971752 PVKHQGKCGTCWAFAIIGATEAQYRIHRG-SF--VILSEQQLVDCVREVS--SCRGVY----LHETYKYIV-KSEGINYD 191
TmL15 PVKNQASCGSCWAFSTVGALEAHYRIRYG-RT--VTLSEQQLVDCVPH----GCRGGW----MPNAYLYIA-RNGGINYD 200
TmL6 PVKNQASCGSCWAFSTTGALESHYKIRYG-ST--VTLSEQQLVDCVPEAH--GCSGGW----MADAYMYIA-RNGGINYD 202
TcL_XP_971867 PVKNQRNCGSCWAFSTNGALEAHYKIRRG-SV--VTLSEQQLVDCVRQAF--GCRGGW----MTDAYMYIA-RNGGINLD 199
TcL_XP_971975 PVKNQGQCGSCWAFATIGAIESHYKIRHK-RA--ISLSEQQLVDCVGRGG--GCGGGW----MPTAYSYIA-RNKGVNYN 201
TcL_XP_969833 PVKNQGQCGSCWAFATIGAIESHYKIRHK-RA--ISLSEQQLVDCVGRGG--GCGGGW----IPTAYSYIA-RNKGVNYN 201
TmL5 PVKNQGSCGSCWAFSSTGAIESQMKIANG-AGYDSSVSEQQLVDCVPNAL--GCSGGW----MNDAFTYVA-QNGGIDSE 206
TmL4 PVKDQGDCGSCWAFSSTGAIESQMKIAQG-ASLDISLSEQQLVDCV-NEG--SCDGGW----MTDAFTYIA-DNGGIDSE 205
TcL_XP_971698 GVKNQGGCGSCWAFSSTGAIESQVKIAKG-ANTDISVSEQQLVDCDTAAD--GCGGGW----MTDAFTYIA-QTGGIDSE 206
TmL9 PIVDQDICLTCWAFAVVAALEGHVGIHLGVKN--ETLSEENLVDCVYSDF--ECKEEMERSALEDCYEYIL-DNGGIDTA 193
TmL8 PIANQERCMSCWAFAIVATVESHVAIYEG-RN--VSLSVQNLVDCVDPTF--DCMQDLDPKALDNTYQYII-DNDGIDLI 195
TmL7 PISNQELCMSCWAFAVVATLEAHVGIYEG-RN--VSLSQQNLVDCVDPTF--TCSQYLDIHALDNTYQYIV-DNGGIDLR 196
TmL11 PVKDQSVCGSCWSFGTVGTVEGALFLKNGGNL--IRLSQQALIDCSWGYGNNGCDGGE----DFRAYQWML-KHGGIPTE 419
TcL_NP_001164088 PVKDQSVCGSCWSFGTVGTVEGALFLHNGGRL--FRLSQQALVDCSWGYGNNGCDGGE----DFRAYQWML-KHGGIPTE 419
Papain PVKNQGSCGSCWAFSAVVTIEGIIKIRTG-NL--NEYSEQELLDCDRRSY--GCNGGY----PWSALQLVA-QY-GIHYR 216
TmF10 EVKNQEQCGSCWAFSVTGNVEGQYAIKHG-KL--AEFSEQELVDCDTEDQ--GCNGGL----MDTAYRMIE-KLGGLETE 1103
TcF_XP_008195656 EVKNQEQCGSCWAFSVTGNVEGQYALRHG-KL--LEFSEQELVDCDTDDQ--GCNGGL----MDTAYRSIE-KIGGLETE 1625
TmO12 KVHNQGNCGACWAYSVVETIESMYGIKTQ-TT--EDLSVQEIIDCAGNNND-GCNGGD----VCSLLSWMKMTNFTIHKK 208
TcO_XP_970512 RIYNQGSCGACWAYSVIETVESMNAIKTN-KS--EELSVQEIIDCAGNNK--GCNGGD----ICTLLSWIKATNFTIQRH 205
TcK_XP_001814509 PSYNQKDCGSCYAFSIASVLQAQIFKQTE-KL--VPLSEQQIVDCSVSMGNYGCGGGS----LRNTLRYLE-KAGGLMTY 204

```
                        SDY-PYEAXD-GSCRFDXSKS-XATITGYVDI-TSGDEEALKDAVATVGPVSVAIDAXGE-FQFYSGGVY--YXPTCS--
                                  1770      1780      1790      1800      1810      1820      1830      1840
TmL30                   ADY-PSDSSD-GTCRRGDIPA-AVTESGYVVI--MGNEEALKDTIAAAGPISVAVDATN--WQFYTGGVF--LDANCS-- 260
TmL29                   SDY-PYEGQQ-GSCRFDSSKS-VTKISGYQDV-DYMSETSLQTTVATIGPVASAVHATDN-LQFYYGGIF--FDDTCNLS 268
TcL_XP_970773           KDY-PYEGKA-GVCRFDASKS-ITTVTGFYDI-DPNDETALQGALAMMGPIAATIEATEE-LQFYKGGIL--LDEKCNSK 255
TmL13                   SAY-PYEAQD-DYCRFDSSQS-VTTLSGYYDL-PSGDENSLADAVGQAGPVAVAIDATDE-LQFYSGGLF--YDQTCN-- 270
TcL_NP_001164001        SAY-PYTAMD-GNCRFDASQS-VTSLQGYYDI-PSGDESALQDAVANNGPVAVALDATEE-LQLYSGGVL--YDTTCS-- 270
TcL_NP_001164314        SAY-PYTASE-GSCRFNPSES-VTSLQGYYDL-PSGDENALKSAVANNGPIAVALDATDE-LQFYSGGVL--YDTTCS-- 270
TmL1                    DAY-PYEGVD-RDCRYDPSRS-VTSLTGYYDL-PSGSESALEDAVSNVGPIAVAIDATAE-LQFYYYGIF--YDETCN-- 270
HsCathL2                ESY-PYVAVD-EICKYRPENS-VANDTGFTVV-APGKEKALMKAVATVGPISVAMDAGHSSFQFYKSGIY--FEPDCS-- 272
HsCathL1                ESY-PYEATE-ESCKYNPKYS-VANDTGFVDI--PKQEKALMKAVATVGPISVAIDAGHESFLFYKEGIY--FEPDCS-- 271
TmL3                    QAY-PYKAED-EKCHYKPKNK-GATDRGYVDI-ESGNEDKLQSAVATVGPVSVAIDASHQSFQLYSGGVY--YEPDCS-- 278
TmL2                    QAY-PYKAED-EKCHYKPKNK-GATDRGYVDI-ESGNEDKLQSAVATVGPVSVAIDASHQSFQLYSGGVY--YEPECS-- 278
TcL_XP_970644           QSY-PYKAED-EKCHYKPRNK-GATDRGFVDI-ESGDEEKLKAAVATVGPISVAIDASHPTFQQYSEGVY--YEPECS-- 278
TcL_XP_970951           DSY-PYEARN-GPCRFRPETV-GAYCTGYVDI-AEGDEQGLEAAIATLGPVSAAMDAGRQSFQFYSDGIY--YDPQCGNR 283
TcL_XP_971752           QDY-RYQSAP-GTCRFRADKP-KITFRKYAYL-TAISEEDLQWIVANVGPVTVSFDGRGKQFKSYSGGVF--YNKTC--- 263
TmL15                   RDY-RYEARD-NACRYRSSMP-RVSVRGYAYL-TGPNEEMLKDMVVTQGPVSVAFDSGGN-FAGYRGGVY--YNPRCS-- 272
TmL6                    RDY-PYEARD-NSCRYSSSRA-RVSVRGYAYL-TGPNEEMLKDMVATQGPVSVAFDAGGN-FAGYSGGVY--YNPSCS-- 274
TcL_XP_971867           RNY-PYKASA-GPCRFQASKP-KVTIRGYAYL-TGPNEEMLKHMVVTQGPVSVAIDASGR-FASYGGGVY--YNPSCA-- 271
TcL_XP_971975           RDY-RYQGRN-GRCRYRSSKP-HIAIRSYAAINNNNNEERVRRLVATKGPVSVAIHVDSRTFNRYKSGVY--NNPSC--- 274
TcL_XP_969833           RDY-PYLGRN-GKCRYRSSKP-HIAIRSYAAINNNNNEERVRRLVATKGPVSVAIHVDSRTFHKYKSGVY--NNPSC--- 274
TmL5                    GAY-PYEMAD-GNCHYDPNQV-AARLSGYVYL-SGPDENMLADMVATKGPVAVAFDADDP-FGSYSGGVY--YNPTCE-- 278
TmL4                    EAY-PYEMKN-GHCHYDVNTV-AARITKYDYL-SVPDEDLLADMVATKGPVAVAFDAEGD-FGYYSGGVY--YDSYCV-- 277
TcL_XP_971698           SSY-PYKGVD-ESCHFMSDKV-AAKLKGYAYL-TGPDENMLADMVSSKGPVSVAFDAEGD-FGSYSGGVY--YNPNCA-- 278
TmL9                    TSY-PYDKEM-EECRFKPENV-GAQIKDYVTI-AEGDEEEMKTVVGTIGPVSVIITVDFT-FMVYQKGVY--YKEDCVNT 266
TmL8                    ESY-PNEYQTVGSCRFKRENV-GATIKAYANI-TEGDEEELKAVVGTVGPVTVTFTTDFA-WDFYHKGVY--YNDDCMNT 269
TmL7                    DSY-PVEYYTVGSCRFKSETV-GATIKAYGNI-TEGDEEELKAVVGTIGPVTVTITTDFS-WDLYREGVY--YNGDCMNT 270
TmL11                   ADYGSYLGQD-GYCHADKVPK-VAKITGYINV-TANDESALRMALFKQGPISVGIDASQRTFSFYSNGVY--YEPKCGNK 494
TcL_NP_001164088        EAYGPYLGQD-GYCHADKVQK-VAKITGYVNV-TTNDENALRLALFKHGPISVAIDASQRTFSFYSNGVY--YEPKCGNK 494
Papain                  NTY-PYEGVQ-RYCRSREKGPYAAKTDGVRQV-QPYNEGALLYSIANQ-PVSVVLEAAGKDFQLYRGGIF--VGP-CG-- 288
TmF10                   QDY-PYDAED-ETCHFNKSLA-RVQLTGAMNI-SHNETDMAKWLVAN-GPISIAINANA--MQFYMGGVSHPYKFLCN-- 1175
TcF_XP_008195656        QDY-PYDAED-EKCHFNRTLA-RVQVTGALNI-SHNETDMAKWLVAN-GPISIAINANA--MQFYMGGVSHPFKFLCS-- 1697
TmO12                   SDY----GKN-SHCGKVSAK--GVQVQDFVCDRLVGSENVMMKLLVGIGPLAVAVNAQT--WQNYIGGVI---EYHCDGD 276
TcO_XP_970512           ADY------G-GKCGRGSA---GVHVRDFMCEGLVGSEDVMLRLLADNGPLAVAINAQT--WQNYIGGVI---EYHCDGD 270
TcK_XP_001814509        SDY-PYLARQ-QRCRFDKHRA-IVNLTTWAVL-PARDERALELAVAKIGPVAASINASPHTFQLYHSGVY--DDVACS-- 277
```

```
                     *                         *
                    TNXLNHAVLXVGYG----X--ENGQDYWLVKNSWGTGWGEQGYIKMARNRNNN---CGIATAASYPVV-
                            1850      1860      1870      1880      1890      1900
TmL30               STELNHGVLAVGYG----V--DDGTDYWIVKNSWGTEWGEDGYIRTARNADNN---CGIATDPTCPIL        318
TmL29               LTDLNHGVLIVGYG----T--ELQYDYWIVKNSWGTDWGENGFYKSIRNYGNN---CGIATSCSYPVL        327
TcL_XP_970773       VPDLNHGVLVVGYG----S--ENGGDFWIVKNSWGSDWGEGGYYRPVRNHGNN---CGIASSATLPIL        314
TmL13               QSDLNHGVLVVGYG----S--DNGQDYWILKNSWGSGWGENGYWTQVRNYGNN---CGIATAASYPAL        328
TcL_NP_001164001    AQALNHGVLVVGYG----S--EGGQDYWIVKNSWGSGWGEQGYWRQARNRNNN---CGIATAASYPAL        328
TcL_NP_001164314    AQALNHGVLVVGYG----S--EGGQDYWIVKNSWGSGWGEQGYWRQARNRNNN---CGIATAASYPAL        328
TmL1                ETDLNHGVLVVGYD----S--ESGQDYWIVKNSWGIRWGEQGYWRQARNRGNN---CGIASQASYPTL        328
HsCathL2            SKNLDHGVLVVGYGFEGAN--SNNSKYWLVKNSWGPEWGSNGYVKIAKDKNNH---CGIATAASYPNV        334
HsCathL1            SEDMDHGVLVVGYGFESTE--SDNNKYWLVKNSWGEEWGMGGYVKMAKDRRNH---CGIASAASYPTV        333
TmL3                ASQLDHGVLVVGYG---TE--DDGTDYWLVKNSWGKSWGDQGYIKMARNRDNN---CGIATEASYPLV        337
TmL2                PSQLDHGVLVVGYG---TE--DDGTDYWLVKNSWGKSWGDQGYIKMARNRDNN---CGIATEASYPLV        337
TcL_XP_970644       SEQLDHGVLVVGYG---TD--EDGNDYWLVKNSWGDSWGDQGYIKMARNRDNN---CGIATQASYPLV        337
TcL_XP_970951       PDDVNHAVLVVGYG---TE--PNGQKYWLVKNSYGPQWGIGGYVKLAKDANNH---CGIAIQASYPLV        343
TcL_XP_971752       TRMKTHVAVLVGYG----T--ENGEDFWLVKNSYGPQWGLDGYVKIARNRNNH---CGITNRITYPIFD       322
TmL15               TNKFTHAVVIVGYG----R--ENGQDYWLVKNSWGSNWGQGGYVKMARNRGNN---CGIASQASY           327
TmL6                TNQFTHAVVIVGYG----T--ENGQDYWLVKNSWGSNWGQGGYIKMARNRGNN---CGIASKASYPVF        332
TcL_XP_971867       RNKFTHAVVIVGYG----R--ENGQDYWLVKNSWGRDWGLGGYIKMARNRNNH---CGIASKASYPVF        329
TcL_XP_971975       RGGLNHAVLIVGYG----R--ERGVDYWLVKNSWGAGWGQKGYVKMARNRRNQ---CGIATHASYPVF        332
TcL_XP_969833       RGGLNHAVVIVGYG----R--ERGVDYWLVKNSWGAGWGQKGYVKMARNRRNQ---CGIATHASYPVF        332
TmL5                TNKFTHAVLIVGYG----N--ENGQDYWLVKNSWGDGWGLDGYFKIARNANNH---CGIAGVASVPTL        336
TmL4                TDSFTHAVLIVGYG----N--ENGQDYWLVKNSWGPEWGLEGYFKIARNANNH---CGIASKASIPVL        335
TcL_XP_971698       TNKFTHAVLIVGYG----N--ENGQDYWLVKNSWGDGWGEHGYFKIARNKGNH---CGIASKASYPVLA       337
TmL9                KEPYNHAVTVVGYG----T--EDGQDYWLVKNEWGLTFGDQGYIKMARNRENN---CGIATYATYPIV        325
TmL8                DEIFNHAAVVVGYG----N--EDGEDYWLVKNSWGTYFGQDGYIKMARNRDNN---CGIASRARYPFM        328
TmL7                DEIFNHAVVVIGYG----S--EDGEDYWLVKNSWGTYFGQDGYIKMARNRDNN---CGIASIARYPLI        329
TmL11               VDELDHAVLAVGYG----T--INGENYWLIKNSWSNYWGNDGYILMS-SKENN---CGVMTTPTYVTM        552
TcL_NP_001164088    IDELDHAVLAVGYG----T--INGENYWLVKNSWSNYWGNDGYILMS-SKDNN---CGVMTTPTYVTM        552
Papain              -NKVDHAVAAVGYG----------PNYILIKNSWGTGWGENGYIRIKRGTGNSYGVCGLYTSSFYPVKN       345
TmF10               PKNLDHGVLIVGYGVHNYPLFKKTLPYWIVKNSWGTSWGEQGYYRVYRGDGT----CGLNQTPSSAIVA       1239
TcF_XP_008195656    PKNLDHGVLIVGYGVHNYPLFKKSLPYWIVKNSWGTGWGEQGYYRVYRGDGT----CGLNQTPSSAIVA       1761
TmO12               VSSLNHAVQIVGYD----L--TAQIPHYIVRNTWGEDWGDGGYLYIAVDKNM----CGVANEVSAVTVL       335
TcO_XP_970512       PSKLNHAVQIVGYD----L--TASIPHYIVRNTWGVDFGDGGFLYIAVDKNM----CGIANEVSALRVL       329
TcK_XP_001814509    SNHVNHAMLIVGYT----------KNAWILKNWWGKHWGEKGYMRLRRGKNR----CGIANYAAYALV        330
```
