# Supplementary material for: Functional analysis of C1 family cysteine peptidases in the larval gut of Тenebrio molitor and Tribolium castaneum
Source: BMC Genomics. 2015 Feb 14;16(1):75. doi: 10.1186/s12864-015-1306-x (PMC4336737; doi:10.1186/s12864-015-1306-x)
Supplement: Additional file 3: Figure S2. — Alignment of amino acid sequences of cysteine cathepsin B from T. molitor and T. castaneum, Homo sapiens cathepsin B (HsCathB, AAH10240), and papain (Carica papaya, AAB02650). Conserved residues are marked with a black asterisk and black box; residues in the S1 subsite are in turquoise box; residues in the S2 subsite are in green box. The occluding loop region is bracketed in black lines. Homologs are marked with red asterisk and sequences lacking a signal peptide are underlined in red on first page; the signal peptide recognition site is marked by a red vertical line. [file 12864_2015_1306_MOESM3_ESM.pdf]

```
                  -----MKXXLLVVXLLLTL-----------------------------------SLSWAKTEI--LSDE-----------
                           10        20        30        40        50        60        70        80
TmB27             -----MKIGLLVVATISTL-----------------------------------PYCWSKTEI--LSDK----------- 28
TmB26             -MQKKMKITILVVIAISTL-----------------------------------SYCWAKTEI--LSNK----------- 32
TmB25             -----MKITILVVIAISTL-----------------------------------SYCWAKTEI--LSNK----------- 28
TmB24             ---------ILVVVAVSTL-----------------------------------SYCWAKTEI--LSDE----------- 24
TcB_XP_966750     -----MRSYLVVVFVLTFS-----------------------------------SALSAQNPI--LSDE----------- 28
TcB_XP_966663     -------MKSLVLLLALFL-----------------------------------PKSSPKTPI--LSQQ----------- 26
*TmB32            -MALTNWTRGLVAITIIAVGSALMDDYFDLPGPYCRQRGCCPGRQDDCSVPILGTLCYCDDFCNRTRNEDCCPDFWSHCR 79
*TcB_XP_008195382 -MASFSWLCPLVALTLLTLTTAQFSDYSDLRGPYCQRRGCCPGRQDDCSEPILGTLCYCDDFCNRTRNEDCCPDFWTHCK 79
Papain            MAMIPSISKLLFVAICLFVYM---------------------------------GLSFGDFSIVGYSQN----------- 37
TmB28             -----MRHAFLLYALTIF------------------------------------SLCEVK-KCQYLTKT----------- 28
TcB_XP_008196465  -------MRKIIILFALTL-----------------------------------PFSWSKPSTPSLQPQ----------- 28
TcB_XP_008196467  -------MHKITFLCALTL-----------------------------------PLSWSKPNTSSLQ------------- 26
*TcB_XP_968689    --------MQKLLLLCALL-----------------------------------PLSWTKPNAS---------------- 22
TcB_XP_968767     --------MQKIIILCALL-----------------------------------PLSWSNPNVTL--------------- 23
TmB23             -----MICPLLVSVLSLVV-----------------------------------SPCLQE-EIQMTRHE----------- 29
TmB22             ------MNYSLVSTLILXV-----------------------------------LFTTXVTLISPRRS------------ 28
TmB19             -----MNYIIVSTLLCLTL-------------------------------------LDTPATLASSRRS----------- 28
TmB20             -----MKIFLSFVVLVAVL-----------------------------------SASLAEIDV--LSSE----------- 28
TcB_XP_974244     -------MYFLIFLLLASI-----------------------------------SVSRAEIDI--QSQD----------- 26
TmB33             -----MKCVLLCIVVLASV-----------------------------------ALSYGGVKLHPLSDE----------- 30
TcB_XP_974298     -----MKCVLLCAVVLATI-----------------------------------ALSYGGLNPHPLSDE----------- 30
TmB18             -----MKCVLLFVAVLGTV-----------------------------------ALTHGGVRVHPLSDE----------- 30
TcB_NP_001164205  -----MKYFLHFAVVLATV-----------------------------------ALVYGGVHLHPLSDD----------- 30
TmB17             -----MKNLAYIAVLCTAI--------------------------------------STILAVNPLSKE----------- 27
TcB_XP_974220     -----MRTLPILTIICTAA--------------------------------------SLSVAVHPLSKE----------- 27
HsCathB           -----MWQLWASLCCLLVL-----------------------------------ANARSRPSFHPVSDE----------- 30

                  -----------------------------------------------------------FINSINSKQST--WXAGR--N
                           90       100       110       120       130       140       150       160
TmB27             -----------------------------------------------------------FIESINQKRTT--WVARR--N 44
TmB26             -----------------------------------------------------------FIESINQKRST--WVARR--N 48
TmB25             -----------------------------------------------------------FIESINQKRST--WVARR--N 44
TmB24             -----------------------------------------------------------FIESINQKRST--WVARR--N 40
TcB_XP_966750     -----------------------------------------------------------FINSINAQQST--WTAGR--N 44
TcB_XP_966663     -----------------------------------------------------------FINAINQKHPS--WLAGP--N 42
TmB32             GVTPPP-IAAPIIGCTYEGTVYPIGKQLKKNCNVCKCERVGMS-GDMLCEQHRCIIEPTITDAINSDPSN-GWTASNYSE 156
TcB_XP_008195382  GLPPPPEPPSPILGCTYEGRVYPLNKQIKKNCNVCKCEKMGQNQADMLCEQHQCLIEPSITEAINSNYANYGWSASNYSK 159
Papain            ------------------DLTSTERLIQLFESWMLKHNKIYKNIDEKIYRFEIFKDNLKYIDETNKKNNSY-WLGLN--V 95
TmB28             -----------------------------------------------------------FIDFINKNQSS--WRAGK--F 44
TcB_XP_008196465  -----------------------------------------------------------LIQEINSRQTS--WKAG---- 43
TcB_XP_008196467  -----------------------------------------------------------VIQEINSEQIS--WKAE---- 41
TcB_XP_968689     -----------------------------------------------------------LINQINSQQSA--WTAGI--- 38
TcB_XP_968767     -----------------------------------------------------------LINQINSQQSS--WTARI--- 39
TmB23             -----------------------------------------------------------LINFINTNQSN--WTAGD--N 45
TmB22             -----------------------------------------------------------LINRVNIIQST--WVAGE--N 44
TmB19             -----------------------------------------------------------FINRINNVSST--WVAGE--N 44
TmB20             -----------------------------------------------------------FIDSINRIQSS--WVAGR--N 44
TcB_XP_974244     -----------------------------------------------------------FIDSINQKQSH--WVARR--N 42
TmB33             -----------------------------------------------------------FINEINSKQTT--WKAGR--N 46
TcB_XP_974298     -----------------------------------------------------------FINAINSKKTT--WKAGR--N 46
TmB18             -----------------------------------------------------------FIASINNQKTT--WKAGR--N 46
TcB_NP_001164205  -----------------------------------------------------------FINRINSRKST--WKAGR--N 46
TmB17             -----------------------------------------------------------FIQSINDAQST--WKAGP--N 43
TcB_XP_974220     -----------------------------------------------------------FIQQINEKQST--WKAGP--N 43
HsCathB           -----------------------------------------------------------LVNYVNKRNTT--WQAGH--N 46
```

```
                     *        *
                   FPEN-TPIEXLKRLNGFLG-DPDPXYKLP-LKVHKXDX-NAIPESFDAREKWPQC-DSIGNIRDQGSCGSCWAFGAVEVM
                         170       180       190       200       210       220       230       240
TmB27              FPED-TPIEQLKRLNGALD-DLRHKYQVK-LKVHRVNV-SAIPDTFDGRTYWSQC-ESLKDIRNQGNCGSCWVFGGVEAI 119
TmB26              FPED-TPIEQLRRLNGAIE-DPHYKSQIK-LKVHKVDV-SAIPDTFDGRTHWPQC-ESLKNIRNQGQCGSCWAFGSTEAM 123
TmB25              FPED-TPIEQLRRLNGAIE-DPHYKSQVK-LKVHKVDV-SAIPDTFDGRTHWPQC-ESLKNIRNQGQCGSCWAFGSTEAM 119
TmB24              FPED-TPIEELRRLNGAIE-DPHYKSQLK-LKVHKVNA-SEIPDTFDGRTYWPQC-ESLKTIRNQGGCGSCWAFSTVEPM 115
TcB_XP_966750      FPED-TPIEHLKRLNGALI-TPDLVGKNQ-THVINVIP-EAIPETFDGRTHWSQC-PSLKNIRNQGNCGSCWAFGSVEVM 119
TcB_XP_966663      FPPN-TPHSHLRSLNGARD-DPAFFTDTE-TKNVTI-P-EQIPQNFDARIVWPQC-ESIRKIRNQGSCGSCWAFGAVETM 116
TmB32              FWGR-TLEEGIKLRLGTLQ-PQRFVMKMT-PVRHTYDP-QSFPREFESEMKWP---RWISGIQDQGWCGSSWAISTAAVA 229
TcB_XP_008195382   FWGH-KLEEGIKLRLGTLQ-PQRFVMHMN-PVRRIYDP-NSLPREFDSEFKWP---GWMSEIQDQGWCGSSWAITTAAVA 232
Papain             FADM-SNDEFKEKYTGSIA-GNYTTTELS-YEEVLNDGDVNIPEYVD----WRQK-GAVTPVKNQGSCGSCWAFSAVVTI 167
TmB28              FLENVTSRNFVHRYHRPVL---NVGNEVR-VKDCYLED-LHIPDSFDARKEWPHCADVIGNIRDQGSCSSCWAFSSTGVM 119
TcB_XP_008196465   -----TNSLDIKSRLGFLGLHPDPDYKIQ-TKHHKIA--KSIPESFDAREKWPECKDVIGKIRDQGTCGSCWAFASTEVM 114
TcB_XP_008196467   -----TNCLDIKSRLGFLGLHPDPNYKIQ-TKQHKISRIISIPESFDAREKWPECKDVIGKIRNQGNCGSCWAFASTEVM 114
TcB_XP_968689      -----NPFDDIESRLGFLGIHPDPNFKPE-IKEPQATQ-NVIPETFDAREYWPECADIIGNIRNQGKCSSSWAFAAAEVM 110
TcB_XP_968767      -----NPFDDIESRLGFLGIHPDPNFQLE-VLEWEEPR-TVIPATFDAREYWPQCKDVIGNIRNQGKCGSCWAFAAAEVM 111
TmB23              FPEI-TSTEVLKRLNGVLGIDPDPEYQVT-LRRHKIGK-TKMPEFFDARDAWAECSGVIGYVRDQGQCGSCWAFAAAEVM 122
TmB22              FPDS-TTTEDLKHLNGFLGVDPDPDFTIQ-KKTHVIPR-NEIPARFDAREKWPECRGIIENIMDQGXCGSCWAFSSAEVM 121
TmB19              FPDS-IPTEHLHYLNGFLGIDPDPNFIIA-ENTHVIPR-NQIPAEFDAREAWPECRDIIENIMAQGACGSCWAFASTEVM 121
TmB20              FPEN-TTNEYLYKLNGFIGLHPDPNYKPP-VLVHTFNA-RDVPESFDARTKWPNC-DSLNRIRDQGACGSCWAFASIESM 120
TcB_XP_974244      FPEN-TTNEYLYKLNGFLGLHPDPNYMPE-KIKHNFNP-QDIPKTFDARKKWPKC-DSLNRIRDQGSCGSCWAFAAVETM 118
TmB33              FDVN-TPISHVRRLLGVLP-KKANAPKLP-VKTHAVNL-DAIPESFDAREAWPECTSIIGEIRDQASCGSCWAFGAVEAM 122
TcB_XP_974298      FDIH-TPLANIKKLLGVLP-KKANARQLE-LKVHSVDV-NAIPESFDAREAWPECASIIGDIRDQASCGSCWAFGAAEAM 122
TmB18              FDVN-TPLPQIKKLLGVLP-KTENTPKLP-LKIHPEGV-LDIPESFDAREAWPDCASIIGEIRDQASCGSCWAFGAVEAM 122
TcB_NP_001164205   FDID-TPISHIKQLLGVLP-ETENTPKLP-KKIHSINA-QEIPDSFDAREAWPDCAPIIGNIRDQSTCGSCWAFGAVEAM 122
TmB17              FAEN-VDMSYIRRLMGV---HPHSKYHKPSIKTHQLDA-TAIPASFDARQEWPNC-PTIREIRDQGSCGSCWAFGAVEAM 117
TcB_XP_974220      FAEN-VPMSYIRRLMGV---PPNSKYHMPSVKRHLLDA-MEIPDDFDARKQWPNC-PTIREIRDQGSCGSCWAFGAVEAM 117
HsCathB            FY-N-VDMGYLKRLCGTFLGGPKPPQRVM-FTED-----LKLPASFDAREQWPQC-PTIKEIRDQGSCGSCWAFGAVEAI 117

                                                                                  Occluding Loop
                   SDRLCIASNGTVKFEFSAEDLLSCCTD-CGD---GCN-GGYPARAWDYWINSGIVSGGDY--NSNEGCQPYS---CSAXV
                         250       260       270       280       290       300       310       320
TmB27              TDRICIATGGKVKFQFSAEDVLSCCTD-CGD---GCD-GGYPYSTWTYWINSGIVSGGDY--QSQQGCQPYT---KSAFV 189
TmB26              TDRLCIATDGKVKFQFSPEDLLSCCKE-CGD---GCD-GGSSATAWAYWMNSGIVSGGDYQSQSQEGCQPYL---ESTFV 195
TmB25              TDRLCIATNGKV----------ACCTA-CGD---GCD-GGWPSKSFDYWMSSGIVSGGDY--QSQQGCQPYA---KSAFD 179
TmB24              TDRLCIATNGTVKFEFSAEDLLSCCTD-CGK---GCQ-GGAPSKAWAYWKSSGIASGGDY--QSQQGCQPYA---KSAFI 185
TcB_XP_966750      TDRLCIASKGKTKFEFSADDLLACCTA-CGK---GCD-GGAPYRAFEYWVAKGIVSGGDY--NSNEGCQPYE---GSAFL 189
TcB_XP_966663      SDRLCIASNATKKFEFSAQDLLACCKE-CGH---GCG-GGYSSRAWQYWVTDGIVSGGDF--NTSQGCHPYS---VQAFR 186
TmB32              SDRFAILSKGREQIMLSAQHLVSCDIR-GQQ---SCN-GGYLDRAWSYIRKFGLV---------DEQCFPYTASNEKCKV 295
TcB_XP_008195382   SDRFAILSKGREKVTLSAQHLLSCDRR-GQQ---SCN-GGYLDRAWSYIRKIGLV---------DEQCFPYSATNEKCRI 298
Papain             EGIIKIRTGNLN--EYSEQELLDCDRR--SY---GCN-GGYPWSALQLVAQYGIHYRNTY---PYEGVQRY----CRSRE 232
TmB28              TDRLCIQSEGEVKFQFSPQDILSCCEN-CSNSTYGCAVGGSSDRAWRYWTESGIVSGGDY--DSYQGCVFYQ---ESEFY 193
TcB_XP_008196465   TDRLCIGTKGETKFVFSPENLLTCCED-CRL---ECV-GGYTAKAWDYYINEGIVSGGDY--NSSEGCQPYS---KASFQ 184
TcB_XP_008196467   TDRLCISSK-----------------D-CGC---GCK-GGYIKNAWDYYINEGIASGGDY--NSSEGCQPYS---ESSFQ 167
TcB_XP_968689      SDRLCIATNGKVKIQLSPEDLIDCCHY-CGN---QCK-GGYTYYAWNYFMLTGLVSGGDY--NTSTGCQPYS---ELNYY 180
TcB_XP_968767      SDRLCVATNGSVKFEFSPEDLINCCET-CGK---KCK-GGYSYYAWKYYTSTGLVSGGDY--NTSRGCQPYS---KSNFN 181
TmB23              SDRLCIATGGQDKVMLSPEDLLQCCTD-CGL---GCD-GGYVNKAWEYWLTYGVVSGGDY--NSNTGCKPYS---ALAYL 192
TmB22              SDRLCIASNGTEKFVFSPEDLLECCVN-CRN---QCL-GGYMNRAFQYWIKNGVVSGGGY--NSNLGCKPYS---GSAYI 191
TmB19              SDRLCISSKATKKFVFSPEDLLECCTS-CRD---QCE-GGYINSAFKYWINNGVVSGGDY--NSGLGCKPYS---KEAHI 191
TmB20              SDRICIHSSGSAQFMFSPEDLLSCCTS-CGD----CG-GGYMMSALDFYINEGIVSGGDV--NSNEGCRPYT---ADAHD 189
TcB_XP_974244      SDRICIHSSGAKKFFFSAEDLLSCCTA-CGS----CS-GGYMMAAFDFYIKQGVVSGGDL--NSNEGCRPYT---ADAHD 187
TmB33              SDRICIHSDASVKVRISAEDLNDCCYD-CGD---GCN-GGWPDLAWSYWSSTGIVTGGLY--GVDEGCKAYSIKPCDHHV 195
TcB_XP_974298      SDRICIHSNATVKVSISTEDLNTCCYE-CGD---GCN-GGWPAEAWAYWAETGIVTGGKY--ETKDGCKAYTVPPCEHHT 195
TmB18              SDRICIHSNATVKVKISAEDLTSCCVL-CGL---GCN-GGWPEMAWLYWSDSGIVTGGNY--GSHEGCRTYSIEPCEHHV 195
TcB_NP_001164205   SDRICIHSNATVKVNISAEDPLDCCTI-CGM---GCN-GGMPAMAWLHWTVNGIVTGGNY--EDTNGCKAYSFAPCEHHV 195
TmB17              SDRVCIHSKGSINVHVSADDLVSCCVT-CGM---GCN-GGFPGAAWHYWVKTGIVSGGTY--GSHQGCRPYEIAPCEHHV 190
TcB_XP_974220      SDRVCIHSKGAVNVRLSADDLVSCCYS-CGM---GCN-GGFPGAAWHYWVNKGIVSGGSF--GSNQGCRPYEIAPCEHHV 190
HsCathB            SDRICIHTNAHVSVEVSAEDLLTCCGSMCGD---GCN-GGYPAEAWNFWTRKGLVSGGLY--ESHVGCRPYSIPPCEHHV 191
```

```
                    Occluding Loop
                    DGT----------TPXCXKTC-NTGYKTPYXQDKH--FGSS-XYSIS-XNVXQIQTEIMTNG-PVEASFXVY-EDFYSYK
                              330       340       350       360       370       380       390       400
TmB27               DNT----------TPECANTCLNTNYTISYQQDKH--FGVD-HYSIA-EDVEQIQTEILTNG-PVEASYSVY-GDFYSYD 253
TmB26               NGV----------TPECATTCRNXDYKTPXQQDKH--SATE-HYQIA-QNVEQIQTEILNNG-PVSASYTVY-DDFYNYG 259
TmB25               DYI----------TPKCATTCLNTGYKTSYQQDKH--FGTQ-HYRIA-QNVQQIQTEIINNG-PVSASYTVY-EDFYNYD 243
TmB24               DGK----------TPACANTCLNTDYKTPYQQDKH--FGID-HYAIAKKDVLQIQTEILTNG-PVSAMFGVY-DDFYNYY 250
TcB_XP_966750       NSV----------TPKCSTKCLNSKYTTPYAKDKH--YGTDFIYMTS-KNVAEIQTEIMNNG-PVVTHMDVY-EDFYSYK 254
TcB_XP_966663       DST----------TPNCSSFCTNPKYQKNYSEDKR--YGAR-SYRIA-KNIEQIQAEIMTSG-PVQASYVVY-DDFYSYQ 250
TmB32               LRK----------GGLAEAKC-----KLPELVDRRSKYKVAPAYRIG--NETDIMHDIMTSG-PVQATMKVY-HDFFTYK 356
TcB_XP_008195382    PRR----------GDLVTANC-----QLPTNVDRRSKYKVAPAYRVG--NETDIMYEILHSG-PVQATMKVY-HDFFTYK 359
Papain              KGP-------------YAAKTDGVRQVQPYNE------GAL-LYSIA-------------NQ-PVSVVLEAAGKDFQLYR 278
TmB28               LKT----------KSPCMKSCNNPHYKISYAEDKH--FGSD-YYQVK-PAEKCIQAEIMSNG-PVEAIFALY-EDFYYYK 257
TcB_XP_008196465    YAV----------ASKCVKACQNDKYDVKYDDDKH--YGDS-FYTLE-TNVTQIQTEILTNG-PVMATFNVF-EDIIYYK 248
TcB_XP_008196467    YAE----------ASECVKACQNDKYDVNYDDDKH--YGDS-XYTLE-TNVAQIQMEILTNG-PVMAYYNVF-EDFACHK 231
TcB_XP_968689       R-I----------TPPCNTTCQNDKYPIPYVSDKH--FGDS-IYYIP-QNETAIQNEILSGGGPVVAAFDVY-GDFKIYR 244
TcB_XP_968767       DGV----------SPECSKTCQNTKYPTSYLNDRH--FGDG-TYYIL-KNVTTIQQEILLRGGPVMAGFDVY-EDFKLYR 246
TmB23               YKT----------TPPCQKRC-QLNYNTPYEVDKS--FALS-IYKVS-SEEEQIQMEIMTNG-PVMTTYTVF-EDFYSYQ 255
TmB22               NGR----------VSTCTKNC-QVGYTTIYNDDKK--FGGS-VYGVS-AAVDQIQMEIMLNG-PVVAGYQVY-EDFYLYK 254
TmB19               NSR----------IPTCVKNC-QTGYKTSYKDDKK--FGVS-VYGVP-AIVEQIQMEIMLNG-PVIAGYEVY-EDFYLYK 254
TmB20               QGQ----------TPACTKSC-RNGYSTSYSADKH--YGSN-DYVVS-SAIDQIQYEVMTNG-PIIVNFEVF-QDFYNYV 252
TcB_XP_974244       KGV----------TPSCTKSC-RKGYPTSYSSDKH--YGSK-DYIVD-AGVSNIQYEIMTNG-PIIVSFKVY-QDFYNYG 250
TmB33               DGNLGPC-GDIQRTPACKKSC-DSTSDLEYKSDLR--RGSA--YSIP-KSESQIQTEIMTNG-PVEADYDVY-SDFLTYK 266
TcB_XP_974298       EGDLPAC-GDIVPTPQCKKEC-DAGVDIEYKSDLR--KGSA--YQTS-SDESQIQTEIMTNG-PVEADFDVY-EDFLNYK 266
TmB18               DGDLPAC-GDIQPTPACQRVC-DADSDLEYEPDLR--TGSS--YTVG-GSVIQIQTEILTNG-PVEASYEVY-EDFLSYK 266
TcB_NP_001164205    DGDLPPC-GPTKPTPDCKKEC-DSGSSLTYQNDLT--HGSN--YGID-PYPKQIQTEIMTNG-PVEASFSVY-EDFLSYK 266
TmB17               NGTRPPCTGDGNKTPSCKKVC-EKGYNVPYNKDRN--FGKE-AYSIS-SDVQQIQKEIMTNG-PVEGAFIVY-ADLLSYK 263
TcB_XP_974220       NGTRPPCTGDDNKTPSCKQQC-EKGYNVPYKKDKN--FGKE-AYSIS-SEVQQIQKEIMTNG-PVEGAFEVY-EDLLSYK 263
HsCathB             NGSRPPCTGEGD-TPKCSKIC-EPGYSPTYKQDKH--YGYN-SYSVS-NSEKDIMAEIYKNG-PVEGAFSVY-SDFLLYK 263

                                       *                  *
                    SGVYQH---VSGXYVGGHAVKILGWGTENGTP----YWLVANSWGSDWGDLGGFFKILRGXNE----CGIESNIXAGX--
                              410       420       430       440       450       460       470       480
TmB27               SGVYQH---VDGKYAGNHAVKILGWGTEDGTP----YWLVANSWGGNWGGLDGFFKILRGADH----LGIESNILGGT-- 321
TmB26               SGVYQH---VSGDVAGAHAIKILGWGTENGTP----YWLVANSWGADWSSLDGFFKILRGSNE----CGIESN        321
TmB25               SGVYQH---LSGDESGSHAIKILGWGTENGTP----YWLVANSWGADWSSLDGFFKILRGSNE----CGIESNIQAGT-- 311
TmB24               SGVYQH---LSGNRSGNHGVKILGWGTEDGTP----YWLVANSWGTNWAGLGGFFKILRGKNS----AGIELNVMGGT-- 318
TcB_XP_966750       SGVYQH---VSGNSMGGHAVKIIGWGTEKGVP----YWLIANSWGAKWADLDGFYKILRGKNH----CKIETYIYGGT-- 322
TcB_XP_966663       NGVYQH---VLGNVSGRHSVKILGWGRENGTD----YWLVANSWGRDWGRLGGFFKFLRGENH----CDIESNILGGD-- 318
TmB32               TGIYKHSPLSSADRTGYHSVRIVGWGEEYTSQGVKKYWKVANSWGRLWGD-NGYFKIARGTNE----CEIESFVLGTW-- 430
TcB_XP_008195382    RGIYRHSPISTNDRTGYHSVRIVGWGEEYSPEGLKKYWKVANSWGPEWGE-NGYFRILRGSNE----CEIESFVLGTW-- 433
Papain              GGIFVG---PCGNKV-DHAVAAVGYGPN--------YILIKNSWGTGWGE-NGYIRIKRGTGNSYGVCGLYTSSFYPV-- 344
TmB28               DGVYTY---TIGGLVSAHTVKIIGWGVENGNP----YWLAANSWNCNWGQLKGFFKIRRGVDE----CGIEQHVLAGTAR 326
TcB_XP_008196465    SGVYYH---VSGDCT-XHVVKVIGWGTEEGVP----YWLIANSWGTWWGDLGGFIKIKRGTNE----CAIEQEMAAGNVH 316
TcB_XP_008196467    SGVYYY---KSGKFVGRHSVKVIGWGTEEGIP----YWLIANSWGSEWGELGGFFKMRRGTNE----CWIEQEMTAGKVH 300
TcB_XP_968689       DGVYIY---TSGALFGRTAVKIIGWGTENGWA----YWLAANSWGKDWGALGGFFKIRRGTNE----CGFEESIIAGQVR 313
TcB_XP_968767       EGVYVH---TSGALLGSHAVKIIGWGTENGWA----YWLVANSWGKDWGALGGVFKIRRGTNE----CKIEQSIITGHVR 315
TmB23               RGVYHH---VAGKYMGQHAVKIIGWGVENKIP----YWIVANSWGSRWGALGGFFKILRGVNH----CGIEEYVIVSD-- 323
TmB22               KGVYKH---VTGQKMGNHAVKIVGWGTENGTP----YWLVANSWGRNWGDLGGFFKILRGKNH----CGIESNIVTAK-- 322
TmB19               KGVYQY---VTGPKVGNHVVKIVGWGTENDTP----YWLVANSWGRDWGDLGGFFKILRGKNH----CDIENYITAGK-- 322
TmB20               SGVYRH---VSGESVGFHVVKIVGWGVENGMP----YWLIANSWGSSWGD-HGFFKMLRGQNE----CGIENYPYAAM-- 319
TcB_XP_974244       SGVYHH---VSGNYTGNHIVKIVGWGTEKEQD----YWLIANSWGSSWGE-HGFFKILRGKNE----CGIENNPYAVL-- 317
TmB33               SGVYQH---VSGEAEGGHAVKILGWGVEDGTP----YWLVANSWNESWGD-KGYFKILRGQDE----CGIESDIIGGI-- 333
TcB_XP_974298       SGVYQQ---TTGNYAGGHAIKILGWGVEDGTP----YWLAANSWNEDWGD-KGYFKILRGQNE----CGIESDIIGGI-- 333
TmB18               SGVYQH---VSGEEQGGHAVKILGWGVEDGTP----YWLVANSWNEDWGD-KGYFKILRGQDE----CGIEEDITAGL-- 333
TcB_NP_001164205    SGVYQH---LEGEYAGGHAIKILGWGVENDTP----YWLVANSWNEDWGD-KGYFKILRGSNE----CGIEGSIVAGI-- 333
TmB17               SGVYQH---VTGAELGGHAIRILGWGVEKNTP----YWLIANSWNSDWGD-NGIFKILRGKDH----CGIESEIVAGL-- 330
TcB_XP_974220       KGVYQH---VKGEALGGHAIRILGWGTEKGTP----YWLIANSWNSDWGD-NGTFKILRGEDH----CGIESSIVAGI-- 330
HsCathB             SGVYQH---VTGEMMGGHAIRILGWGVENGTP----YWLVANSWNTDWGD-NGFFKILRGQDH----CGIESEVVAGI-- 330
```

```
                        -----------------------PKX------------------------------------
                                 490       500       510       520       530       540
TmB27                   -----------------------PKV                                           323
TmB26                                                                                        321
TmB25                   -----------------------PKV                                           313
TmB24                   -----------------------PKV                                           320
TcB_XP_966750           -----------------------PQV                                           324
TcB_XP_966663           -----------------------PKI                                           320
TmB32                   -----------------------PEIENKLLLNNEIRRIRYPK                          449
TcB_XP_008195382        -----------------------AEVENKLLLRNEI                                 445
Papain                  -----------------------KN                                            345
TmB28                   LKTKTSSSS--------------PWLLVALVSAGIVIAVMAIALFVKKKSMESVINYFRLKS       374
TcB_XP_008196465        IGGRETTTKA-------------PSSGGRIKGAEFGVVLIATILFRHIAIKPW                356
TcB_XP_008196467        IEGNERTEEMTTNATSKA-----PSSGFRTKGPEFRGVLIATILLCQISIKCCLF              350
TcB_XP_968689           --EGGSTSPTPVTFTATVTPPTTPGSGVRVAGCGIGLVL-SMMLFYLLK-----F              360
TcB_XP_968767           KDEKSSTSTTPV-----------PGSGVRVVGSGVGAVL-AMVLFYLLK-----F              353
TmB23                   -----------------------KSHSSQMLPLPLLIV--TLPLVCKVIIFFAKF              352
TmB22                   -----------------------SSSSSSYISSLLFILTAAAVNSTSFLSK                  349
TmB19                   -----------------------SSASSVYICYFLLMLATVINSTFFLSR                   348
TmB20                   -----------------------PRL                                           321
TcB_XP_974244           -----------------------PKL                                           319
TmB33                   -----------------------PEL                                           335
TcB_XP_974298           -----------------------PVV                                           335
TmB18                   -----------------------PNL                                           335
TcB_NP_001164205        -----------------------PEL                                           335
TmB17                   -----------------------PPAQS                                         334
TcB_XP_974220           -----------------------PKDSS                                         334
HsCathB                 -----------------------PRTDQYWEKI                                    339
```
