# Supplementary figures and images for: Functional analysis of C1 family cysteine peptidases in the larval gut of Тenebrio molitor and Tribolium castaneum
Source: BMC Genomics. 2015 Feb 14;16(1):75. doi: 10.1186/s12864-015-1306-x (PMC4336737; doi:10.1186/s12864-015-1306-x)

A

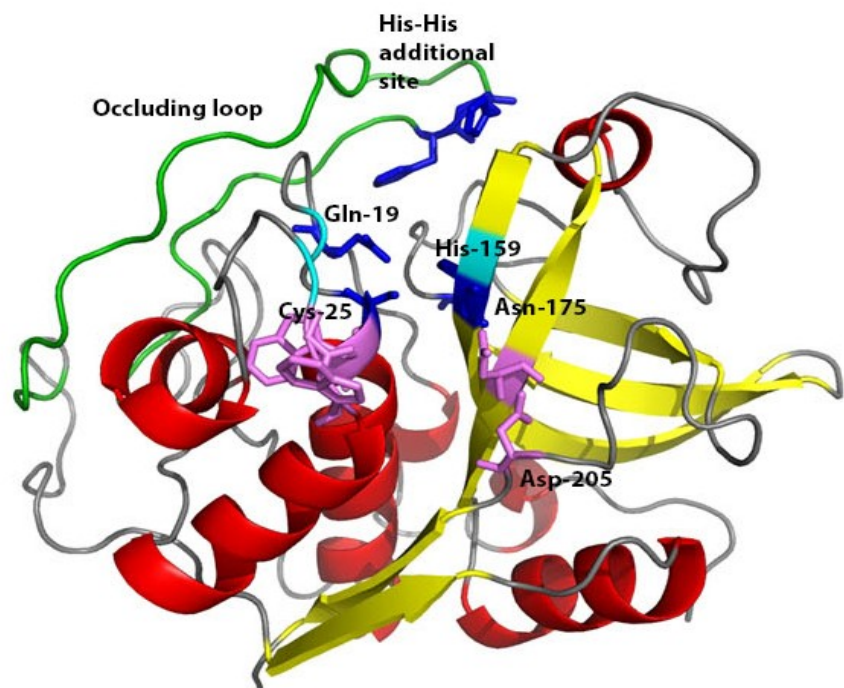

B

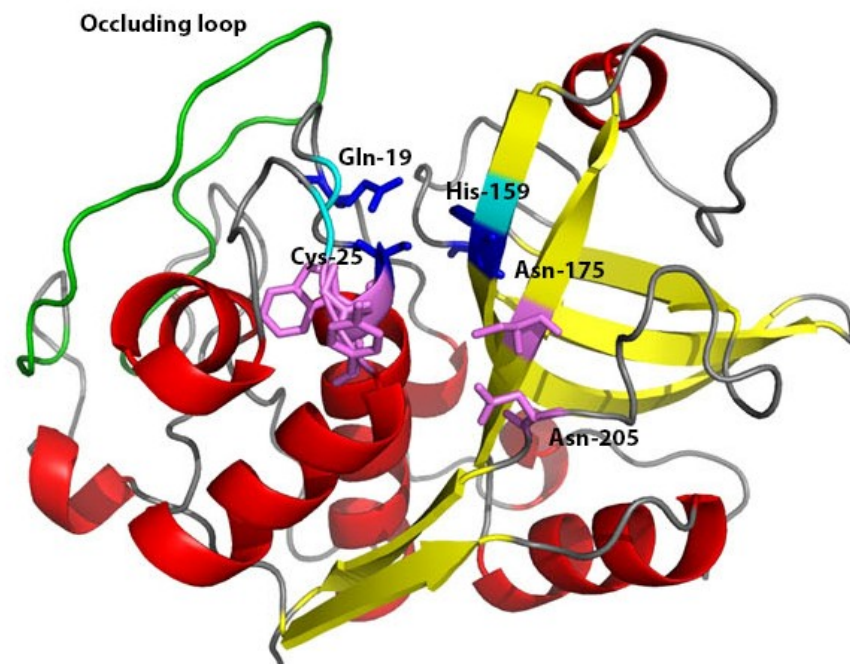

Supplement: Additional file 4: Figure S3 — Models of three-dimensional structures of representatives of two groups of cathepsins B in T. molitor larvae. A, typical cathepsin B TmB33; B, atypical cathepsin B-like TmB22. Dark blue - active site (residues Gln-19, Cys-25, His-159, Asn-175); light blue - S1 substrate binding site (residues 23, 66, 158); purple - S2 substrate binding site (residues 67, 68, 133, 157, 160, 205); the occluding loop is colored green. [file 12864_2015_1306_MOESM4_ESM.pdf]
