# Supplementary material for: Functional analysis of C1 family cysteine peptidases in the larval gut of Тenebrio molitor and Tribolium castaneum
Source: BMC Genomics. 2015 Feb 14;16(1):75. doi: 10.1186/s12864-015-1306-x (PMC4336737; doi:10.1186/s12864-015-1306-x)

Supplemental Fig. S4. Models of two possible conformations (**A** and **B**) of Arg in the P1 position of the substrate FRF in the active site of human cathepsin L [3OF8; Shenoy, Sivaraman 2011].

A.

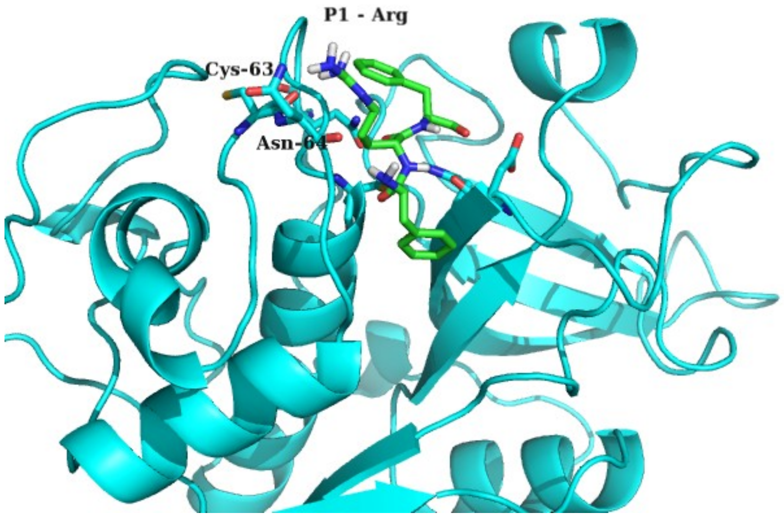

B.

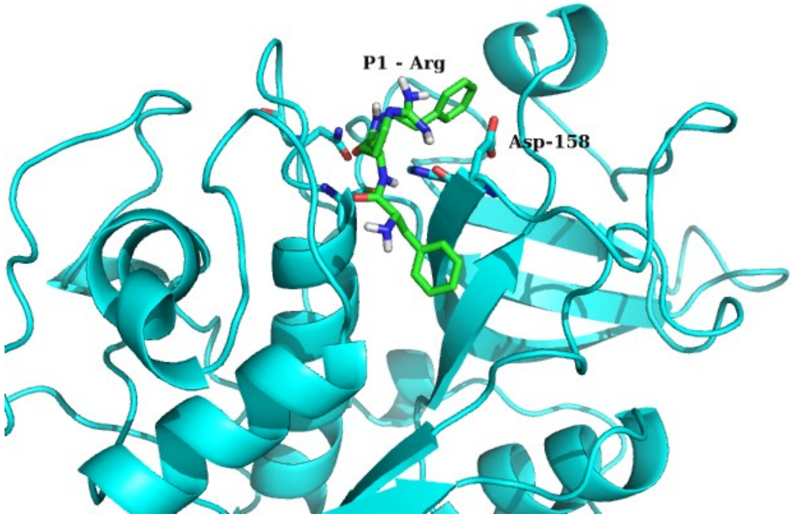

Supplement: Additional file 5: Figure S4. — Models of two possible conformations (A and B) of Arg in the P1 position of the substrate FRF in the active site of human cathepsin L (3OF8) [33]. [file 12864_2015_1306_MOESM5_ESM.pdf]
