# Supplementary material for: Functional analysis of C1 family cysteine peptidases in the larval gut of Тenebrio molitor and Tribolium castaneum
Source: BMC Genomics. 2015 Feb 14;16(1):75. doi: 10.1186/s12864-015-1306-x (PMC4336737; doi:10.1186/s12864-015-1306-x)
Supplement: Additional file 6: Figure S5. — Ramachandran plots of the analysis of active site and S2 subsite of models of T. molitor predicted mature cysteine cathepsins (TmL2, TmL4, TmL5, TmL7, TmL8, TmL9, TmL11, TmL13, TmB18, TmB19, TmB20, TmB22, TmB28, and TmB33). [file 12864_2015_1306_MOESM6_ESM.pdf]

TmL2

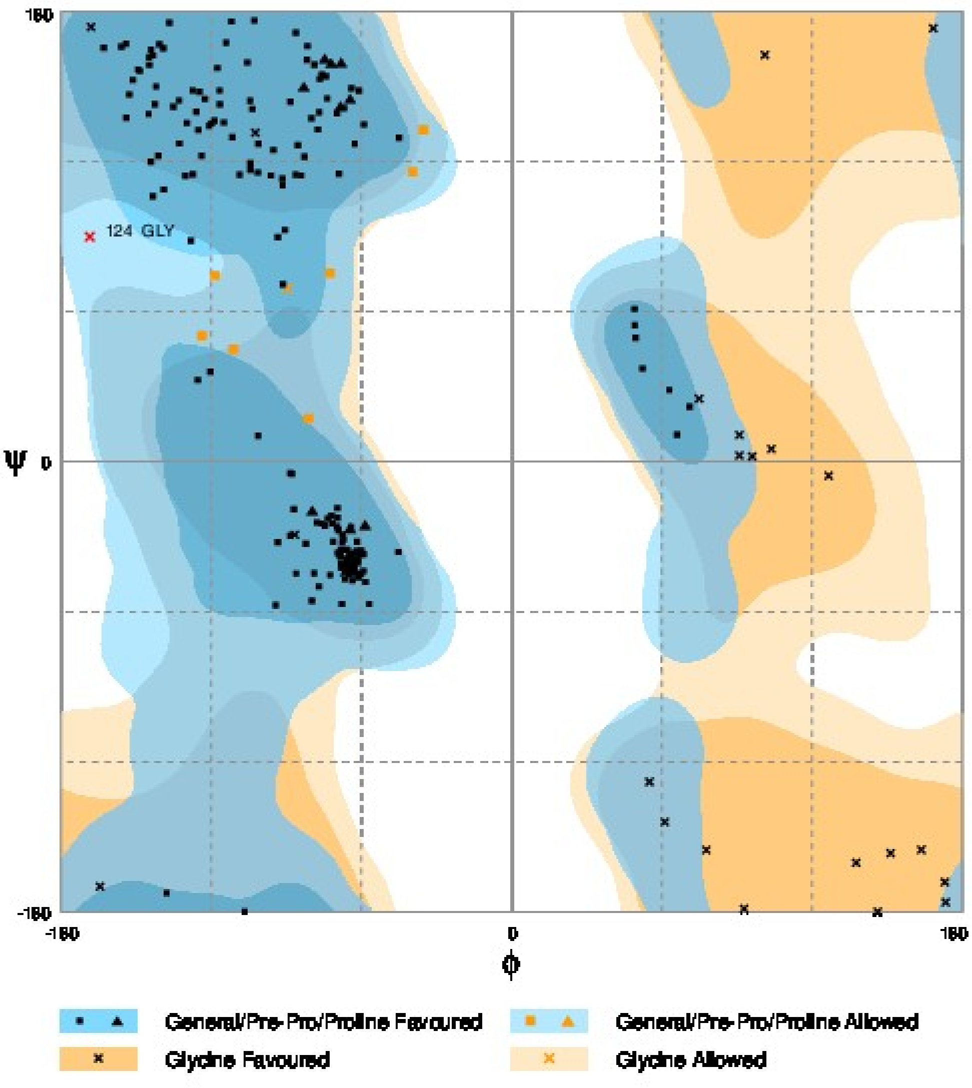

Number of residues in favoured region	(~98.0% expected)	:	213 (95.9%)
Number of residues in allowed region	(~2.0% expected)	:	8 (3.6%)
Number of residues in outlier region		:	1 (0.5%)

TmL4

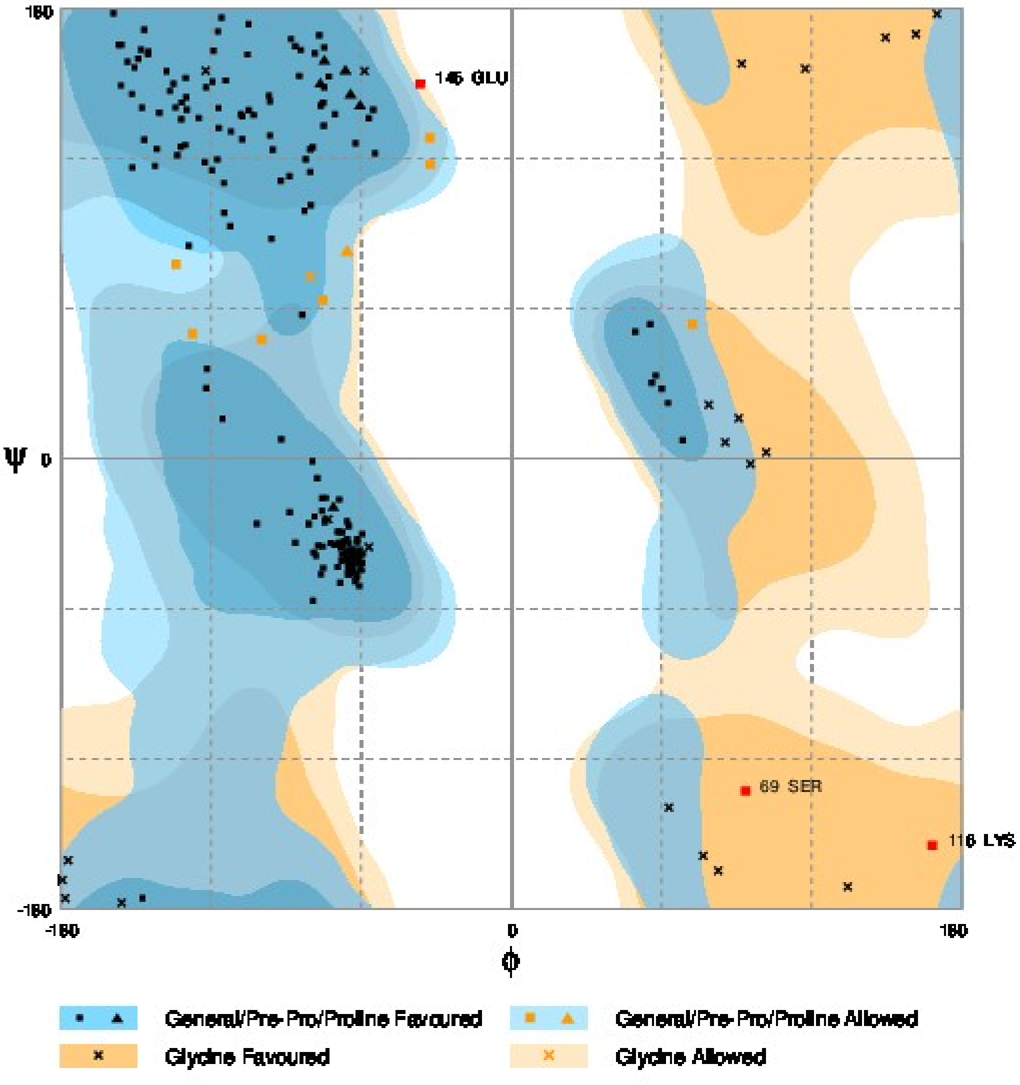


```
Number of residues in favoured region     (~98.0% expected)  :  207 ( 94.5%)
Number of residues in allowed region      ( ~2.0% expected)  :    9 (  4.1%)
Number of residues in outlier region                         :    3 (  1.4%)
```

TmL5

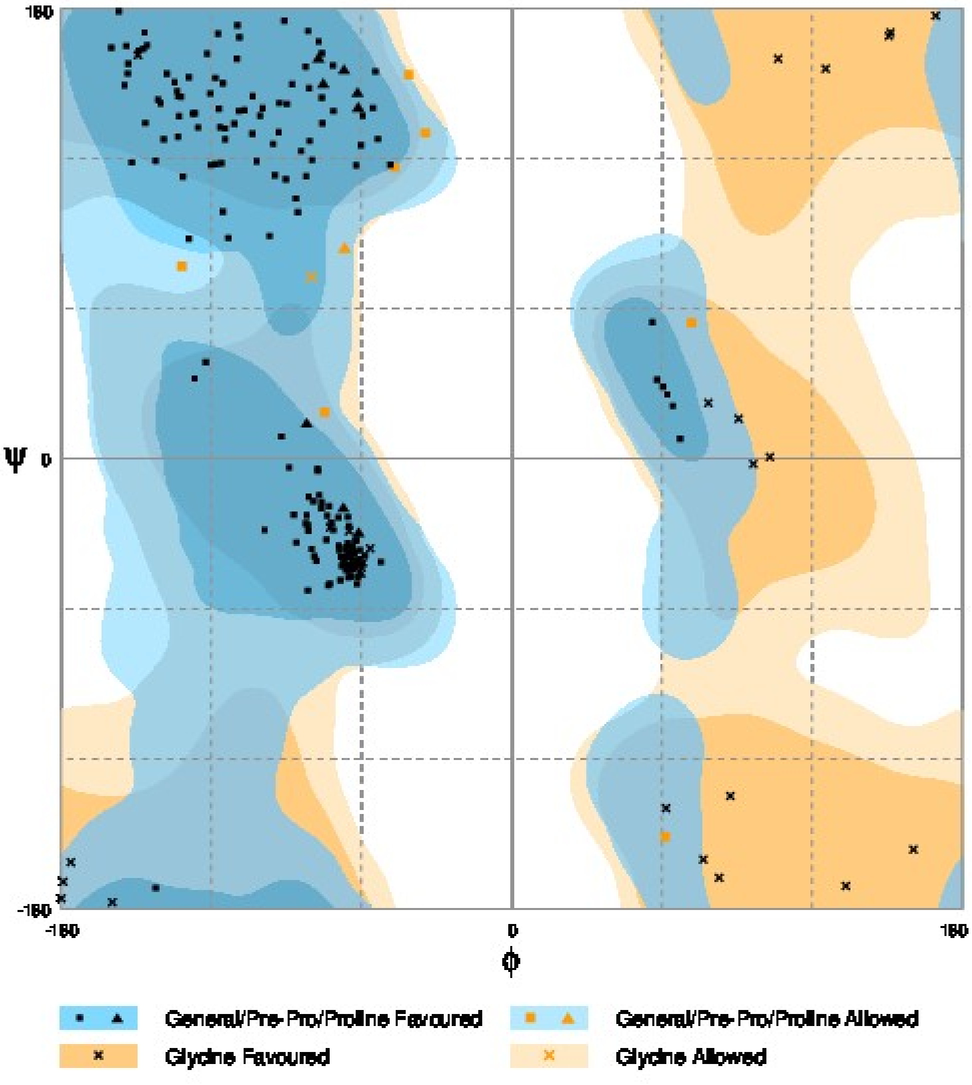


```
Number of residues in favoured region     (~98.0% expected)  :  211 ( 95.9%)
Number of residues in allowed region      ( ~2.0% expected)  :    9 (  4.1%)
Number of residues in outlier region                         :    0 (  0.0%)
```

TmL7

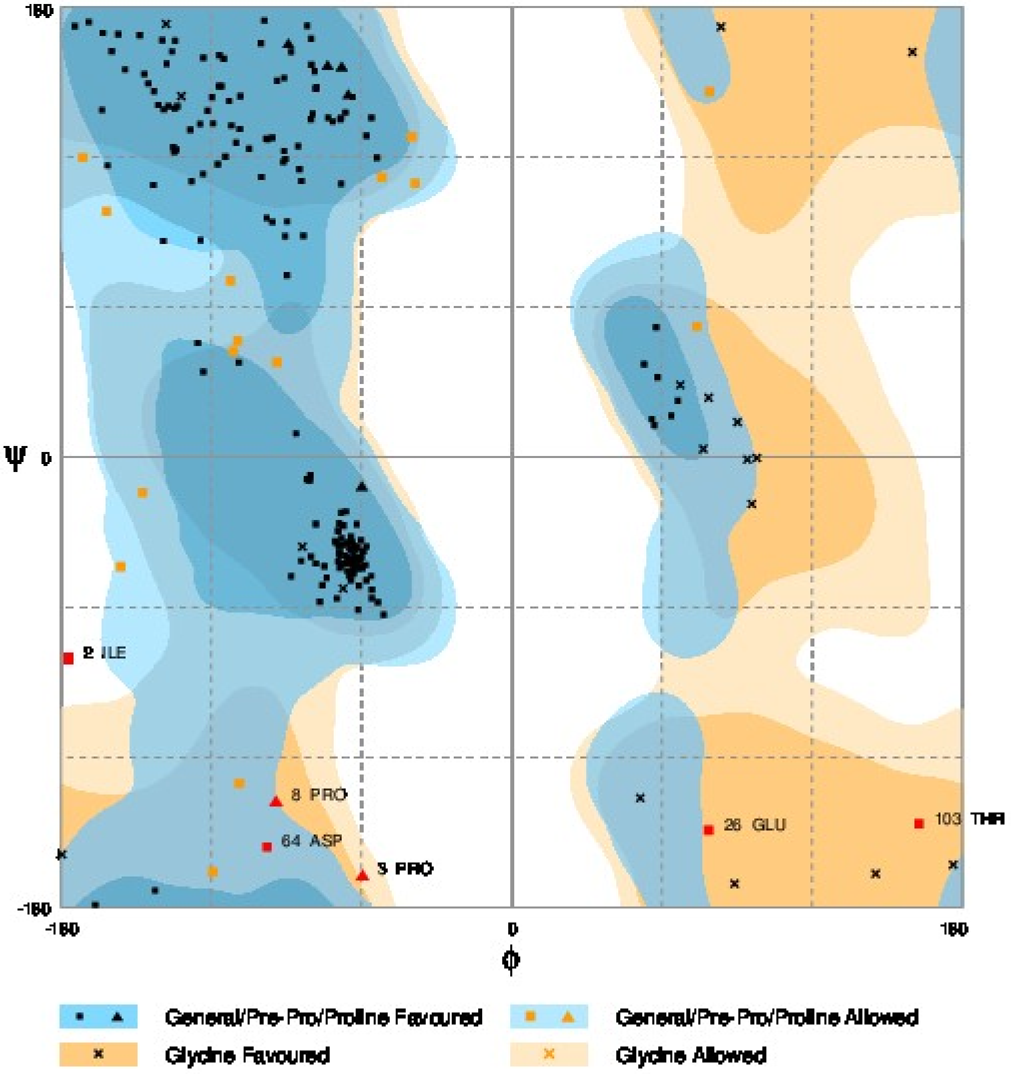


```
Number of residues in favoured region     (~98.0% expected)  :  204 ( 90.7%)
Number of residues in allowed region      ( ~2.0% expected)  :   15 (  6.7%)
Number of residues in outlier region                         :    6 (  2.7%)
```

TmL8

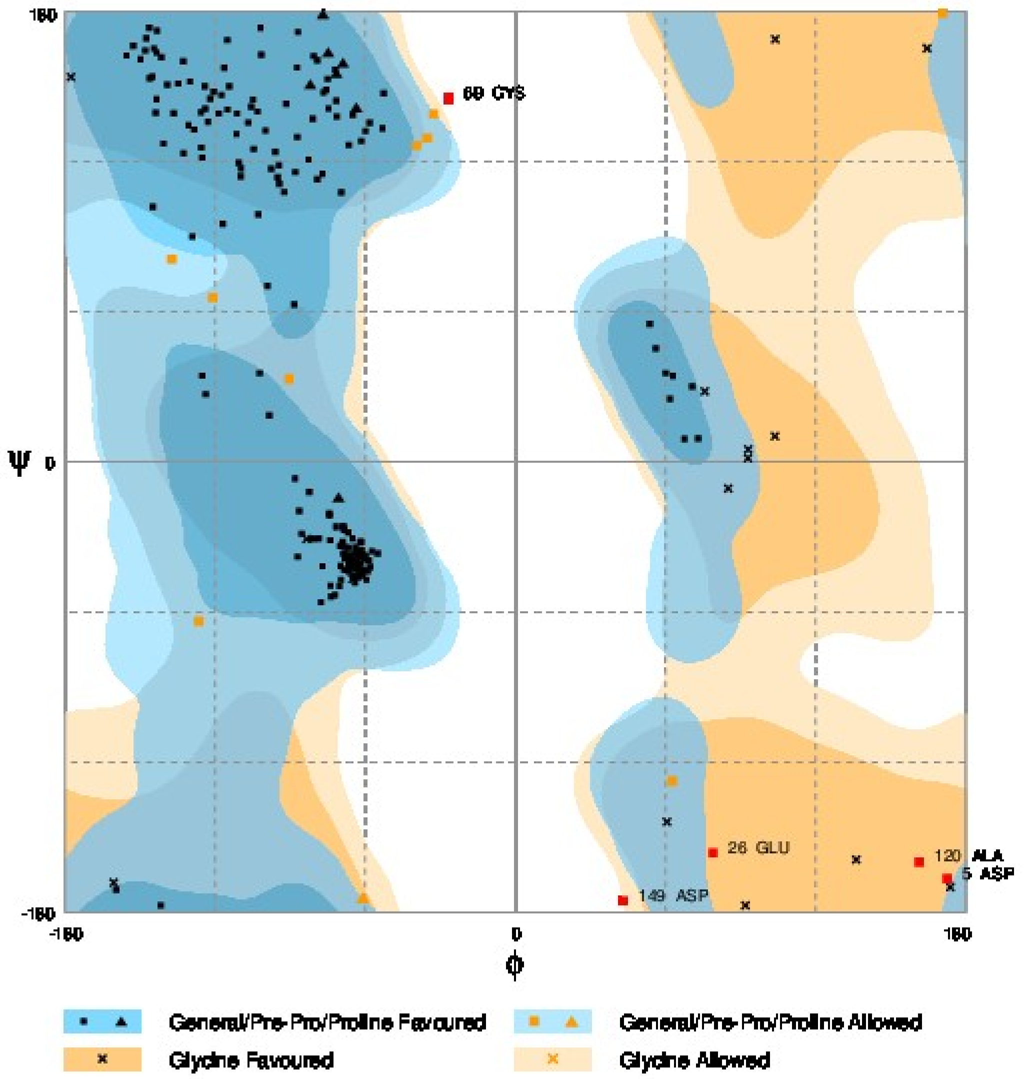


```
Number of residues in favoured region     (~98.0% expected)  :  210 ( 93.3%)
Number of residues in allowed region      ( ~2.0% expected)  :   10 (  4.4%)
Number of residues in outlier region                         :    5 (  2.2%)
```

TmL9

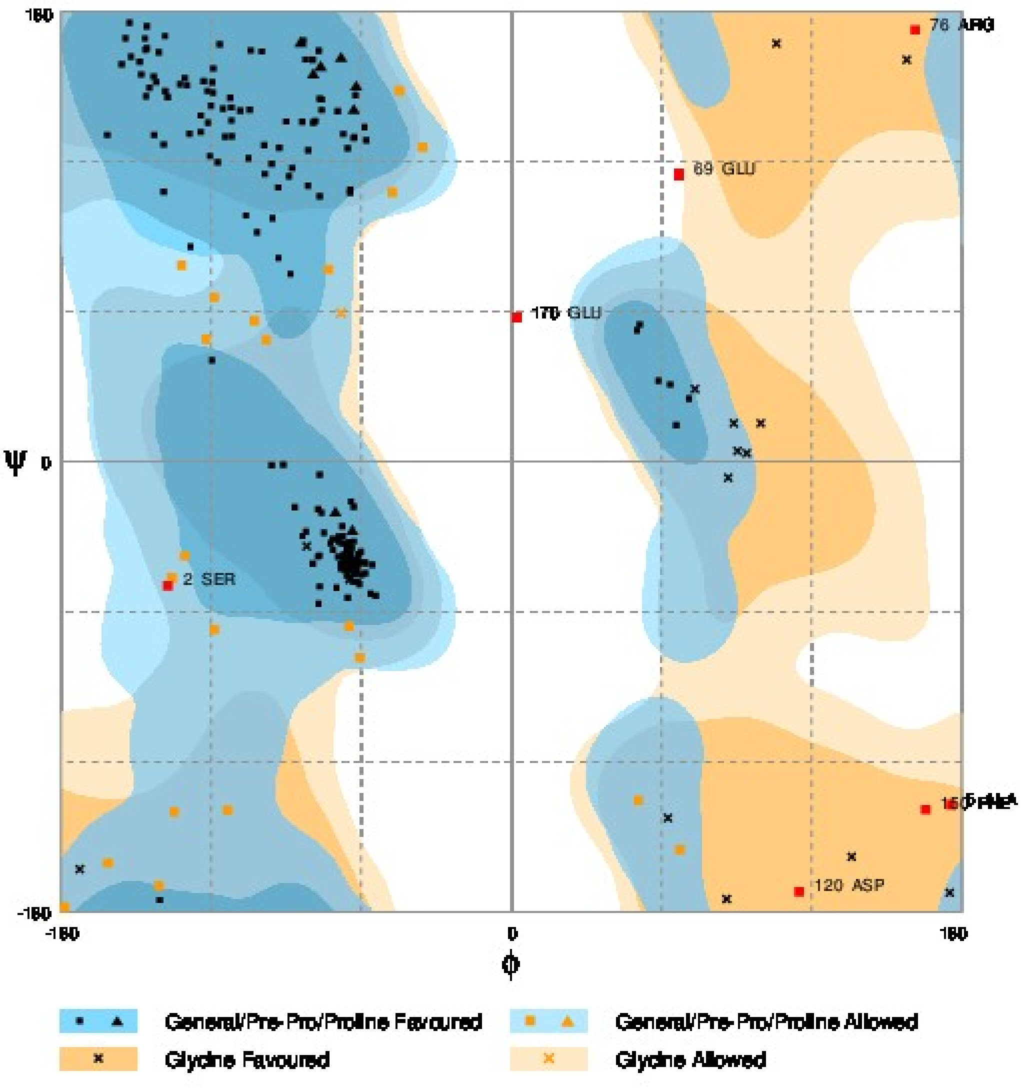


```
Number of residues in favoured region     (~98.0% expected)  :  196 ( 87.1%)
Number of residues in allowed region      ( ~2.0% expected)  :   22 (  9.8%)
Number of residues in outlier region                         :    7 (  3.1%)
```

TmL11

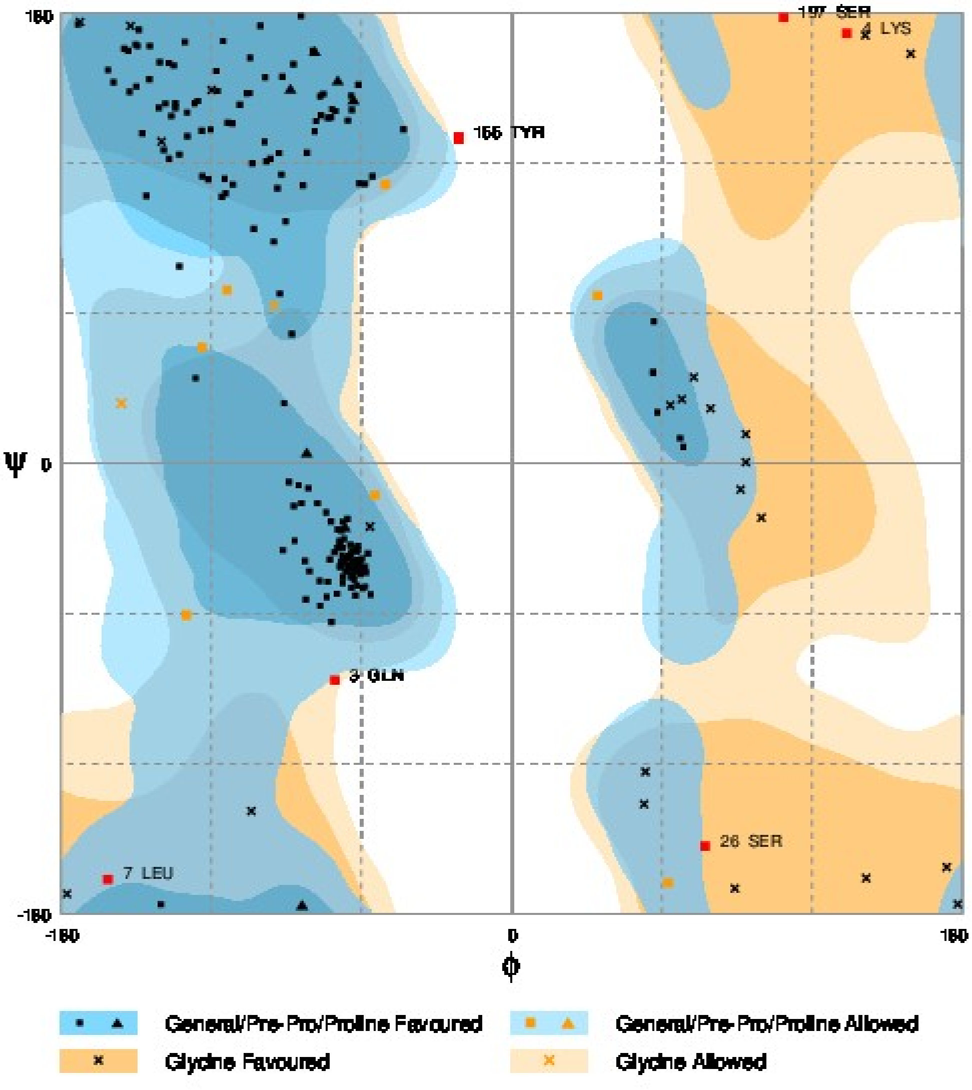


```
Number of residues in favoured region     (~98.0% expected)  :  209 ( 93.3%)
Number of residues in allowed region      ( ~2.0% expected)  :    9 (  4.0%)
Number of residues in outlier region                         :    6 (  2.7%)
```

TmL13

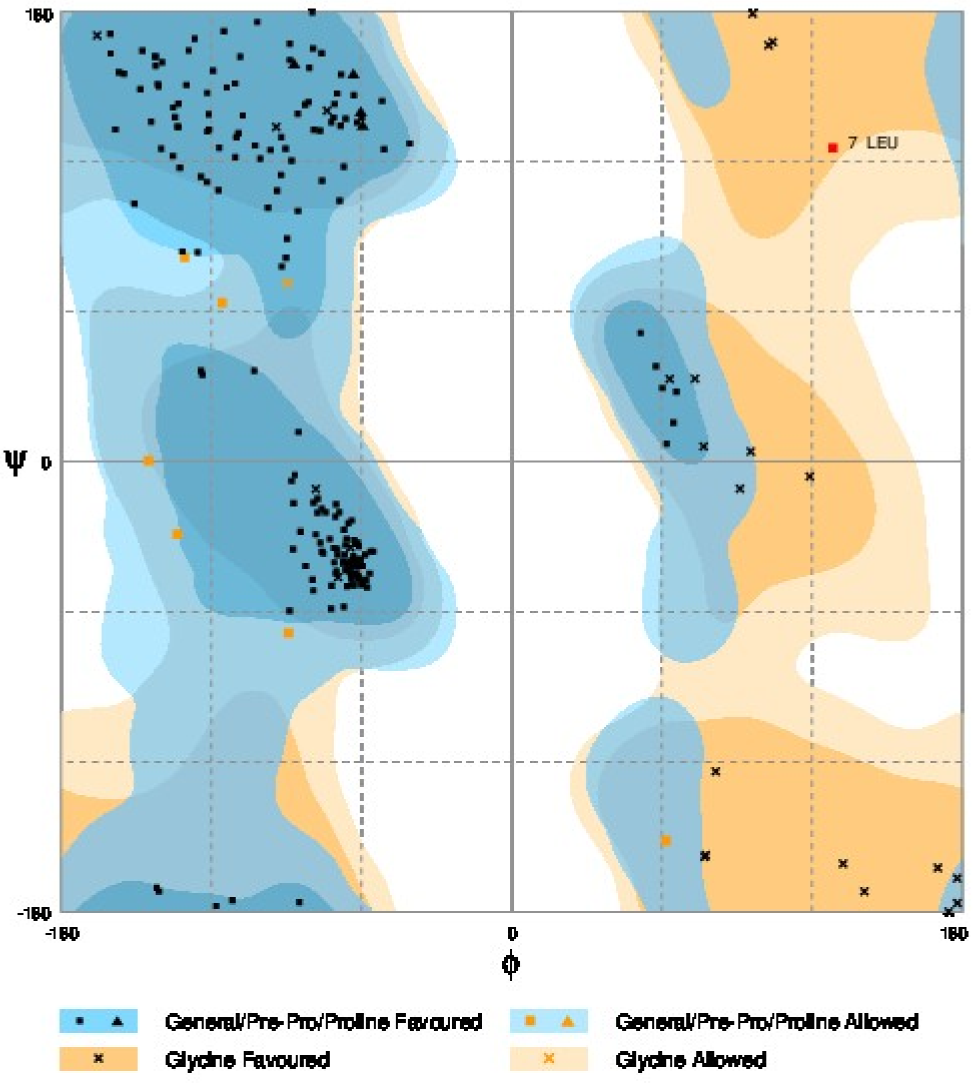


```
Number of residues in favoured region     (~98.0% expected)  :  210 ( 96.3%)
Number of residues in allowed region      ( ~2.0% expected)  :    7 (  3.2%)
Number of residues in outlier region                         :    1 (  0.5%)
```

TmB18

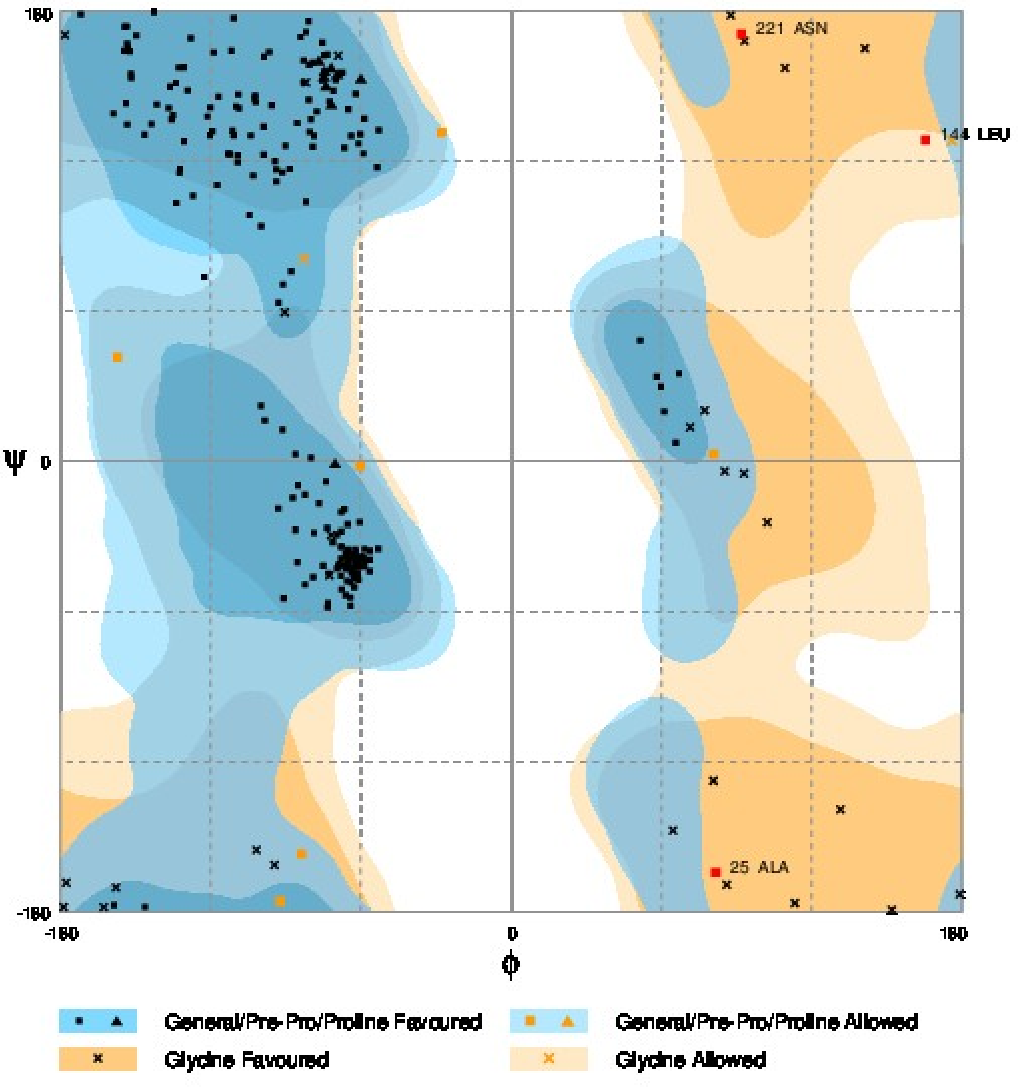


```
Number of residues in favoured region     (~98.0% expected)  :  239 ( 95.6%)
Number of residues in allowed region      ( ~2.0% expected)  :    8 (  3.2%)
Number of residues in outlier region                         :    3 (  1.2%)
```

TmB19

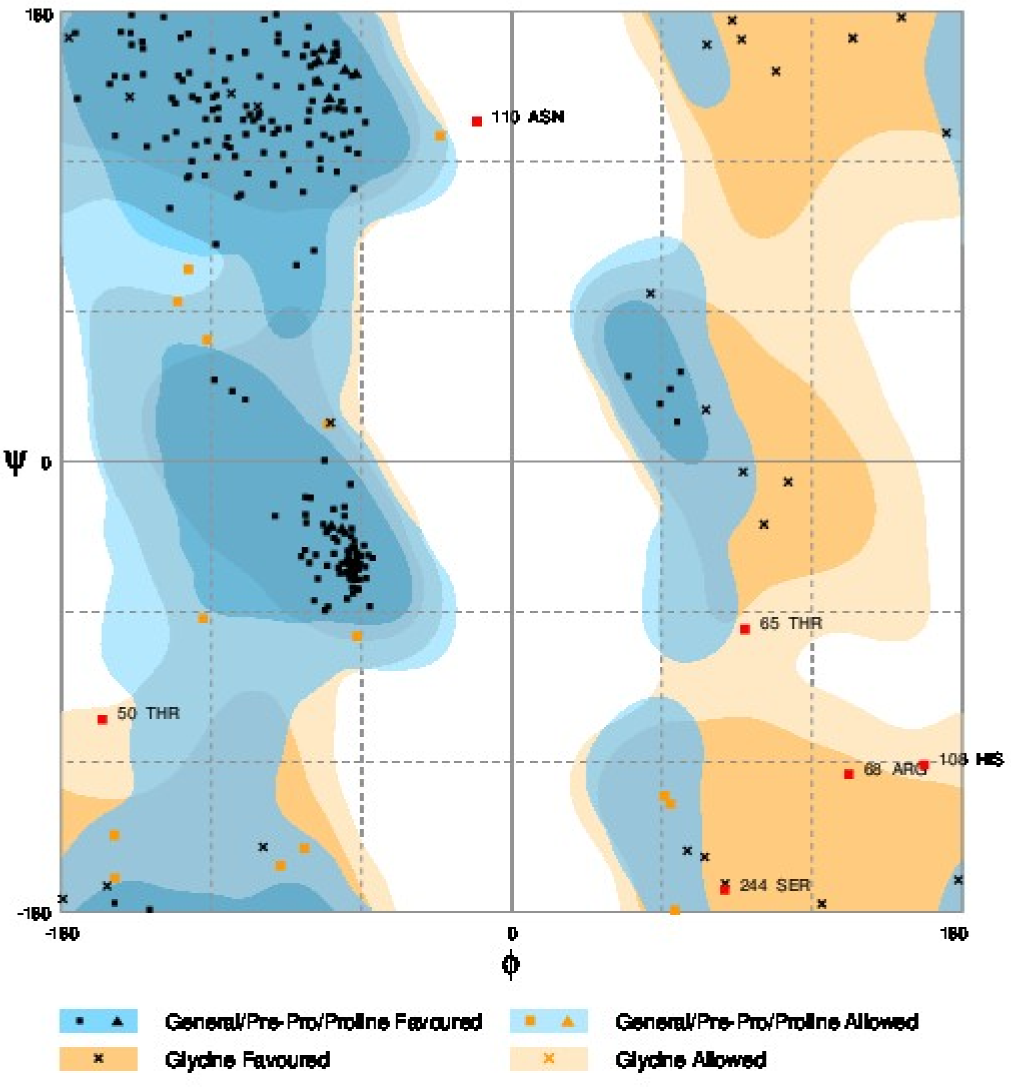


```
Number of residues in favoured region     (~98.0% expected)  :  244 ( 92.4%)
Number of residues in allowed region      ( ~2.0% expected)  :   14 (  5.3%)
Number of residues in outlier region                         :    6 (  2.3%)
```

TmB20

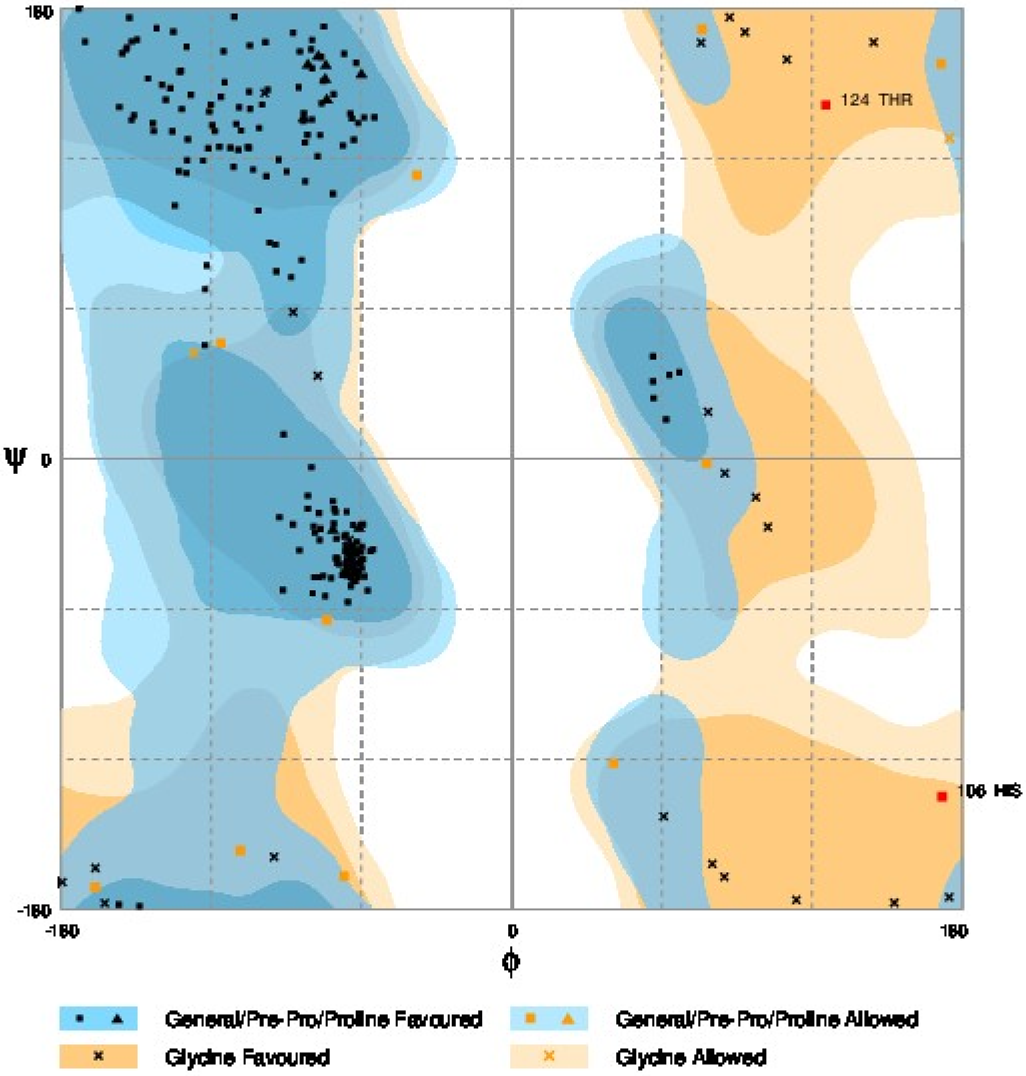


```
Number of residues in favoured region     (~98.0% expected)  :   223 ( 94.1%)
Number of residues in allowed region      ( ~2.0% expected)  :    12 (  5.1%)
Number of residues in outlier region                         :     2 (  0.8%)
```

TmB22

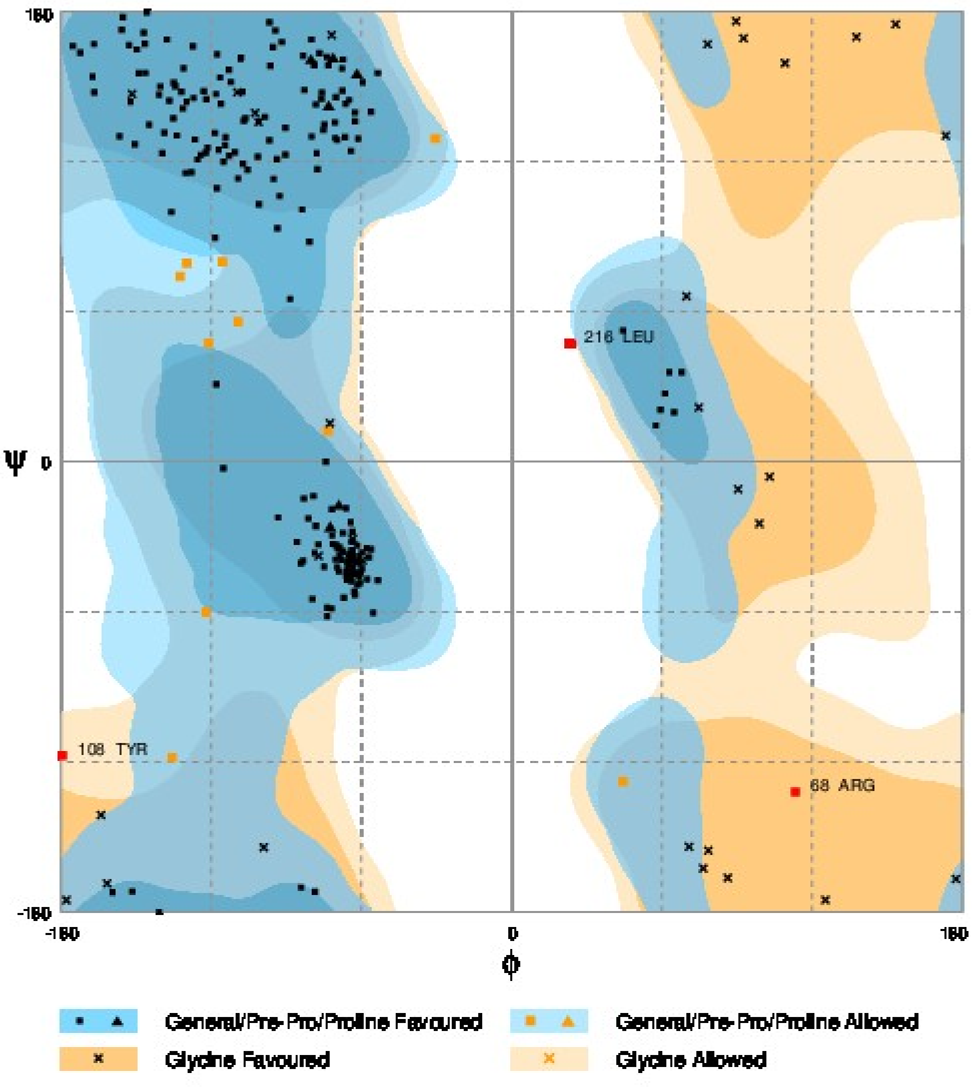


```
Number of residues in favoured region     (~98.0% expected)  :  252 ( 95.1%)
Number of residues in allowed region      ( ~2.0% expected)  :   10 (  3.8%)
Number of residues in outlier region                         :    3 (  1.1%)
```

TmB28

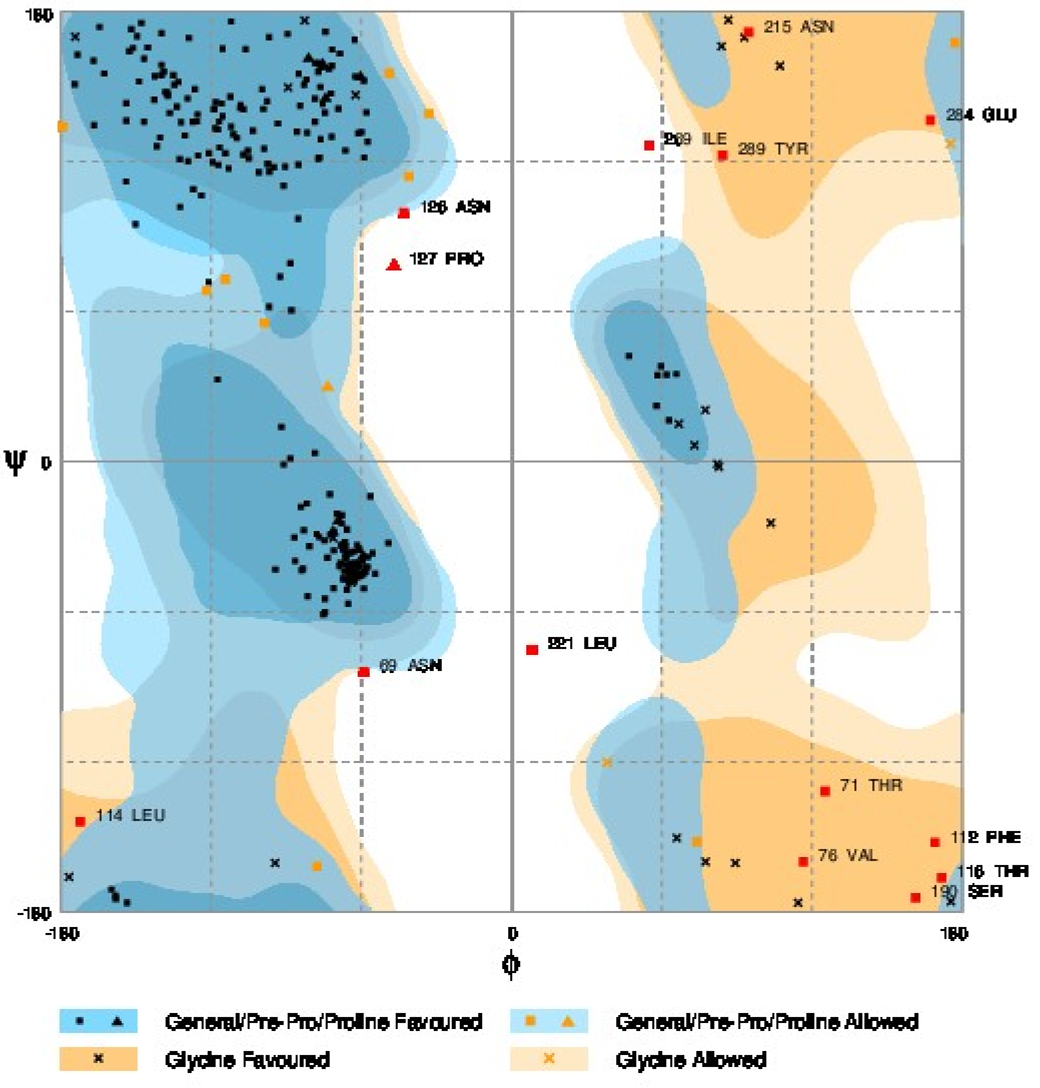


```
Number of residues in favoured region     (~98.0% expected)  :   265 ( 90.8%)
Number of residues in allowed region      ( ~2.0% expected)  :    13 (  4.5%)
Number of residues in outlier region                         :    14 (  4.8%)
```

TmB33

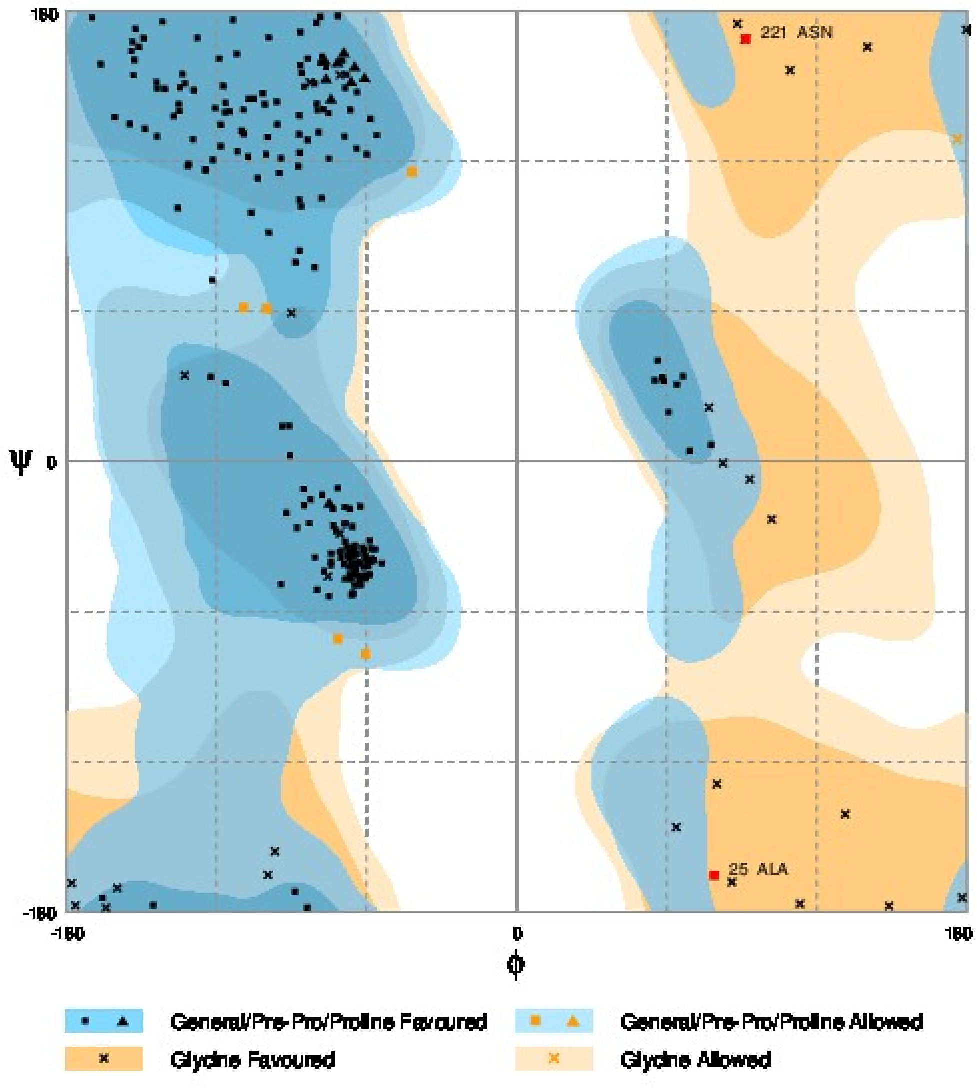


```
Number of residues in favoured region     (~98.0% expected)  :  242 ( 96.8%)
Number of residues in allowed region      ( ~2.0% expected)  :    6 (  2.4%)
Number of residues in outlier region                         :    2 (  0.8%)
```
