# Supplementary material for: Functional analysis of C1 family cysteine peptidases in the larval gut of Тenebrio molitor and Tribolium castaneum
Source: BMC Genomics. 2015 Feb 14;16(1):75. doi: 10.1186/s12864-015-1306-x (PMC4336737; doi:10.1186/s12864-015-1306-x)

A. 3QJ3 vs TmL5a (mature cathepsin)

In 3QJ3 – catalytic cysteine is changed to serine

##	Q-score	P-score	Z-score	RMSD	Nalgn	Nsse	Ngaps	Seq-%	Nmd	Nres-Q	Nsse-Q	Nres-T	Nsse-T	Query	Target
1	0.6929	26.95	17.25	0.199	222	12	0	0.9955	0	319	20	222	14	3qj3:A	TmL5a

```
H·  A:SER 129       0.04 | H-   CYS  31
S+  A:HIS 267  ::·  0.05 | S+   HIS 169
 +  A:ASN 287  ::·  0.05 |  +   ASN 189
```

B. 3QT4 vs TmL13a (mature cathepsin)

In 3QT4– catalytic cysteine is changed to serine

##	Q-score	P-score	Z-score	RMSD	Nalgn	Nsse	Ngaps	Seq-%	Nmd	Nres-Q	Nsse-Q	Nres-T	Nsse-T	Query	Target
1	0.7213	37.53	18.62	0.173	220	13	0	0.9909	0	304	20	220	13	3qt4:A	TmL13a

H· A:SER 122 0.05 | H- CYS 30

S+ A:HIS 259 ::· 0.04 | S+ HIS 167

+ A:ASN 279 ::· 0.02 | + ASN 187

C. 3OF8 (mature cathepsin L human) vs 1CS8 (procathepsin L human)

##	Q-score	P-score	Z-score	RMSD	Nalgn	Nsse	Ngaps	Seq-%	Nmd	Nres-Q	Nsse-Q	Nres-T	Nsse-T	Query	Target
1	0.6522	31.92	17.18	0.469	216	14	1	0.9907	0	221	14	316	19	3of8:A	1cs8:A

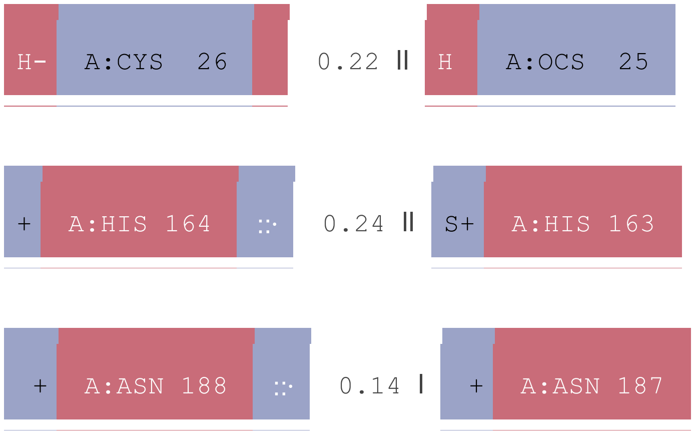

Supplement: Additional file 7: Figure S6. — The models of mature peptidases from T. molitor (A, TmL5; B, TmL13) were compared with crystal structures of corresponding mutant procathepsins 3QJ3 and 3QT4 [41], respectively, using PDBeFold [39]. C, the comparison of mature (3OF8) vs procathepsin L (1CS8) from H. sapiens. [file 12864_2015_1306_MOESM7_ESM.pdf]
